# Efficacy and safety of a modified sound therapy for patients with subjective tinnitus (MOST): a multicentre, double-blind, randomised controlled trial

**DOI:** 10.1016/j.eclinm.2025.103671

**Published:** 2025-12-08

**Authors:** Dongmei Tang, Dantong Gu, Jiamin Gong, Guangyu Liu, Lei Zhou, Aqiang Dai, Yan Huo, Pengfei Guan, Jianning Zhang, Xinsheng Huang, Yunfeng Wang, Shan Sun, Huawei Li

**Affiliations:** a ENT Institute and Department of ENT Institute and Otorhinolaryngology, Tinnitus Hyperacusis Center, Eye & ENT Hospital, State Key Laboratory of Medical Neurobiology, NHC Key Laboratory of Hearing Medicine Research, Fudan University, Shanghai, 200032, China; b Department of Otolaryngology, Yueyang Hospital of Integrative Chinese & Western Medicine Affiliated to Shanghai University of Traditional Chinese Medicine, 110 Ganhe Rd, Shanghai, China; c Department of Otorhinolaryngology-Head and Neck Surgery, Zhongshan Hospital, Fudan University, 180 Fenglin Road, Shanghai, 200031, China; d Institutes of Biomedical Sciences, Fudan University, Shanghai, 200032, China; e The Institutes of Brain Science and the Collaborative Innovation Center for Brain Science, Fudan University, Shanghai, 200032, China

**Keywords:** Chronic subjective tinnitus, Sound therapy, Randomised controlled trial, Multicenter trial, Sustained effect

## Abstract

**Background:**

Tinnitus is a common and often debilitating auditory condition with limited treatment options. While sound therapy is widely used, robust evidence from long-term randomised trials is scarce. We aimed to evaluate the 9-month efficacy and 3-month posttreatment durability of four sound therapies for adults with chronic subjective tinnitus and identify predictors of response.

**Methods:**

In this multicentre, double-blind, randomised controlled clinical trial, participants (aged 18–80 years) with chronic subjective tinnitus from three academic hospitals in China were included. Participants were randomly 1:1:1:1 assigned to receive one of four daily 2-h interventions: unmodified music (UM), UM plus pitch-centered narrowband noise (UM + NBN), high-frequency–enhanced music (HFEM), or digital frequency-customised relieving sound (DFCRS). Primary outcome was tinnitus severity assessed by Tinnitus Handicap Inventory (THI). Assessments occurred at baseline, 1, 2, 3, 6, and 9 months, with a 3-month posttreatment follow-up. Two prespecified primary endpoints were defined: (a) complete remission, operationalised as a THI score of 0 at any follow-up visit within the 9-month period. Participants achieving this endpoint were considered clinically cured, and sound therapy was discontinued; or (b) if complete remission was not achieved by the end of 9 months, the magnitude of improvement was defined as the change in THI score from baseline to the 9-month endpoint. The primary analysis followed the intention-to-treat (ITT) principle, This trial is registered with the Chinese Clinical Trial Registry, ChiCTR2000039007.

**Findings:**

Between May 14, 2021, and November 30, 2022, 440 participants (median age 45 years [IQR, 35–56]; 222/440 [50·5%] male; median tinnitus duration 13 months [IQR, 7–36]) were enrolled and randomly assigned (UM, n = 111; UM + NBN, n = 110; HFEM, n = 108; DFCRS, n = 111). Baseline characteristics were balanced between the groups. Only one participant in the HFEM group achieved complete remission, with a THI score of 0 at the 6-month follow-up. THI scores significantly decreased over time in all groups (median 50·00 [IQR 36·00–62·00]) at baseline to 9-month follow-up (35·00 [24·00–48·00]; *p* < 0·0001), with effects sustained posttreatment. Significant group × time interactions occurred (UM: F_(5, 618)_ = 11·45; UM + NBN: F_(5, 605)_ = 7·17; HFEM: F_(5, 599)_ = 8·3; DFCRS: F_(5, 619)_ = 12·65; all *p* < 0·0001) in all arms. DFCRS demonstrated superior efficacy (parameter estimate −4·37, 95% CI −6·25 to −2·48; *p* < 0·0001), when compared to UM as reference. No adverse events were reported in any group.

**Interpretation:**

In this exploratory trial, personalised acoustic therapy may provide promising efficacy for chronic tinnitus. Although interpretation is tempered by the absence of a blank control arm and objective adherence monitoring, these limitations highlight opportunities for future studies to refine methods and validate treatment benefits more robustly.

**Funding:**

The Ministry of Science and Technology, the Shanghai Shenkang Development Centre, the 10.13039/501100003399Shanghai Science and Technology Committee, and the 10.13039/501100001809National Natural Science Foundation of China.

**Research in context**

**Evidence before this study**

Tinnitus affects approximately 10–15% of the adult population worldwide and represents a major clinical challenge due to the absence of consistently effective therapies. We searched PubMed and Embase up to January 31, 2024 using terms including “tinnitus,” “sound therapy,” “music therapy,” “randomized trial,” “notched music,” and “frequency-customization.” Previous studies suggested that sound therapy may relieve tinnitus by reducing tinnitus-related distress and altering abnormal auditory processing. However, the evidence base has been limited by small sample sizes, heterogeneous interventions, short follow-up durations, and few rigorously conducted randomized controlled trials. Notched music and noise-based approaches have shown modest and sometimes inconsistent effects. To date, no large multicenter, double-blind trials have comprehensively compared different sound therapy strategies or examined the durability of effects beyond the treatment period.

**Added value of this study**

This multicenter, double-blind, randomized controlled trial enrolled 440 patients with chronic subjective tinnitus and systematically compared four sound therapy approaches over a 9-month intervention period with an additional 3-month post-treatment follow-up. The study demonstrates that all sound therapies produced clinically meaningful improvement in tinnitus severity, but that digital frequency-customized relieving sound (DFCRS) achieved the greatest and most durable reductions in Tinnitus Handicap Inventory scores, as well as improvements in anxiety, sleep, and tinnitus loudness. Importantly, patient characteristics—such as age, tinnitus duration, location (bilateral vs unilateral), and type (intermittent vs persistent)—were identified as predictors of treatment response. By providing parameter estimates, subgroup analyses, and detailed statistical reporting, this study addresses several methodological shortcomings of prior research, thereby strengthening the clinical evidence for sound therapy in tinnitus.

**Implications of all the available evidence**

The findings indicate that long-term sound therapy can yield sustained symptom relief for chronic tinnitus, and that individualized, frequency-targeted interventions such as DFCRS provide greater therapeutic benefit than unmodified music or conventional noise-based approaches. The durability of treatment effects suggests that sound therapy may promote lasting auditory plasticity rather than transient symptom masking. Moreover, the identification of patient-specific predictors of response underscores the potential for a more personalized approach to tinnitus management. However, the absence of a no-treatment control arm limits the ability to fully disentangle therapeutic effects from placebo responses or natural fluctuations in symptoms, and the lack of objective monitoring of treatment adherence restricts conclusions about dose–response relationships. Future studies incorporating digital tracking and appropriate control groups will be critical to validate and extend these findings. Collectively, the available evidence supports DFCRS as a promising, noninvasive, and scalable therapy for managing chronic subjective tinnitus, while also highlighting methodological considerations that must be addressed to strengthen causal inference and optimize personalized treatment strategies.

## Introduction

Tinnitus, commonly characterised as a persistent ringing or noise in the ears in the absence of external sound, is a prevalent auditory condition that affects a significant portion of the global population.^1^^,^^2^ The subjective nature of tinnitus, combined with its diverse etiological factors, makes it a difficult condition to manage and treat effectively.^3^ Current therapeutic strategies vary widely, but sound therapy has emerged as a particularly promising approach due to its non-invasive nature and its potential to directly address the auditory symptoms of tinnitus.^4^

The perception of tinnitus is believed to be the result of neural changes within the neural system, which may be influenced by auditory input, cognitive processes, and emotional state.^5^ Sound therapy aims to provide auditory stimulation or noise masking that can potentially recalibrate the neural mechanisms responsible for tinnitus perception, thereby reducing its severity or the distress associated with it.^6^ Various forms of sound therapy have been proposed, ranging from broad-spectrum noise generators to customised sound therapies tailored to individual auditory profiles.^7^ In our previous studies,^8^^,^^9^ we developed a method of digital frequency-customised relieving sound (DFCRS), also referred to as modified tinnitus-relieving sound,^8^^,^^9^ which involves creating a sound profile that exactly modulating specific frequency bands of music based on the pitch of the tinnitus perceived by the individual. By aligning the therapeutic sound with the specific characteristics of the subjectively perceived tinnitus, it is possible to effectively alter the brain's perception of the tinnitus, leading to potential long-term habituation or attenuation of the intrusive noise.^10^

Despite the popularity of sound therapy in clinical settings, there remains significant variability in the treatment outcomes, which may be attributable to the non-standardised nature of therapy application and a lack of understanding regarding which types of sound therapy are most effective for specific patient demographics and tinnitus characteristics. In our earlier small-sample, short-term, non-randomised study, DFCRS demonstrated positive therapeutic effects,^11^ however, we also observed that baseline tinnitus severity, as measured by the tinnitus handicap inventory (THI),^12^ significantly influenced treatment outcomes.^13^ These findings underscored the need for a larger, long-term, randomised controlled trial to account for baseline variability and enhance the generalisability of results.

To address these limitations, we conducted the modified sound therapy for chronic subjective tinnitus (MOST) trial—a multicenter, randomised, controlled study designed to evaluate the efficacy of four sound therapy strategies: unmodified music (UM), UM combined with narrowband noise (UM + NBN), high-frequency-enhanced music (HFEM), and DFCRS. The trial aimed not only to determine the most effective acoustic intervention but also to explore how individual tinnitus characteristics and demographic factors modulate therapeutic response. Ultimately, this study seeks to inform a more systematic, personalised approach to sound therapy in tinnitus management.

## Methods

### Study design

This multi-arm, parallel-group, double-blind, randomised controlled trial was reported according to the Reporting of Multi-Arm Parallel-Group Randomised Trials Extension of the CONSORT 2010 Statement and was conducted in three outpatient medical clinics in Shanghai, China, in accordance with current good clinical practices and applicable regulations. The study was designed to compare each of the modified sound arms to the UM (negative control) arm and the UM + NBN (standard control) arm using the tinnitus handicap inventory (THI)^12^ over a 9-month period with sound therapy and 3 additional months without sound therapy. The end of the trial (=9 months) was defined as the primary endpoint.

The study protocol followed the World Medical Association's Declaration of Helsinki and was approved by the Institutional Review Board of the Ethics Committee of the Eye and ENT Hospital of Fudan University (No. 2017048, and the revised version is 2017048-1 and 2017048-2, Shanghai, China; Supplementary Appendix 1) and was registered on https://www.chictr.org.cn/ (ChiCTR2000039007, October 13, 2020). The protocol was also approved by the Institutional Review Boards of all participating sites (B2021-396R, SHSK2020001). All participants or their representatives provided written informed consent before participation in the trial.

### Participants

Adults with chronic subjective tinnitus were eligible to participate if they met the following criteria: (1) age 18–80 years old, (2) a duration of tinnitus ≥3 months, (3) normal middle ear function, with pure-tone average (PTA) (0·5, 1, 2 kHz) of the worse ear less than 55 dB HL, and (4) the ability to communicate with others in Mandarin.

Exclusion criteria included: (1) vascular pulsatile tinnitus or objective tinnitus, (2) a major health problem that affected or prevented them from participating in the study or cooperating with the follow-up, (3) other diseases that might induce or influence tinnitus, (4) severe mental diseases, or (5) participating in another research project. Data collected from all patients enrolled in the study included age, sex, PTA threshold, psychoacoustic measures of tinnitus, and history of tinnitus at baseline, including the duration, the location, the tone, and the type of tinnitus.

### Randomisation and masking

Eligible participants were randomly assigned in a 1:1:1:1 ratio to each arm. The randomisation process was conducted by an independent statistician using block randomisation with variable block sizes. Before randomisation, only the study coordinator was aware of the treatment assignments, while the data assessors remained blinded to the treatment allocation. Both the participants and the clinicians were blinded to the study treatments. Detailed information is provided in the study protocol and statistics analysis (Supplementary Appendix 1 & 2).

### Procedures

Eligible participants underwent a baseline assessment (T0), which included PTA to determine hearing thresholds, detailed clinical history of tinnitus, and completion of standardised self-report questionnaires: the THI, the Hospital Anxiety and Depression Scale (HADS-A for anxiety, HADS-D for depression),^14^ the Athens Insomnia Scale (AIS-8),^15^ and two 0–10 point Visual Analog Scale (VAS) assessing tinnitus loudness and tinnitus-related distress.^16^

Following the T0 evaluation, participants were randomly assigned to one of four intervention arms for a 9-month sound therapy program. All participants received both oral and written instructions on how to access and engage with their assigned intervention. Monthly reminders and follow-up messages were sent by the research team to support engagement and retention. Follow-up assessments were conducted at 1 month (T1), 2 months (T2), 3 months (T3), 6 months (T4), 9 months (T5), and an extension follow-up at 12 months (T6) (Figure S1).

All participants were instructed to complete at least 2 h of sound therapy daily using dedicated MP3 devices. No specific time of day was prescribed, and exposure could be distributed flexible according to individual routines (e.g., four 30-min sessions). Additional use was permitted and encouraged during periods when tinnitus symptoms were particularly bothersome. To promote compliance, several supportive strategies were implemented: pre-treatment educational sessions, individualised instruction on music listening, reinforcement during scheduled follow up visits, and regular telephone counseling. Examples of acoustic treatment for each arm were provided (Supplementary Audio 1–4).

Participants were assigned to one of the following four intervention arms (Figure S2):

1) UM arm: Participants in this group listened to unmodulated pure music.
2) UM + NBN arm: The sound used with this group was unmodulated pure music intermixed with 1/3 octave NBN centered on the dominant pitch of the tinnitus.
3) HFEM arm: The modulated frequency range was 6000–9000 Hz and the enhanced intensity was 10 dB in this group, regardless of the pitch of the tinnitus.
4) DFCRS arm: The soothing music was modulated according to our particular algorithm in which two specific frequency bands of 1/3 octave intervals below and above the tinnitus pitch were confirmed, and the two specified bands were dynamically increased in steps of 10 dB.^8^^,^^9^^,^^17^ The enhancement of sound energy in frequency bands adjacent to the tinnitus-matched frequency can suppress neural activity within the tinnitus-corresponding region through lateral inhibition, thereby reducing perceived tinnitus-related activity. Because the frequency and intensity of music naturally fluctuate over time, the frequency bands emphasised by this modulation strategy also exhibit dynamic, time-varying characteristics. In addition to these neurophysiological effects, the soothing and pleasant qualities of music facilitate relaxation and redirect attention, which may further contribute to the therapeutic efficacy for tinnitus management.^17^

To mitigate potential operator-related variability in this multicenter study, standardised procedures were established prior to data collection. All responsible for tinnitus characterisation, pure-tone audiometry, and questionnaire administration underwent unified training at offline meetings across all participating sites. This training ensured adherence to a consistent study protocol, harmonised testing procedures, and uniform approaches to patient instruction. In addition, roles and responsibilities for staff were clearly defined at each center to further enhance consistency. To minimise technical variation, standardised equipment was employed, including the use of identical MP3 devices for sound therapy across sites. Collectively, these measures were implemented to reduce site-specific experimental bias and strengthen the reliability of the data.

### Outcomes

The primary outcome was tinnitus severity, assessed with THI (range: 0–100) over the 9-month treatment and follow-up period. Two prespecified primary endpoints were defined: (a) complete remission, operationalised as a THI score of 0 at any follow-up visit within the 9-month period. Participants achieving this endpoint were considered clinically cured, and sound therapy was discontinued; or (b) if complete remission was not achieved by the end of 9 months, the magnitude of improvement was defined as the change in THI score from baseline to the 9-month endpoint. THI scores were collected at baseline and at each scheduled follow-up assessment throughout the study period.

Secondary outcomes included the scales assessing the subjective loudness and annoyance of tinnitus using a 0–10 points VAS-loudness and VAS- annoyance measure, the anxiety and depression caused by the tinnitus using the HADS-A and HADS-D, respectively, and the influence of tinnitus on sleep as measured by the AIS-8.

Participants were able to report multiple events simultaneously, and adverse events were assessed via open-ended questions at all follow-up visits, including their respective incidence and type. No adverse events were reported in this trial, and therefore no interim analyses were performed.

### Statistical analysis

The sample size was calculated using generalised estimating equation (GEE) tests for the slope of multiple groups in a repeated measures design (continuous outcome) using the NCSS software (Kaysville, UT, USA). A total of 400 participants, divided equally among the four arms and measured at all six time points, provided a power of 0·90 to detect significant differences in group slopes at a significance level of 0·05. Group slopes under the alternative hypothesis were set at −0·23, −0·24, −0·27, and −0·30, with a residual standard deviation of 0·172. The time points were proportional to the total study duration: 0, 0·2, 0·4, 0·6, 0·8, and 1 for T0–T5, respectively.

Demographics and baseline characteristics are summarised by treatment arm, and categorical variables are presented as frequencies and percentages, while continuous variables are expressed as means ± standard deviations (for normally distributed data) or as medians and interquartile ranges (IQR) (for non-normally distributed data). Normality was assessed graphically using the Shapiro–Wilk test. Differences between groups were analysed with chi-square or Fisher's exact tests for categorical variables, by one-way ANOVA for normally distributed continuous variables, and by Mann–Whitney U-tests for non-normally distributed continuous variables. Means and 95% confidence intervals (CIs) were calculated for the THI, HADS-A, HADS-D, AIS, and VAS scores at baseline and at each follow-up. GEE was used to analyse changes in THI scores over time, adjusting for covariates such as age, sex, tinnitus duration, tinnitus characteristics.

The primary analysis followed the intention-to-treat (ITT) principle, including all 440 randomised participants analysed according to their assigned treatment arms. The mechanism of missing data was formally assessed prior to imputation. Little's test for Missing Completely at Random (MCAR) was statistically significant, thereby rejecting the MCAR hypothesis. To further distinguish between Missing at Random (MAR) and Missing Not at Random (MNAR), logistic regression models were fitted to examine whether the probability of missingness at each follow-up could be fully observed baseline variables. The absence of significant associations supported the plausibility of the MAR mechanism. Consequently, multiple imputation under the MAR assumption was performed using Predictive Mean Matching (PMM) implemented in the R package mice, with 50 imputed datasets created. The imputation model incorporated all longitudinal outcome variables, treatment group, relevant covariates, and auxiliary baseline variables in order to strengthen the robustness of the MAR assumption. An extended follow-up analysis assessed outcomes at the primary endpoint and again at 12 months (an extension of 3 months following the treatment phase concluded). THI scores were categorised into severity levels. To visualise the longitudinal flow of individual participants' THI severity states, a Sankey diagram was generated specifically for the subgroup of participants with complete THI data at 0, 3, 9, and 12 months. This diagram depicts the frequencies (counts) and proportions of participants moving between severity categories across these four time points.

To evaluate the robustness of the findings, sensitivity analyses were conducted. Different GEE model specifications (null, simplified, and fully adjusted models) were compared. Both intention-to-treat (with multiple imputation) and per-protocol (PP, without imputation) analyses were also performed. Results were consistent across approaches, supporting the stability of the main conclusions.

All statistical tests were two-tailed, with the significance level set at α = 0·05. All analyses were performed using R software (version 4·2·3). R packages “geepack”, “lubridate”, “tidyr”, “dplyr”, and “mice” were used in our analysis.

### Role of the funding source

The funder of the study had no role in study design, data collection, data analysis, data interpretation, or writing of the report. DM Tang had full access to the data in the study, and HW Li had final responsibility for the decision to submit for publication.

## Results

### Baseline characteristics

**Fig. 1:**
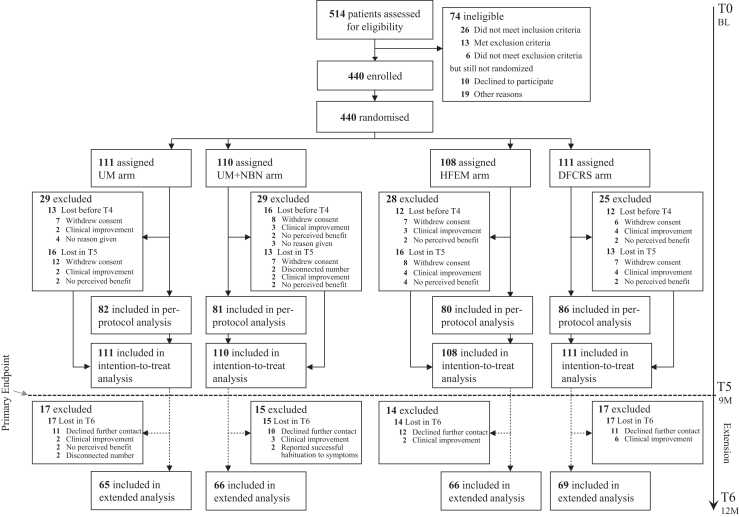
**CONSORT diagram of the study enrollment flow.** The diagram illustrates the study enrollment process following the CONSORT (Consolidated Standards of Reporting Trials) guidelines. Abbreviations: UM, unmodified music; UM + NBN, unmodified music combined with narrowband noise; HFEM, high frequency-enhanced music; DFCRS, digital frequency-customised relieving sound.

The study enrolled 440 participants with chronic subjective tinnitus across three Shanghai medical centers between May 14, 2021 and November 30, 2023. Participants were randomly allocated to four intervention groups: UM (n = 111), UM + NBN (n = 110), HFEM (n = 108), and DFCRS (n = 111) (Fig. 1). The cohort had a median age of 45 years (interquartile range [IQR] 35–56) with balanced sex distribution (222/440 males [50·5%]). Median tinnitus duration was 13 months (IQR 7–36).

Comprehensive baseline assessment demonstrated no significant differences among treatment groups in demographic characteristics, tinnitus parameters (duration, laterality, pitch matching, loudness, type), audiometric profiles (PTA in both ears), or multidimensional tinnitus severity measures including THI, HADS, AIS-8, and VAS scores (Table 1).

**Table 1:** Baseline characteristics by group.

	Level	Overall (N = 440)	UM (N = 111)	UM + NBN (N = 110)	HFEM (N = 108)	DFCRS (N = 111)	*p-*value
Research center	EENT Hospital	199 (45·2)	50 (45·0)	50 (45·5)	49 (45·4)	50 (45·0)	1·00
	YY Hospital	180 (40·9)	45 (40·5)	45 (40·9)	45 (41·7)	45 (40·5)	
	ZS Hospital	61 (13·9)	16 (14·4)	15 (13·6)	14 (13·0)	16 (14·4)	
Sex	Male	222 (50·5)	58 (52·3)	56 (50·9)	53 (49·1)	55 (49·5)	0·97
	Female	218 (49·5)	53 (47·7)	54 (49·1)	55 (50·9)	56 (50·5)	
Age (years)		45·00 [35·00, 56·00]	45·00 [38·00, 57·50]	43·00 [32·00, 53·00]	45·50 [35·00, 57·00]	44·00 [37·00, 55·50]	0·29
Tinnitus duration		13·00 [7·00, 36·00]	12·00 [6·00, 36·00]	15·50 [7·25, 39·00]	12·50 [7·00, 36·00]	16·00 [8·00, 29·00]	0·63
Tinnitus location	Unilateral	278 (63·2)	71 (64·0)	62 (56·4)	75 (69·4)	70 (63·1)	0·61
	Bilateral	151 (34·3)	38 (34·2)	45 (40·9)	30 (27·8)	38 (34·2)	
	Head	11 (2·5)	2 (1·8)	3 (2·7)	3 (2·8)	3 (2·7)	
Tinnitus tone	High pitch	332 (75·5)	85 (76·6)	84 (76·4)	81 (75·0)	82 (73·9)	0·79
	Low pitch	102 (23·2)	24 (21·6)	25 (22·7)	27 (25·0)	26 (23·4)	
	Noise-like	6 (1·4)	2 (1·8)	1 (0·9)	0 (0·0)	3 (2·7)	
Tinnitus type	Intermittent	59 (13·4)	14 (12·6)	12 (10·9)	10 (9·3)	23 (20·7)	0·061
	Persistent	381 (86·6)	97 (87·4)	98 (89·1)	98 (90·7)	88 (79·3)	
Average PTA∗ (AC, dB HL)	Right	21·25 [15·00, 35·00]	21·25 [15·62, 34·38]	21·25 [15·00, 35·00]	21·25 [13·75, 35·62]	21·25 [13·75, 31·88]	0·97
	Left	22·50 [14·38, 33·75]	22·50 [15·00, 33·75]	21·25 [13·75, 37·50]	25·00 [13·75, 35·94]	20·00 [14·38, 31·25]	0·60
THI_BL_		50·00 [36·00, 62·00]	52·00 [35·00, 63·00]	50·00 [38·50, 61·50]	51·00 [38·00, 66·00]	46·00 [34·00, 62·00]	0·42
HADA_BL_-	Anxiety	6·00 [3·00, 8·00]	6·00 [4·00, 8·00]	5·00 [3·00, 7·00]	5·50 [3·00, 8·00]	5·00 [3·00, 7·50]	0·77
HADS_BL_-	Depression	5·00 [3·00, 6·00]	5·00 [3·00, 6·00]	4·00 [3·00, 6·00]	4·00 [3·00, 6·00]	4·00 [3·00, 6·00]	0·45
AIS_BL_		7·00 [5·00, 9·00]	7·00 [5·00, 10·00]	6·00 [5·00, 9·00]	7·50 [5·00, 9·25]	7·00 [5·00, 8·50]	0·55
VAS_BL_	Loudness	5·00 [4·00, 6·00]	5·00 [4·00, 6·00]	5·00 [4·00, 6·00]	5·00 [4·00, 7·00]	5·00 [4·00, 6·00]	0·12
	Annoyance	5·00 [3·75, 6·00]	5·00 [3·00, 5·00]	5·00 [4·00, 6·00]	5·00 [3·00, 6·00]	5·00 [3·00, 6·00]	0·58
Tinnitus PM (Hz)	Left						0·75
	Low∗	66 (23·0)	18 (24·0)	18 (24·3)	18 (26·5)	12 (17·1)	
	Mid∗	68 (23·7)	21 (28·0)	17 (23·0)	13 (19·1)	17 (24·3)	
	High∗	153 (53·3)	36 (48·0)	39 (52·7)	37 (54·4)	41 (58·6)	
	Right						0·36
	Low	55 (21·3)	10 (16·4)	12 (18·2)	19 (31·1)	14 (20·0)	
	Mid	68 (26·4)	15 (24·6)	16 (24·2)	15 (24·6)	22 (31·4)	
	High	135 (52·3)	36 (59·0)	38 (57·6)	27 (44·3)	34 (48·6)	
Tinnitus loudness (dB SL)	Left	10·00 [7·00, 14·00]	9·00 [7·00, 13·00]	10·00 [7·00, 15·00]	11·00 [8·75, 13·25]	10·50 [7·25, 13·75]	0·68
	Right	10·00 [6·00, 13·00]	9·00 [5·00, 13·00]	10·00 [7·00, 15·00]	9·00 [6·50, 14·25]	10·00 [6·00, 13·75]	0·47

Data are median [IQR], mean (sd), or n (%). ∗ means the average pure tone hearing thresholds of 500, 1000, 2000, and 4000 Hz. Low∗ means < 1000 Hz; Mid∗ means 1000 Hz (inclusive) ∼ 4000 Hz (exclusive); High∗ means 4000 Hz (inclusive) ∼ 8000 Hz (inclusive).

Abbreviation: AC, air conduction; BL, baseline; HL, hearing level; PM, pitch match; PTA, pure tone audiometry; SL, sensation level. THI, Tinnitus Handicap Inventory; HADS, Hospital Anxiety and Depression Scale; AIS-8, Athens Insomnia Scale-8; and VAS, Visual Analog Scale.

Inter-center comparisons revealed homogeneous participant characteristics regarding demographics and most clinical measures across the three institutions. However, baseline VAS assessment showed statistically significant differences (*p* < 0·05), with participants at Zhongshan Hospital reporting lower tinnitus distress compared to the other two centers (Table S1). The distribution of intervention groups remained consistent across all research sites.

### Primary outcome

Acoustic therapy demonstrated significant efficacy in tinnitus management across all interventions. THI scores decreased substantially from baseline (median 50·00 [IQR 36·00–62·00]) to 9-month follow-up (35·00 [24·00–48·00]; *p* < 0·0001; Table S2). Because only one participant in the HFEM group achieved complete remission (THI = 0) at the 6-month follow-up, treatment efficacy was assessed using the change in THI scores from baseline rather than remission rates.

**Fig. 2:**
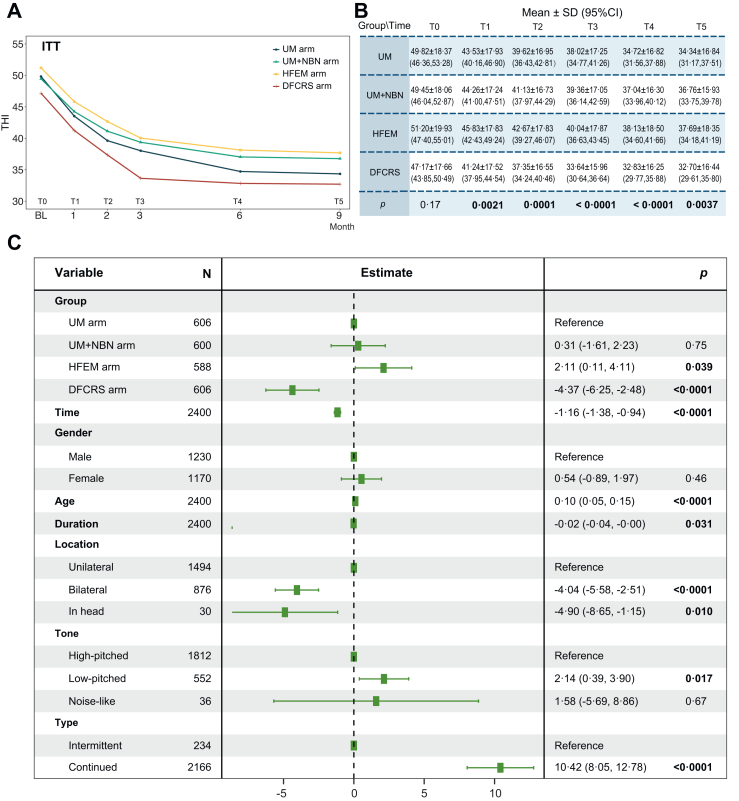
**Primary outcome measured by Tinnitus Handicap Inventory (THI) scores.** (A and B) THI scores at baseline and at the 1-month, 2-month, 3-month, 6-month, 9-month, and 12-month follow-ups in the intention-to-treat (ITT) analyses. (C) The Generalised Estimating Equation (GEE) model based on the intention-to-treat (ITT) analyses.

Analysis of treatment-specific effects revealed significant group × time interactions for THI scores in all arms. (UM: *F*_*(5, 618)*_ = 11·45; UM + NBN: *F*_*(5, 605)*_ = 7·17; HFEM: *F*_*(5, 599)*_ = 8·3; DFCRS: *F*_*(5, 619)*_ = 12·65; all *p* < 0·0001; Table S3). Each intervention showed progressively decreasing THI scores throughout the 9-month treatment period (Fig. 2A and B), confirming the therapeutic benefit of sustained sound exposure.

GEE modeling identified significant predictors of tinnitus treatment response using THI score reduction as the primary outcome measure (Fig. 2C, Table 2). Treatment modality significantly influenced outcomes when compared to UM as reference: DFCRS demonstrated superior efficacy (parameter estimate −4·37, 95% CI −6·25 to −2·48; *p* < 0·0001), while HFEM (2·11, 95% CI 0·11–4·11; *p* = 0·039) showed reduced effectiveness, and UM + NBN was comparable to UM (0·31, 95% CI −1·61 to 2·23; *p* = 0·75) (Fig. 2C).

**Table 2:** Generalised estimating equations model affecting the efficacy of tinnitus sound therapy (intention-to-treat).

Predictors	Null model			Simplified model			Adjusted model		
	Estimates	CI	*p*	Estimates	CI	*p*	Estimates	CI	*p*
(Intercept)	42·74	42·02–43·46	**<0·0001**	47·53	45·95–49·10	**<0·0001**	34·84	31·37–38·31	**<0·0001**
UM				Ref	Ref	Ref	Ref	Ref	Ref
UM + NBN				−0·43	−2·36–1·50	0·66	0·31	−1·61 to 2·23	0·75
Group									
HFEM				2·55	0·55–4·55	**0·012**	2·11	0·11–4·11	**0·039**
DFCRS				−4·87	−6·80 to −2·94	**<0·0001**	−4·37	−6·25 to −2·48	**<0·0001**
Time									
Time				−1·16	−1·39 to −0·94	**<0·0001**	−1·16	−1·38 to −0·94	**<0·0001**
Sex									
Male							Ref	Ref	Ref
Female							0·54	−0·89 to 1·97	0·46
Age									
Age							0·10	0·05–0·15	**<0·0001**
Duration									
Duration							−0·02	−0·04 to −0·00	**0·031**
Location									
Unilateral							Ref	Ref	Ref
Bilateral							−4·04	−5·58 to −2·51	**<0·0001**
Head							−4·9	−8·65 to −1·15	**0·010**
Tone									
High pitched							Ref	Ref	Ref
Low pitched							2·14	0·39–3·90	**0·017**
Noise-like							1·58	−5·69 to 8·86	0·67
Type									
Intermittent							Ref	Ref	Ref
Persistent							10·42	8·05–12·78	**<0·0001**

Bold *p*-values denote significance at *p* < 0.05.

Additionally, treatment duration independently predicted improvement, with each additional month associated with greater THI reduction (−1·16 per month, 95% CI −1·38 to −0·94; *p* < 0·0001). Tinnitus characteristics significantly modulated treatment response: persistent tinnitus was associated with poorer outcomes compared to intermittent presentation (10·42, 95% CI 8·05–12·78; *p* < 0·0001), while bilateral tinnitus (−4·04, 95% CI −5·58 to −2·51; *p* < 0·001) and head origin (−4·90, 95% CI −8·65 to −1·15; *p* = 0·010) demonstrated enhanced treatment effects vs unilateral cases. Patient age negatively correlated with improvement (0·10 per year, 95% CI 0·05–0·15; *p* < 0·0001) (Fig. 2C, Table 2), whereas neither sex nor tinnitus pitch significantly influenced therapeutic outcomes.

### Secondary outcomes

**Fig. 3:**
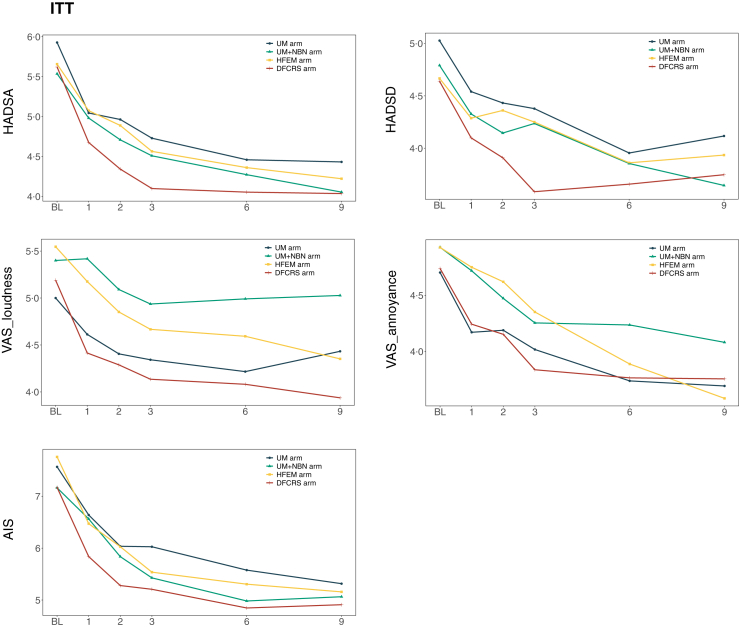
**Secondary outcomes measured by Hospital Anxiety and Depression Scale (HADS), Athens Insomnia Scale-8 (AIS-8), and Visual Analog Scale (VAS).** Anxiety (HADS-A) and Depression (HADS-D) subscales of HADS, the AIS-8, and the VAS for tinnitus loudness and annoyance were measured at baseline and at the 1-month, 2-month, 3-month, 6-month, and 9-month follow-ups in the ITT.

Sound therapy demonstrated significant benefits across all secondary outcome measures. Progressive improvements were observed in tinnitus-related comorbidities over the 9-month intervention period. The AIS-8, HADS-A, HADS-D, and VAS-Loudness and VAS-Annoyance all showed substantial reductions from baseline values (Fig. 3).

Statistical analyses revealed significant group × time interaction effects for all secondary endpoints: AIS-8 (*F*_*(5,1353)*_ = 25·69, *p* < 0·0001), HADS-A *(F*_*(5, 574)*_ = 15·35, *p* < 0·0001), HADS-D (*F*_*(5, 212)*_ = 7·09, *p* < 0·0001), VAS-Loudness (*F*_*(5, 236)*_ = 16·02, *p* < 0·0001), and VAS-Annoyance (*F*_*(5, 323)*_ = 21·12, *p* < 0·0001) (Table S4). The DFCRS intervention consistently demonstrated superior efficacy, with significantly greater reductions in all secondary outcome measures during the initial three-month treatment phase compared to other interventions (Fig. 3). This accelerated improvement trajectory mirrored the primary outcome pattern, confirming DFCRS's enhanced therapeutic profile for alleviating tinnitus-related sleep disturbances, emotional distress, and perceptual symptoms.

Consistent with the GEE model findings for the primary outcome, the DFCRS group demonstrated significantly greater improvements compared with the UM control group across multiple secondary measures, including HADS-A, HADS-D, AIS, and VAS-Loudness (all *p* < 0·05). Patients with bilateral and intermittent tinnitus achieved larger reductions in anxiety, sleep disturbance, and perceived loudness than those with unilateral and persistent tinnitus (all *p* < 0·05) (Table S5).

### Sensitivity analysis

Robustness of the primary findings was evaluated through three sequential GEE models using ITT data (Table 2). The intercept-only null model established baseline variance structure without covariates. Subsequent simplified model incorporating treatment group and time variables demonstrated significantly differential efficacy: HFEM showed inferior treatment response compared to UM reference (2·55, 95% CI 0·55–4·55; *p* = 0·012), while DFCRS exhibited superior efficacy (−4·87, 95% CI −6·80 to −2·94; *p* < 0·0001). Temporal analysis confirmed progressive improvement across all groups (−1·16 per month, 95% CI −1·39 to −0·94; *p* < 0·0001).

The fully adjusted model incorporating demographic and clinical covariates maintained consistent treatment effects: HFEM remained less effective than UM (2·11, 95% CI 0·11–4·11; *p* = 0·039), DFCRS demonstrated sustained superiority (−4·37, 95% CI −6·25 to −2·48; *p* < 0·0001), and temporal improvement persisted unchanged (−1·16, 95% CI −1·38 to −0·94; *p* < 0·0001). Significant covariates predicting treatment response included: advancing age (0·10 per year, 95% CI 0·05–0·15; *p* < 0·0001), low-pitched tinnitus manifestation (vs high-pitched: 2·14, 95% CI 0·39–3·90; *p* = 0·017), bilateral presentation (−4·04, 95% CI −5·58 to −2·51; *p* < 0·0001), head origin (vs unilateral: −4·9, 95% CI −8·65 to −1·15; *p* = 0·010), persistent tinnitus (vs intermittent: 10·42, 95% CI 8·05–12·78; *p* < 0·0001), and tinnitus duration (−0·02 per month, 95% CI −0·04 to −0·00; *p* = 0·031) (Table 2).

According to reports, the minimum clinically important difference (MCID) for THI is 7.^18^ Based on this, we conducted an MCID for THI analysis in this study and found that 92/111 (82·88%) in the UM group, 74/110 (67·27%) in the UM + NBN group, 81/108 (75·0%) in the HFEM group, and 90/111 (81·08%) in the DFCRS group had a Δ THI ≤ −7 (Table S6). Additionally, PP analyses of THI scores corroborated the superiority of DFCRS while showing no significant differences in the HFEM and UM + NBN groups when compared to the UM control group (Figure S3A–C, Table S7). PP analyses of secondary outcomes also confirmed DFCRS as the best compared to other strategies (Table S8). These findings reinforce DFCRS as the most effective intervention across analytical approaches. Notably, the DFCRS group demonstrated the steepest decline in THI and all secondary outcomes during the first 3 months, reflecting an accelerated therapeutic response compared with the other interventions (Figure S3A, D). Importantly, all significant effects observed in the primary analyses were replicated under the PP framework (Table 2 and Table S7), confirming the stability of the model across analytical strategies. This multi-model evaluation underscores that the treatment effect of DFCRS is robust, consistent, and invariant to covariate adjustment, thereby strengthening the validity of the observed clinical benefit.

### Sustained treatment effects

**Fig. 4:**
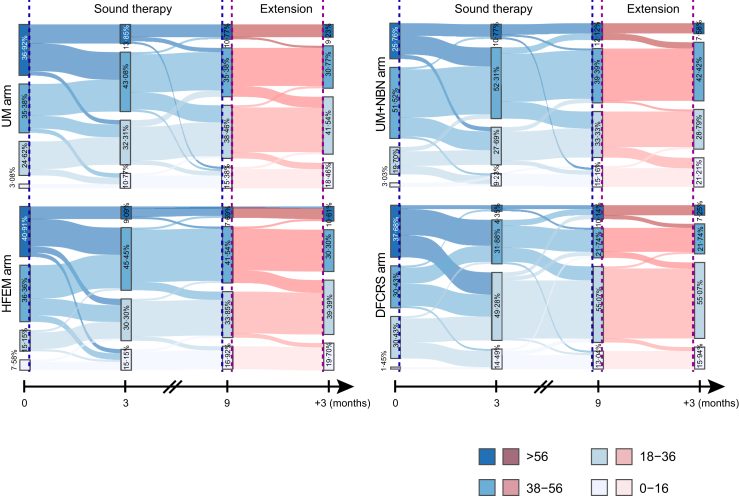
**Sankey diagram illustrating the aftereffects of sound therapy.** Patients completing extended follow-up were included in the analysis. Baseline, 3 months (period of most rapid decline), 9 months (end of sound therapy), and 3 months post-treatment were selected as observation timepoints. Based on THI scores, patients were stratified into four tiers: <16, 18–36, 38–56, and >56 points. The Sankey diagram visualises changes in the proportion of patients across THI tiers at each timepoint and compares patient flow among the four groups: UM, UM + NBN, HFEM, and DFCRS. Arrows indicate cohort progression, with band widths scaled to retention rates (%) at each phase. After treatment discontinuation at 9 months, the majority of patients across all groups maintained their post-treatment status. A minority exhibited further THI score reductions during additional follow-up, indicating sustained therapeutic effects following 9 months of sound therapy.

Longitudinal assessment of tinnitus severity evolution, categorised by THI scores (slight: 0–16; mild: 18–36; moderate: 38–56; severe: ≥58), demonstrated progressive clinical improvement across all intervention groups during the 9-month treatment period. All cohorts exhibited significant migration from higher to lower severity categories, evidenced by substantial reductions in severe cases and corresponding increases in slight/mild classifications relative to baseline. At treatment completion, the combined slight/mild proportions were: UM 35/65 (53·85%), UM + NBN 32/66 (48·48%), HFEM 33/65 (50·77%), and DFCRS 47/69 (68·12%). Post-treatment evaluation at 3-month follow-up revealed sustained clinical benefits, with slight/mild proportions maintained at: UM 39/65 (60·0%), UM + NBN 33/66 (50·0%), HFEM 39/66 (59·09%), and DFCRS 49/69 (71·01%). Taken together, a substantially larger proportion of patients in the DFCRS group transitioned into, and sustained enrollment within, the lowest tinnitus severity categories (slight/mild) by the end of the study compared with patients in the other treatment arms. This persistence of therapeutic gains beyond treatment cessation demonstrates durable treatment effects, particularly pronounced in the DFCRS cohort (Fig. 4). The stability of severity category distributions post-intervention underscores the long-term clinical value of acoustic therapy in tinnitus management.

## Discussion

This MOST multicenter trial represents, to the best of our knowledge, the largest randomised controlled clinical trial to date comparing various sound therapy strategies for patients with chronic subjective tinnitus. All sound therapy approaches demonstrated significant effectiveness in improving both primary and secondary outcomes, with no adverse events reported during the trial. The sustained treatment effects observed 3 months post-intervention further support clinical value of all strategies of sound treatments on tinnitus severity. Notably, DFCRS treatment yielded superior outcomes compared to the other strategies. The results of the ITT analysis of primary outcome were further validated by the findings from the secondary outcome analysis, sensitivity analysis, and PP analysis. The findings mark a significant step forward in understanding and optimising tinnitus management through sound therapy.

In recent years, sound therapy has emerged as one of the most widely researched and utilised non-invasive approaches for managing tinnitus.^19^ Although the basic principles of sound therapy date back to ancient times, contemporary advancements in neuroscience, audiology, and technology have significantly enhanced the efficacy and precision of these treatments.^20^ The evolution of sound therapy reflects a growing understanding of tinnitus as a neurological disorder involving maladaptive neural plasticity, and this has propelled the development of more sophisticated, personalised interventions.

Sound therapy can be broadly categorised into non-customised and customised approaches. In line with prior studies,^7^^,^^21^ our results confirmed the overall effectiveness of acoustic therapy across all four arms, including both non-customised and customised approaches, in reducing tinnitus severity, as measured by the THI score and the secondary outcomes compared to baseline. Non-customised sound therapy includes strategies such as masking therapy^22^ and tinnitus retraining therapy,^23^ which involve the presentation of external sounds that partially or completely cover the perceived tinnitus. Narrowband noise, as used in the UM + NBN arm, is often more effective than broadband noise because it directly targets and competes with the specific auditory perception of tinnitus by centering on the tinnitus frequency. Customised sound therapy, on the other hand, is tailored to an individual's specific tinnitus characteristics, with the aim of promoting cortical reorganisation or altering the pathological synchronisation of neurons, such as tailor-made notched music training (TMNMT),^24^ acoustic coordinated reset neuromodulation (CR),^25^ Heidelberg Neuro-Music Therapy,^26^ and our DFCRS strategy. This durability aligns with neuroplasticity principles, suggesting long-term reorganisation of auditory processing networks.

While all sound therapy approaches demonstrated some clinical benefit, the consistently superior performance of DFCRS is likely attributed to its individualised and neurophysiological informed design. Tinnitus has been linked to elevated spontaneous firing rates, maladaptive cortical reorganisation, and abnormal neural synchrony within auditory and cognitive networks.^27^ The DFCRS strategy directly targets these aberrant mechanisms by employing tinnitus pitch-centered modulation designed to strengthen lateral inhibition and promote neural desynchronisation. By delivering stimulus patterns that flank or precisely match the tinnitus frequency, DFCRS might recruit adjacent auditory neurons that exert inhibitory influence on hyperactive circuits, thereby suppressing overrepresented tinnitus-related activity. Concurrently, the burst-like delivery format appears to disrupt abnormally synchronised firing patterns, facilitating a rebalancing of cortical excitability. Over time, these combined effects may drive adaptive plasticity within auditory–cognitive networks and lead to more durable reductions in tinnitus perception.

Several recent studies have provided compelling clinical evidence supporting the effectiveness of contemporary sound therapy approaches. For instance, Reavis et al.^28^ investigated the use of amplitude-modulated tones in the high-frequency range to match the tinnitus pitch, and they found that modulated sounds, particularly those with low-rate amplitude modulation, could significantly reduce the perceived loudness of tinnitus. Similarly, Okamoto et al.^24^ demonstrated the efficacy of TMNMT, which removes the tinnitus frequency from the music. Moreover, CR, which uses carefully timed sequences of sound pulses to disrupt pathological neural synchrony, remains a promising area of research. Studies such as Tass et al.^29^ have shown that CR therapy can reduce tinnitus severity by promoting the long-term desynchronisation of abnormally synchronised neurons. In the present study, we employed a novel strategy in the DFCRS arm, distinct from TMNMT and CR, by using a flexible, frequency-customised sound profile that dynamically amplified specified bands around the tinnitus pitch. The DFCRS approach demonstrated greater efficacy in reducing tinnitus severity compared to the HFEM strategy, which employs high-frequency enhancement irrespective of the tinnitus pitch. Therefore, our findings extend prior research on customised therapies by demonstrating superior efficacy of dynamic frequency targeting compared to static modulation approaches.

We also noticed that several factors emerged as critical in determining treatment outcomes, reinforcing the importance of a personalised approach to tinnitus management. Both treatment duration and tinnitus characteristics emerged as significant predictors of treatment success. Patients with intermittent tinnitus experienced better outcomes compared to those with persistent tinnitus, likely due to the less entrenched neural pathways in cases of intermittent symptoms. Interestingly, age and tinnitus location also played a role, with younger patients and those with bilateral tinnitus showing slightly better responses to treatment. However, tinnitus frequency did not significantly impact treatment efficacy, suggesting that while personalised sound therapy is effective, its success may not be solely contingent on the specific pitch of the tinnitus.^21^ A longer tinnitus duration was statistically associated with better treatment outcomes; however, the effect size (0·02) was negligible, indicating that tinnitus duration exerted minimal clinical influence on treatment efficacy.

Additionally, the integration of digital platforms, such as the Fudan Tinnitus-Relief System (FTRS),^10^ has revolutionised access to customised sound therapy. These applications offer frequency-specific stimuli, real-time feedback, and adaptive treatments, enabling patients to engage in therapy conveniently and effectively.

Several limitations of this study warrant consideration. Most notably, the absence of a no-treatment control arm limits the extent to which the therapeutic benefits of sound therapy can be unequivocally distinguished from placebo effects or the possibility of natural symptom fluctuations over time. In clinical trials of tinnitus sound therapy, waitlist controls are often employed as comparators. Prior evidence suggests that while short waiting periods may yield modest reductions in tinnitus-related questionnaire scores,^30^ extended waiting generally results in significantly poorer outcomes relative to active treatment groups.^31^ Another limitation of this study is the absence of objective adherence monitoring tools (e.g., daily listening logs or device-based tracking) to quantify individual listening duration. This limitation constrains our ability to directly assess the relationship between treatment adherence and therapeutic outcomes. Future research could address this by incorporating digital or device-based tracking systems to more accurately correlate listening duration with treatment efficacy.

In this exploratory trial, we chose not to formally adjust the significance threshold (α) for multiplicity. The decision is consistent with the hypothesis-generating nature of the study but also increases the risk of Type I errors (false-positive findings). Accordingly, the statistical results should be interpreted with caution, as they are best viewed as preliminary indications of potential patterns or associations. These findings highlight hypotheses to be tested in subsequent confirmatory studies that will employ stricter control conditions and appropriate statistical safeguards. Despite these limitations, the present findings support DFCRS as a promising and superior therapeutic strategy. The integration of DFCRS with digital platforms offers the potential for scalable and individualised sound therapy delivery, thereby accommodating the high prevalence and heterogeneous presentation of tinnitus in routine clinical practice. To enhance the external validity of these findings, future studies should aim to replicate and extend these results across more diverse geographic regions, ethnic populations, and healthcare settings. A key limitation of the present study is the relatively short duration of follow-up (3 months). Longer-term follow-up is essential to determine whether the therapeutic effects of sound therapy are sustained over time and to what extent they are associated with auditory central plasticity. To address this, we will continue to monitor patients beyond the initial 3-month period, and future studies are planned with longer structured follow-up intervals (e.g., 6 months or more) to more comprehensively evaluate persistence of therapeutic benefit as well as potential relapse of tinnitus in relation to sound therapy. Such efforts would not only strengthen generalisability but also inform optimal implementation strategies in real-world contexts.

In conclusion, sound therapy significantly reduced tinnitus severity over 9 months, with benefits persisting 3 months posttreatment. DFCRS demonstrates significant advantages over conventional sound therapies in reducing tinnitus severity and associated comorbidities, with durable treatment effects. Younger patients, longer treatment duration, and bilateral or head-origin tinnitus, as well as those with intermittent tinnitus, derived notable benefit from acoustic intervention. These findings establish frequency-customised neuromodulation as a promising cornerstone of evidence-based tinnitus management.

## Contributors

All authors made substantial intellectual contributions to this study. DMT, SS and HWL conceived the trial. YFW, JNZ, and XSH were responsible for the diagnosis, clinical screening, and participant recruitment. DTG generated the random allocation sequence and intervention assignment. JMG, GYL, LZ, AQD, PFG, and YH collected the data. DMT, SS, and DTG contributed to the clinical trial registration, obtained ethical approval for the study, and performed the statistical analysis. DMT, DTG, and SS drafted the manuscript. HWL and SS reviewed and edited the manuscript. DMT and DTG have access to and verify the underlying study data. HWL was the principal investigator of the trial and contributed to the conception of the study. All authors participated in revising the manuscript and approved the final version.

## Data sharing statement

The datasets generated and analysed during the current study will be made available upon reasonable request after the manuscript is accepted for publication. Researchers can contact the corresponding author at hwli@shmu.edu.cn to request access to the data. Data will be shared in accordance with institutional and ethical guidelines, and any necessary approvals will be required prior to sharing.

## Declaration of interests

All authors declare no competing interests.

## Appendix A Supplementary data

Supplementary Files

UM+NBN-4000Hz-1 3octave

UM.mp3

DFCRS-4000hz-10.00dB

HFEM-6000-9000-10.00dB
