# Supplementary material for: Efficacy and safety of a modified sound therapy for patients with subjective tinnitus (MOST): a multicentre, double-blind, randomised controlled trial
Source: eClinicalMedicine. 2025 Dec 8;90:103671. doi: 10.1016/j.eclinm.2025.103671 (PMC12766481; doi:10.1016/j.eclinm.2025.103671)
Supplement: Supplementary Files [file mmc1.pdf]

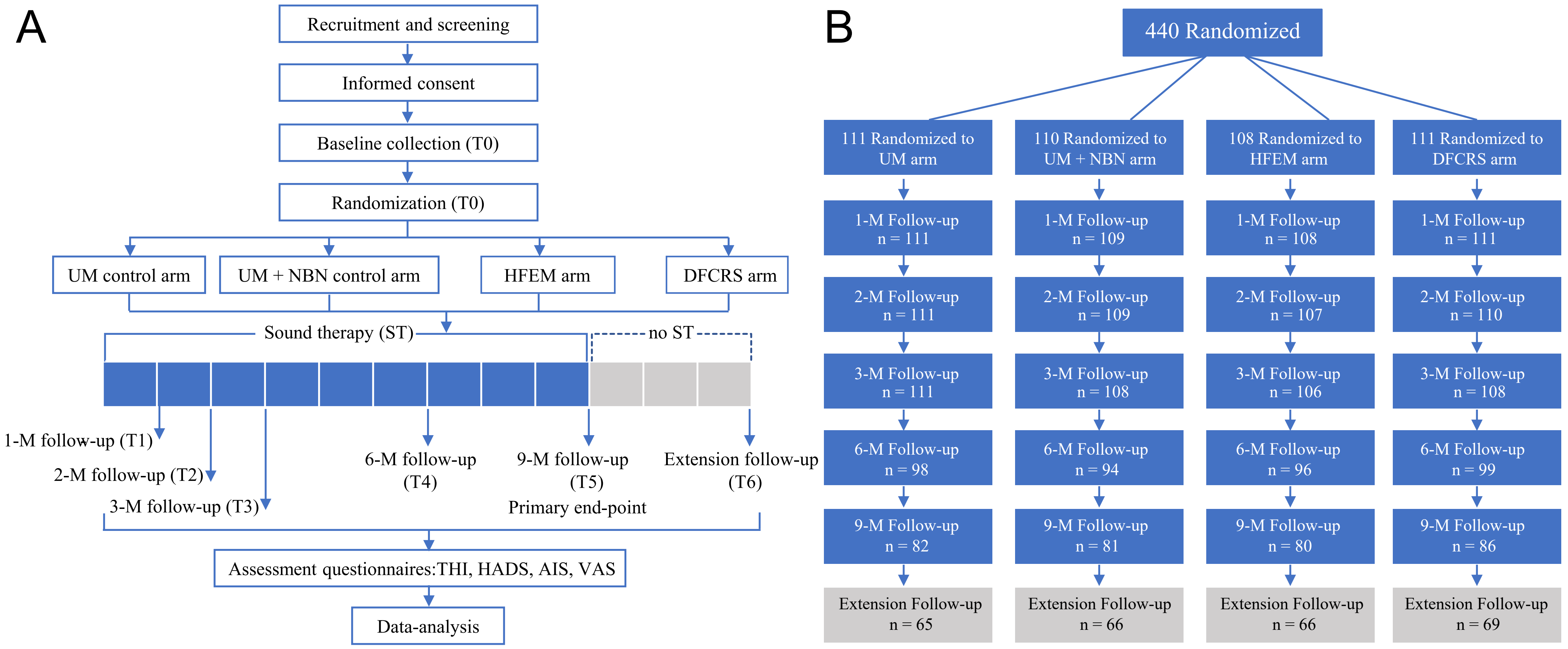


Supplementary Figure S1

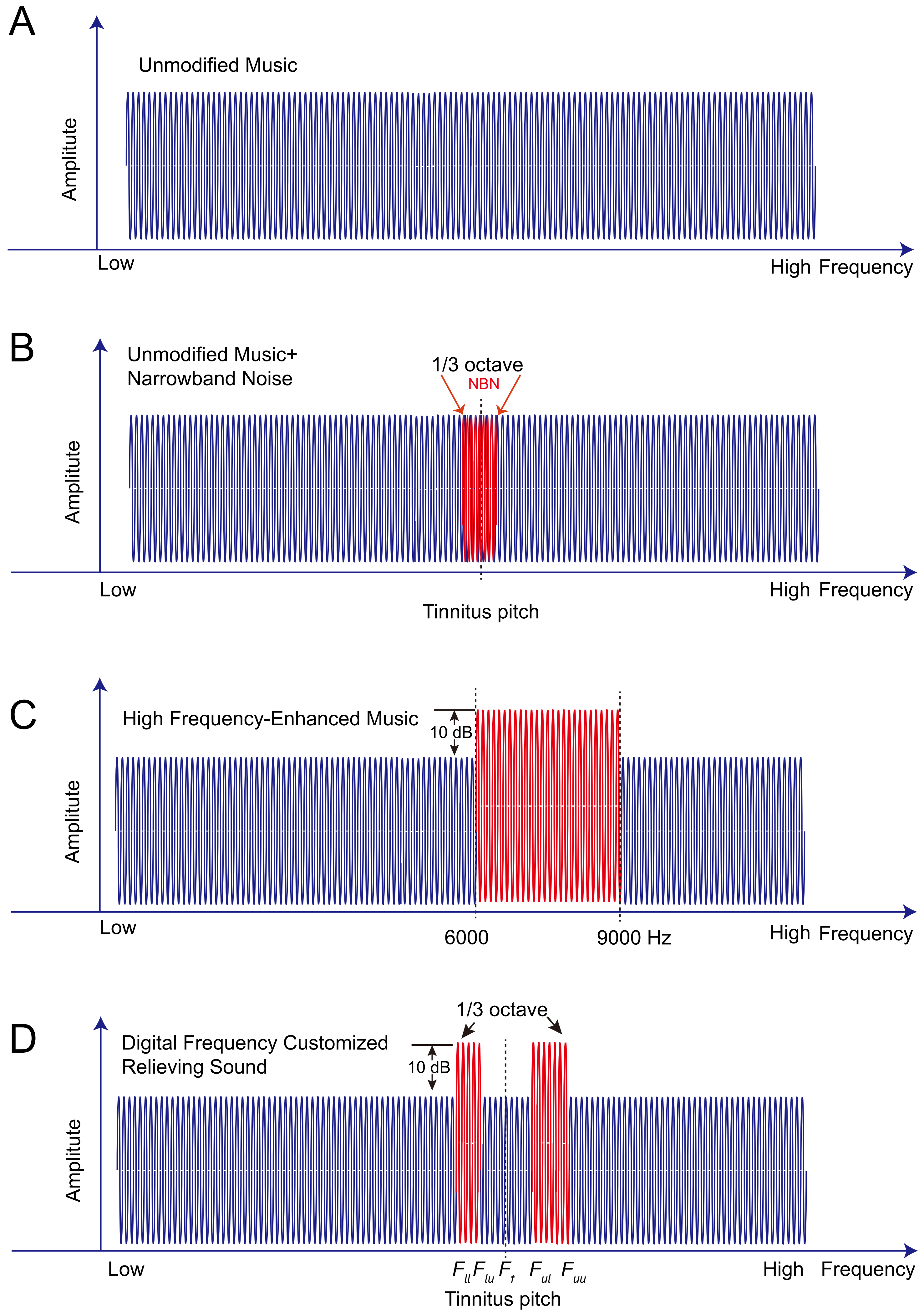


Supplementary Figure S2

**A**

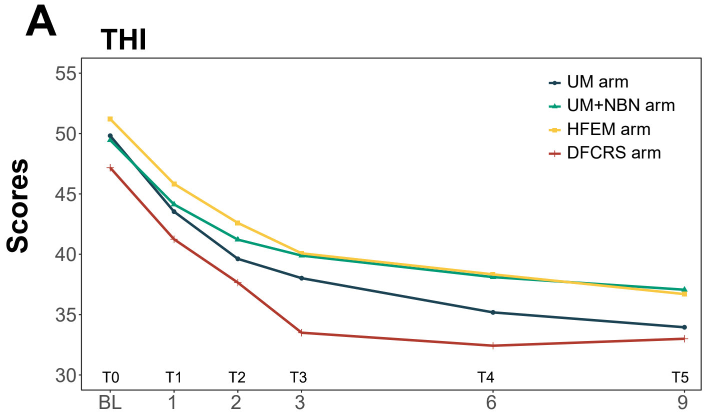


**B**

| | T0 | T1 | T2 | T3 | T4 | T5 |
|---|---|---|---|---|---|---|
| UM | 49·82±18·37 (46·36,53·28) | 43·53±17·93 (40·16,46·90) | 39·62±16·95 (36·43,42·81) | 38·02±17·25 (34·77,41·26) | 35·18±17·20 (31·74,38·63) | 33·95±17·83 (30·03,37·87) |
| UM+NBN | 49·45±18·06 (46·04,52·87) | 44·13±17·27 (40·85,47·41) | 41·21±16·79 (38·02,44·40) | 39·89±16·76 (36·69,43·09) | 38·11±16·56 (34·71,41·50) | 37·06±17·43 (33·21,40·92) |
| HFEM | 51·20±19·93 (47·40,55·01) | 45·83±17·83 (42·43,49·24) | 42·60±17·90 (39·17,46·03) | 40·08±18·04 (36·60,43·55) | 38·33±19·21 (34·44,42·23) | 36·70±18·39 (32·61,40·79) |
| DFCRS | 47·17±17·66 (46·85,53·79) | 41·24±17·52 (37·95,44·54) | 37·66±16·32 (34·57,40·74) | 33·5±15·75 (30·50,36·50) | 32·42±16·58 (29·12,35·73) | 33·00±17·06 (29·34,36·66) |
| *p* | 0·17 | **0·0031** | **< 0·0001** | **< 0·0001** | **< 0·0001** | **0·0001** |

Mean ± SD (95%CI)

**C**

| Variable | N | Estimate | *p* |
|---|---|---|---|
| **Group** | | | |
| UM arm | 578 | Reference | |
| UM+NBN arm | 565 | 0·15 (-1·84, 2·14) | 0·88 |
| HFEM arm | 558 | 1·91 (-0·17, 3·98) | 0·072 |
| DFCRS arm | 578 | -4·37 (-6·32, -2·43) | **<0·0001** |
| **Time** | 2279 | -1·19 (-1·43, -0·95) | **<0·0001** |
| **Gender** | | | |
| Male | 1162 | Reference | |
| Female | 1117 | 0·80 (-0·66, 2·26) | 0·28 |
| **Age** | 2279 | 0·10 (0·06, 0·15) | **<0·0001** |
| **Duration** | 2279 | -0·02 (-0·04, -0·00) | **0·034** |
| **Location** | | | |
| Unilateral | 1439 | Reference | |
| Bilateral | 813 | -3·71 (-5·31, -2·10) | **<0·0001** |
| In head | 27 | -4·84 (-8·80, -0·89) | **0·016** |
| **Tone** | | | |
| High-pitched | 1733 | Reference | |
| Low-pitched | 516 | 1·79 (-0·04, 3·61) | 0·055 |
| Noise-like | 30 | 2·85 (-5·64, 11·35) | 0·51 |
| **Type** | | | |
| intermittent | 217 | Reference | |
| continued | 2062 | 10·01 (7·55, 12·46) | **<0·0001** |

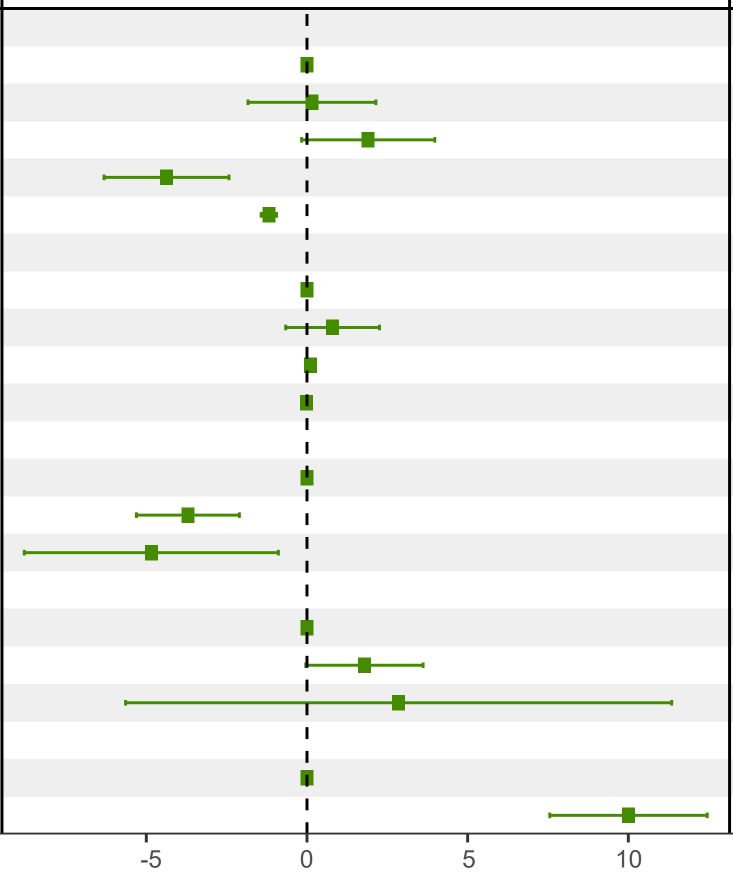


**D**

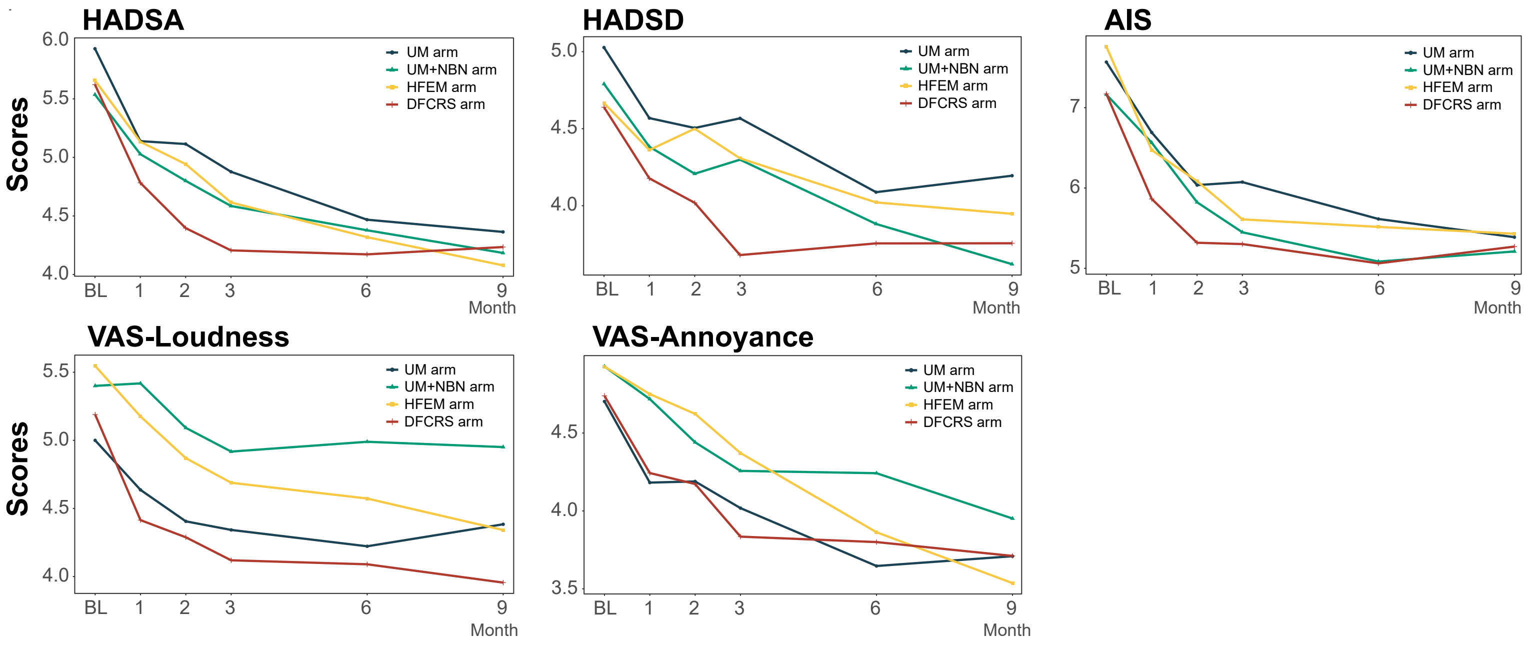

**Supplementary figure legends.**

**Figure S1. Recruitment and study procedures.**

(A) Overview of the study design, including screening, recruitment, randomization, intervention procedures, follow-up schedule, and data analysis framework.

(B) Participant distribution across treatment groups at baseline and at each follow-up time point, illustrating retention and dropout rates throughout the study.

**Figure S2. The illustration of 4 different acoustic strategies.**

(A) UM, unmodified music without any modulation.

(B) UM+NBN, unmodified music combined with narrowband noise; a mixture of unmodulated pure music and 1/3 octave narrowband noise centered on the tinnitus pitch.

(C) HFEM, high-frequency enhanced music; an intensification modulation of 10 dB is performed on a pure piece of music in the 6000-9000Hz range.

(D) DFCRS, digital frequency customized relieving sound; the intensity of the music is dynamically enhanced by 10 dB within the two 1/3 octave frequency bands below ($F_{ll}$ to $F_{lu}$) and above ($F_{ul}$ to $F_{uu}$) tinnitus pitch. The relationship of $F_{ll}$, $F_{lu}$, $F_{ul}$, and $F_{uu}$ are determined by special algorithms in study protocol, $F_u=2F_l$, $F_u \times F_l=F_t^2$. $F_t$, tinnitus pitch; $F_{uu}$, the upper endpoint of 1/3 octave centered at $F_u$; $F_{ul}$, the lower endpoint of 1/3 octave centered at $F_u$; $F_{ll}$, the lower endpoint of 1/3 octave centered at $F_l$; and $F_{lu}$, the upper endpoint of 1/3 octave centered at $F_l$.

**Figure S3. Per-protocol analysis of Tinnitus Handicap Inventory (THI) scores and other secondary outcomes.**

(A-B) THI scores at baseline and at the 1-month, 2-month, 3-month, 6-month, and 9-month follow-ups in the per-protocol (PP) analyses.

(C) The Generalized Estimating Equation (GEE) model based on PP analyses.

(D) Secondary outcomes measured by Hospital Anxiety and Depression Scale (HADS), Athens Insomnia Scale-8 (AIS-8), and Visual Analog Scale (VAS) at baseline and at the 1-month, 2-month, 3-month, 6-month, and 9-month follow-ups in the PP analyses.

**Supplementary tables.**

**Table S1. Baseline Characteristics by research unit.**

| | level | | Overall (N = 440) | EENT Hospital (N = 199) | YY Hospital (N = 180) | ZS Hospital (N = 61) | *p* value |
|---|---|---|---|---|---|---|---|
| Group | UM | | 111 (25·23) | 50 (25·13) | 45 (25·00) | 16 (26·23) | 1·00 |
| | UM+NBN | | 110 (25·00) | 50 (25·13) | 45 (25·00) | 15 (24·59) | |
| | HFEM | | 108 (24·54) | 49 (24·61) | 45 (25·00) | 14 (22·95) | |
| | DFCRS | | 111 (25·23) | 50 (25·13) | 45 (25·00) | 16 (26·23) | |
| Sex | Male | | 222 (50·45) | 103 (51·76) | 91 (50·56) | 28 (45·90) | 0·73 |
| | Female | | 218 (49·55) | 96 (48·24) | 89 (49·44) | 33 (54·10) | |
| Age | | | 45·00 [35·00, 56·00] | 45·00 [37·00, 55·00] | 44·00 [34·00, 57·00] | 45·00 [36·00, 54·00] | 1·00 |
| Tinnitus duration | | | 13·00 [7·00, 36·00] | 13·00 [6·00, 60·00] | 15·00 [8·00, 28·00] | 12·00 [6·00, 36·00] | 0·20 |
| Tinnitus location | Unilateral | | 278 (63·18) | 98 (49·25) | 150 (83·33) | 30 (49·18) | **<0·0001** |
| | Bilateral | | 151 (34·32) | 99 (49·75) | 21 (11·67) | 31 (50·82) | |
| | In head | | 11 (2·50) | 2 (1·00) | 9 (5·00) | 0 (0·00) | |
| Tinnitus tone | High pitch | | 332 (75·45) | 147 (73·87) | 140 (77·78) | 45 (73·77) | 0·088 |
| | Low pitch | | 102 (23·18) | 49 (24·62) | 40 (22·22) | 13 (21·31) | |
| | Noise-like | | 6 (1·37) | 3 (1·51) | 0 (0·00) | 3 (4·92) | |
| Tinnitus type | Intermittent | | 59 (13·41) | 17 (8·54) | 28 (15·56) | 14 (22·95) | **0·008** |
| | Persistent | | 381 (86·59) | 182 (91·46) | 152 (84·44) | 47 (77·05) | |
| Average PTA* (AC) | Right | | 21·25 [15·00, 35·00] | 21·25 [16·25, 33·75] | 24·38 [13·75, 37·50] | 20·00 [15·00, 30·00] | 0·28 |
| | Left | | 22·50 [14·38, 33·75] | 20·62 [15·00, 33·75] | 25·00 [13·75, 38·75] | 21·25 [15·00, 30·00] | 0·55 |
| THI$_{BL}$ | | | 50·00 [36·00, 62·00] | 50·00 [36·00, 64·00] | 52·00 [42·00, 62·00] | 42·00 [26·00, 68·00] | 0·14 |
| HADS-$_{BL}$ | Anxiety | | 6·00 [3·00, 8·00] | 5·00 [3·00, 8·00] | 6·00 [5·00, 7·00] | 4·00 [3·00, 8·00] | 0·37 |
| | Depression | | 5·00 [3·00, 6·00] | 4·00 [2·00, 7·00] | 5·00 [4·00, 6·00] | 4·00 [2·00, 6·00] | 0·066 |
| AIS$_{BL}$ | | | 7·00 [5·00, 9·00] | 7·00 [4·00, 10·50] | 7·00 [6·00, 8·00] | 7·00 [4·00, 10·00] | 0·40 |
| VAS$_{BL}$- | Loudness | | 5·00 [4·00, 6·00] | 5·00 [4·00, 7·00] | 5·00 [5·00, 6·00] | 4·00 [3·00, 5·00] | **<0·0001** |
| | Annoyance | | 5·00 [3·75, 6·00] | 5·00 [3·00, 6·00] | 5·00 [4·00, 6·00] | 4·00 [3·00, 5·00] | **0·038** |
| Tinnitus PM$_{BL}$- | Left | Low | 66 (23·00) | 33 (23·57) | 28 (25·00) | 4 (11·76) | **0·042** |
| | | Mid | 68 (23·69) | 39 (27·86) | 17 (15·18) | 12 (35·29) | |
| | | High | 153 (53·31) | 68 (48·57) | 67 (59·82) | 18 (52·95) | |
| | Right | Low | 55 (21·32) | 35 (23·03) | 18 (20·93) | 2 (10·00) | 0·38 |
| | | Mid | 68 (26·36) | 44 (28·95) | 18 (20·93) | 6 (30·00) | |
| | | High | 135 (52·32) | 73 (48·02) | 50 (58·14) | 12 (60·00) | |
| Tinnitus L$_{BL}$-(SL) | Left | | 10·00 [7·00, 14·00] | 9·00 [5·00, 15·00] | 11·00 [9·00, 13·00] | 5·00 [4·00, 8·75] | **<0·0001** |
| | Right | | 10·00 [6·00, 13·00] | 9·00 [3·00, 17·00] | 10·00 [9·00, 12·00] | 6·00 [3·75, 10·00] | **0·008** |

Data are median [IQR] or n (%). * the average pure tone hearing thresholds of 0.5, 1, 2, and 4 kHz. Low*

means < 1000 Hz; Mid* means 1000 Hz (inclusive) ~ 4000 Hz (exclusive); High* means 4000 Hz (inclusive) ~ 8000 Hz (inclusive).
Abbreviation: AC, air conduction; AIS, Athens Insomnia Scale; BL, baseline; HADS, Hospital Anxiety and Depression Scale; HL, hearing level; PM, pitch match; PTA, pure tone audiometry; SL, sensation level; THI, Tinnitus Handicap Inventory; VAS, Visual Analogue Scale.

## Table S2. The overall treatment effect of sound therapy over a 9-month follow-up.

| | **Baseline** | **1-month follow-up** | **2-month follow-up** | **3-month follow-up** | **6-month follow-up** | **9-month follow-up** | ***p*** |
|---|---|---|---|---|---|---|---|
| **n** | 440 | 422 | 414 | 410 | 362 | 304 | |
| **THI** | 50.00 [36.00, 62.00] | 46.00 [32.00, 56.00] | 42.00 [32.00, 52.00] | 39.00 [28.00, 49.50] | 36.00 [26.00, 48.00] | 35.00 [24.00, 48.00] | <0.0001 |
| **HADS-A** | 6.00 [3.00, 8.00] | 5.00 [3.00, 6.75] | 5.00 [3.00, 6.00] | 4.00 [3.00, 6.00] | 4.00 [3.00, 6.00] | 4.00 [2.00, 6.00] | <0.0001 |
| **HADS-D** | 5.00 [3.00, 6.00] | 4.00 [3.00, 6.00] | 4.00 [3.00, 6.00] | 4.00 [3.00, 5.00] | 4.00 [2.00, 5.00] | 4.00 [2.00, 5.00] | <0.0001 |
| **AIS** | 7.00 [5.00, 9.00] | 6.00 [4.00, 8.00] | 6.00 [4.00, 7.00] | 5.00 [4.00, 7.00] | 5.00 [4.00, 7.00] | 5.00 [4.00, 7.00] | <0.0001 |
| **VAS-Loudness** | 5.00 [4.00, 6.00] | 5.00 [4.00, 6.00] | 5.00 [4.00, 6.00] | 5.00 [3.00, 6.00] | 4.00 [4.00, 6.00] | 4.00 [3.00, 6.00] | <0.0001 |
| **VAS-Annoyance** | 5.00 [3.75, 6.00] | 4.00 [3.00, 5.00] | 4.00 [3.00, 5.00] | 4.00 [3.00, 5.00] | 4.00 [3.00, 5.00] | 4.00 [3.00, 5.00] | <0.0001 |

Data are median [IQR], mean(SD) or n (%).
Abbreviation: AIS, Athens Insomnia Scale; HADS, Hospital Anxiety and Depression Scale; THI, Tinnitus Handicap Inventory; VAS, Visual Analogue Scale.

**Table S3. The scores of THI (primary outcome) at baseline, 1-, 2-, 3-, 6-, and 9-month follow-up among different groups.**

| Group | Follow-up (months) | n | Mean (±SD) | Mean (95% CI) | Cohen *d* (95%CI) | *F-Value* | *P-Value* |
|---|---|---|---|---|---|---|---|
| UM | BL | 111 | 49·82±18·37 | 49·82(46·36,53·28) | ref | $F_{(5, 618)}$ = 11·45 | **<0·0001** |
| | 1 | 111 | 43·53±17·93 | 43·53(40·16,46·9) | 0·32(0·18,0·45) | | |
| | 2 | 111 | 39·62±16·95 | 39·62(36·43,42·81) | 0·51(0·38,0·65) | | |
| | 3 | 111 | 38·02±17·25 | 38·02(34·77,41·26) | 0·65(0·51,0·78) | | |
| | 6 | 98 | 35·18±17·2 | 35·18(31·74,38·63) | 0·74(0·6,0·89) | | |
| | 9 | 82 | 33·95±17·83 | 33·95(30·03,37·87) | 0·79(0·64,0·93) | | |
| UM+NBN | BL | 110 | 49·45±18·06 | 49·45(46·04,52·87) | ref | $F_{(5, 605)}$ = 7·17 | **<0·0001** |
| | 1 | 109 | 44·13±17·27 | 44·13(40·85,47·41) | 0·32(0·18,0·45) | | |
| | 2 | 109 | 41·21±16·79 | 41·21(38·02,44·4) | 0·51(0·38,0·65) | | |
| | 3 | 108 | 39·89±16·76 | 39·89(36·69,43·09) | 0·65(0·51,0·78) | | |
| | 6 | 94 | 38·11±16·56 | 38·11(34·71,41·5) | 0·74(0·6,0·89) | | |
| | 9 | 81 | 37·06±17·43 | 37·06(33·21,40·92) | 0·79(0·64,0·93) | | |
| HFEM | BL | 108 | 51·2±19·93 | 51·2(47·4,55·01) | ref | $F_{(5, 599)}$ = 8·3 | **<0·0001** |
| | 1 | 108 | 45·83±17·83 | 45·83(42·43,49·24) | 0·32(0·18,0·45) | | |
| | 2 | 107 | 42·6±17·9 | 42·6(39·17,46·03) | 0·51(0·38,0·65) | | |
| | 3 | 106 | 40·08±18·04 | 40·08(36·6,43·55) | 0·65(0·51,0·78) | | |
| | 6 | 96 | 38·33±19·21 | 38·33(34·44,42·23) | 0·74(0·6,0·89) | | |
| | 9 | 80 | 36·7±18·39 | 36·7(32·61,40·79) | 0·79(0·64,0·93) | | |
| DFCRS | BL | 111 | 47·17±17·66 | 47·17(43·85,50·49) | ref | $F_{(5,619)}$ = 12·65 | **<0·0001** |
| | 1 | 111 | 41·24±17·52 | 41·24(37·95,44·54) | 0·32(0·18,0·45) | | |
| | 2 | 110 | 37·66±16·32 | 37·66(34·57,40·74) | 0·51(0·38,0·65) | | |
| | 3 | 108 | 33·5±15·75 | 33·5(30·5,36·5) | 0·65(0·51,0·78) | | |
| | 6 | 99 | 32·42±16·58 | 32·42(29·12,35·73) | 0·74(0·6,0·89) | | |
| | 9 | 86 | 33±17·06 | 33(29·34,36·66) | 0·79(0·64,0·93) | | |

F values are from the overall contrast for group × time (4×7) interaction. Cohen *d* values are within-group time effects for baseline to each post assessment using the model mean difference divided by the whole-sample preassessment SD.

Abbreviation: THI, Tinnitus Handicap Inventory.

## Table S4. Secondary outcomes of tinnitus treatment.

| Variable | UM | | | UM+NBN | | | HFEM | | | DFCRS | | | F-Value | *P*-Value |
|---|---|---|---|---|---|---|---|---|---|---|---|---|---|---|
| | n | Mean (±SD) | Cohen *d* (95%CI) | n | Mean (±SD) | Cohen *d* (95%CI) | n | Mean (±SD) | Cohen d (95%CI) | n | Mean (±SD) | Cohen *d* (95%CI) | | |
| **AIS** | | | | | | | | | | | | | | |
| BL | 111 | 7·57±4·05 | ref | 110 | 7·16±3·36 | ref | 108 | 7·76±3·86 | ref | 111 | 7·17±3·42 | ref | | |
| 1-mth | 110 | 6·69±3·58 | 0·29(0·15,0·42) | 110 | 6·56±3·51 | 0·29(0·15,0·42) | 108 | 6·47±3·38 | 0·29(0·15,0·42) | 110 | 5·86±3·09 | 0·29(0·15,0·42) | | |
| 2-mth | 111 | 6·04±3·28 | 0·47(0·34,0·61) | 107 | 5·82±2·79 | 0·47(0·34,0·61) | 106 | 6·08±3·23 | 0·47(0·34,0·61) | 110 | 5·32±2·89 | 0·47(0·34,0·61) | $F_{(5,1353)}$ = 25·69 | **<0·0001** |
| 3-mth | 109 | 6·07±3·6 | 0·53(0·39,0·66) | 107 | 5·45±2·75 | 0·53(0·39,0·66) | 105 | 5·61±3·11 | 0·53(0·39,0·66) | 106 | 5·3±2·97 | 0·53(0·39,0·66) | | |
| 6-mth | 96 | 5·62±3·27 | 0·61(0·47,0·75) | 95 | 5·08±2·76 | 0·61(0·47,0·75) | 95 | 5·52±3·38 | 0·61(0·47,0·75) | 97 | 5·06±2·88 | 0·61(0·47,0·75) | | |
| 9-mth | 80 | 5·39±3·09 | 0·61(0·47,0·76) | 81 | 5·21±2·62 | 0·61(0·47,0·76) | 79 | 5·43±2·97 | 0·61(0·47,0·76) | 85 | 5·27±3·23 | 0·61(0·47,0·76) | | |
| **HADS-A** | | | | | | | | | | | | | | |
| BL | 111 | 5·93±3·19 | ref | 110 | 5·54±2·81 | ref | 108 | 5·66±3·08 | ref | 111 | 5·62±3·25 | ref | | |
| 1-mth | 108 | 5·14±2·81 | 0·23(0·09,0·36) | 108 | 5·03±2·88 | 0·23(0·09,0·36) | 105 | 5·13±2·56 | 0·23(0·09,0·36) | 106 | 4·78±2·81 | 0·23(0·09,0·36) | | |
| 2-mth | 105 | 5·11±2·68 | 0·31(0·17,0·44) | 105 | 4·8±2·57 | 0·31(0·17,0·44) | 105 | 4·94±2·62 | 0·31(0·17,0·44) | 106 | 4·4±2·49 | 0·31(0·17,0·44) | $F_{(5,574)}$ = 15·35 | **<0·0001** |
| 3-mth | 106 | 4·88±2·7 | 0·39(0·25,0·52) | 106 | 4·58±2·6 | 0·39(0·25,0·52) | 104 | 4·62±2·64 | 0·39(0·25,0·52) | 102 | 4·21±2·53 | 0·39(0·25,0·52) | | |
| 6-mth | 94 | 4·47±2·77 | 0·47(0·33,0·61) | 90 | 4·38±2·56 | 0·47(0·33,0·61) | 94 | 4·32±2·75 | 0·47(0·33,0·61) | 93 | 4·17±2·62 | 0·47(0·33,0·61) | | |
| 9-mth | 77 | 4·36±2·94 | 0·51(0·36,0·66) | 76 | 4·18±2·33 | 0·51(0·36,0·66) | 77 | 4·08±2·28 | 0·51(0·36,0·66) | 81 | 4·24±2·76 | 0·51(0·36,0·66) | | |
| **HADS-D** | | | | | | | | | | | | | | |
| BL | 111 | 5·03±2·75 | ref | 110 | 4·79±2·72 | ref | 108 | 4·67±2·78 | ref | 111 | 4·64±2·78 | ref | | |
| 1-mth | 109 | 4·57±2·53 | 0·16(0·02,0·29) | 107 | 4·38±2·44 | 0·16(0·02,0·29) | 105 | 4·36±2·28 | 0·16(0·02,0·29) | 107 | 4·18±2·49 | 0·16(0·02,0·29) | | |
| 2-mth | 107 | 4·5±2·45 | 0·18(0·05,0·32) | 106 | 4·21±2·26 | 0·18(0·05,0·32) | 104 | 4·5±2·55 | 0·18(0·05,0·32) | 105 | 4·02±2·43 | 0·18(0·05,0·32) | $F_{(5,212)}$ = 7·09 | **<0·0001** |
| 3-mth | 104 | 4·57±2·42 | 0·22(0·09,0·35) | 107 | 4·3±2·33 | 0·22(0·09,0·35) | 104 | 4·31±2·36 | 0·22(0·09,0·35) | 103 | 3·68±2·38 | 0·22(0·09,0·35) | | |
| 6-mth | 91 | 4·09±2·24 | 0·33(0·19,0·47) | 93 | 3·88±2·13 | 0·33(0·19,0·47) | 93 | 4·02±2·38 | 0·33(0·19,0·47) | 94 | 3·76±2·27 | 0·33(0·19,0·47) | | |
| 9-mth | 77 | 4·2±2·53 | 0·35(0·2,0·5) | 79 | 3·62±2·23 | 0·35(0·2,0·5) | 76 | 3·95±2·1 | 0·35(0·2,0·5) | 82 | 3·76±2·45 | 0·35(0·2,0·5) | | |

| | | | | | | | | | | | | | | |
|---|---|---|---|---|---|---|---|---|---|---|---|---|---|---|
| **VAS-loudness** | | | | | | | | | | | | | | |
| BL | 111 | 5±1·76 | ref | 110 | 5·4±1·84 | ref | 108 | 5·55±1·88 | ref | 111 | 5·19±1·62 | ref | | |
| 1-mth | 110 | 4·64±1·53 | 0·21(0·08,0·34) | 110 | 5·42±1·79 | 0·21(0·08, 0·34) | 108 | 5·18±1·86 | 0·21(0·08,0·34) | 111 | 4·41±1·56 | 0·21(0·08, 0·34) | | |
| 2-mth | 111 | 4·4±1·55 | 0·36(0·23,0·49) | 109 | 5·09±1·6 | 0·36(0·23, 0·49) | 107 | 4·87±1·78 | 0·36(0·23,0·49) | 111 | 4·29±1·61 | 0·36(0·23, 0·49) | $F_{(5,236)}$ = 16·02 | **<0·0001** |
| 3-mth | 111 | 4·34±1·55 | 0·44(0·31,0·57) | 109 | 4·92±1·79 | 0·44(0·31, 0·57) | 106 | 4·69±1·7 | 0·44(0·31,0·57) | 109 | 4·12±1·65 | 0·44(0·31, 0·57) | | |
| 6-mth | 99 | 4·22±1·61 | 0·47(0·33,0·61) | 95 | 4·99±1·61 | 0·47(0·33, 0·61) | 96 | 4·57±1·69 | 0·47(0·33,0·61) | 100 | 4·09±1·69 | 0·47(0·33, 0·61) | | |
| 9-mth | 86 | 4·38±1·56 | 0·5(0·36, 0·65) | 81 | 4·95±1·77 | 0·5(0·36,0·65) | 82 | 4·34±1·58 | 0·5(0·36, 0·65) | 90 | 3·96±1·9 | 0·5(0·36,0·65) | | |
| **VAS-annoyance** | | | | | | | | | | | | | | |
| BL | 111 | 4·7±1·67 | ref | 110 | 4·93±1·85 | ref | 108 | 4·93±1·91 | ref | 111 | 4·74±1·79 | ref | | |
| 1-mth | 110 | 4·18±1·6 | 0·2(0·06, 0·33) | 110 | 4·72±1·79 | 0·2(0·06,0·33) | 108 | 4·75±1·84 | 0·2(0·06, 0·33) | 111 | 4·24±1·75 | 0·2(0·06,0·33) | | |
| 2-mth | 111 | 4·19±1·56 | 0·27(0·13,0·4) | 109 | 4·44±1·68 | 0·27(0·13, 0·4) | 106 | 4·62±1·94 | 0·27(0·13,0·4) | 110 | 4·17±1·74 | 0·27(0·13, 0·4) | $F_{(5,323)}$ = 21·12 | **<0·0001** |
| 3-mth | 111 | 4·02±1·57 | 0·4(0·26, 0·53) | 109 | 4·26±1·85 | 0·4(0·26,0·53) | 105 | 4·37±1·71 | 0·4(0·26, 0·53) | 109 | 3·84±1·82 | 0·4(0·26,0·53) | | |
| 6-mth | 99 | 3·65±1·67 | 0·54(0·4, 0·68) | 95 | 4·24±1·58 | 0·54(0·4,0·68) | 95 | 3·86±1·77 | 0·54(0·4, 0·68) | 100 | 3·8±1·66 | 0·54(0·4,0·68) | | |
| 9-mth | 86 | 3·71±1·62 | 0·61(0·47,0·76) | 82 | 3·95±1·65 | 0·61(0·47, 0·76) | 82 | 3·54±1·76 | 0·61(0·47,0·76) | 90 | 3·71±1·97 | 0·61(0·47, 0·76) | | |

F values are from the overall contrast for group × time (4×6) interaction. Cohen *d* values are within-group time effects for baseline to each post assessment using the model mean difference divided by the whole-sample preassessment SD.

Abbreviation: AIS, Athens Insomnia Scale; HADS, Hospital Anxiety and Depression Scale; VAS, Visual Analogue Scale.

**Table S5: Secondary outcomes parameter estimates and model fit for GEE models (ITT).**

| | | HADSA | | HADSD | | AIS | | VAS-Loudness | | VAS-Annoyance | |
|---|---|---|---|---|---|---|---|---|---|---|---|
| *Predictors* | | *Estimates(95%CI)* | *p* | *Estimates(95%CI)* | *p* | *Estimates(95%CI)* | *p* | *Estimates(95%CI)* | *p* | *Estimates(95%CI)* | *p* |
| (Intercept) | | 8·57(7·76 – 9·38) | **<0·0001** | 6·77(6·10 – 7·45) | **<0·0001** | 8·42(7·47 – 9·36) | **<0·0001** | 4·33(3·95 – 4·71) | **<0·0001** | 4·09(3·71 – 4·48) | **<0·0001** |
| Group | UM | Ref | Ref | Ref | Ref | Ref | Ref | Ref | Ref | Ref | Ref |
| | UM+NBN | -0·19(-0·64 – 0·27) | 0·42 | 0·01(-0·38 – 0·39) | 0·97 | -0·84(-1·32 – -0·35) | **0·0008** | 0·34(0·14 – 0·54) | **0·0008** | 0·26(0·05 – 0·46) | **0·014** |
| | HFEM | -0·5(-0·95 – -0·06) | **0·027** | -0·38(-0·77 – 0·01) | 0·057 | -0·57(-1·11 – -0·03) | **0·039** | 0·21(0·00 – 0·41) | **0·045** | 0·22(0·01 – 0·43) | **0·040** |
| | DFCRS | -0·72(-1·17 – -0·28) | **0·0014** | -0·43(-0·81 – -0·04) | **0·030** | -1·15(-1·67 – -0·62) | **<0·0001** | -0·32(-0·52 – -0·12) | **0·0019** | 0·08(-0·12 – 0·29) | 0·43 |
| Time | Time | -0·15(-0·20 – -0·10) | **<0·0001** | -0·13(-0·17 – -0·08) | **<0·0001** | -0·3(-0·35 – -0·24) | **<0·0001** | -0·08(-0·11 – -0·06) | **<0·0001** | -0·1(-0·13 – -0·08) | **<0·0001** |
| Sex | Male | Ref | Ref | Ref | Ref | Ref | Ref | Ref | Ref | Ref | Ref |
| | Female | -0·18(-0·51 – 0·15) | 0·29 | -0·13(-0·41 – 0·16) | 0·38 | 0·77(0·39 – 1·14) | **0·0001** | 0·05(-0·09 – 0·20) | 0·47 | -0·13(-0·28 – 0·02) | 0·096 |
| Age | Age | -0·01(-0·02 – -0·00) | **0·038** | 0(-0·01 – 0·01) | 0·80 | 0(-0·01 – 0·01) | 0·89 | 0(-0·00 – 0·01) | 0·13 | 0(-0·00 – 0·01) | 0·74 |
| Duration | Duration | 0(-0·01 – -0·00) | **0.0003** | 0(-0·01 – -0·00) | **0.0002** | 0(-0·00 – 0·00) | 0·96 | 0(0·00 – 0·00) | **0·016** | 0(-0·00 – 0·00) | 0·29 |
| Location | Unilateral | Ref | Ref | Ref | Ref | Ref | Ref | Ref | Ref | Ref | Ref |
| | Bilateral | -2·04(-2·37 – -1·70) | **<0·0001** | -1·9(-2·18 – -1·62) | **<0·0001** | -1·93(-2·31 – -1·56) | **<0·0001** | -0·17(-0·32 – -0·01) | **0·035** | -0·31(-0·47 – -0·15) | **0·0002** |
| | Head | -0·55(-1·99 – 0·90) | 0·46 | -0·75(-2·00 – 0·49) | 0·24 | 0·24(-1·28 – 1·75) | 0·76 | -0·14(-0·80 – 0·53) | 0·69 | -0·4(-0·93 – 0·14) | 0·15 |
| Tone | High pitched | Ref | Ref | Ref | Ref | Ref | Ref | Ref | Ref | Ref | Ref |
| | Low pitched | -0·38(-0·79 – 0·02) | 0·065 | -0·37(-0·72 – -0·03) | **0·034** | -0·42(-0·91 – 0·06) | 0·085 | -0·17(-0·34 – 0·01) | 0·059 | -0·02(-0·20 – 0·16) | 0·81 |
| | Noise-like | -2·09(-3·21 – -0·97) | **0.0002** | -1·56(-2·53 – -0·60) | **0·0015** | -1·66(-2·91 – -0·40) | **0·010** | -0·53(-1·39 – 0·33) | 0·23 | 0·15(-0·73 – 1·04) | 0·74 |
| Type | Intermittent | Ref | Ref | Ref | Ref | Ref | Ref | Ref | Ref | Ref | Ref |
| | Persistent | 0·56(0·02 – 1·11) | **0·043** | 0·77(0·33 – 1·21) | **0·0007** | 1·36(0·80 – 1·93) | **<0·0001** | 0·85(0·58 – 1·13) | **<0·0001** | 1·07(0·81 – 1·33) | **<0·0001** |

Abbreviation: AIS, Athens Insomnia Scale; HADS, Hospital Anxiety and Depression Scale; VAS, Visual Analogue Scale.

**Table S6: The results for the additional exploratory analysis using the established MCID for THI.**

| Arms | Total | Δ THI⩽-7 Events (%) |
|---|---|---|
| UM | 111 | 92(82·88%) |
| UM+NBN | 110 | 74(67·27%) |
| HFEM | 108 | 81(75·0%) |
| DFCRS | 111 | 90(81·08%) |

**Table S7. Generalized Estimating Equations model affecting the efficacy of tinnitus sound therapy (PPS).**

| | | Adjusted model | | |
|---|---|---|---|---|
| *Predictors* | | *Estimates* | *CI* | *p* |
| (Intercept) | | 35·18 | 31·58 – 38·78 | **<0·0001** |
| Group | UM | Ref | Ref | Ref |
| | UM+NBN | 0·15 | -1·84 – 2·14 | 0·88 |
| | HFEM | 1·91 | -0·17 – 3·98 | 0·072 |
| | DFCRS | -4·37 | -6·32 – -2·43 | **<0·0001** |
| Time | Time | -1·19 | -1·43 – -0·95 | **<0·0001** |
| Sex | Male | Ref | Ref | Ref |
| | Female | 0·8 | -0·66 – 2·26 | 0·28 |
| Age | Age | 0·1 | 0·06 – 0·15 | **<0·0001** |
| Duration | Duration | -0·02 | -0·04 – -0·00 | **0·034** |
| Location | Unilateral | Ref | Ref | Ref |
| | Bilateral | -3·71 | -5·31 – -2·10 | **<0·0001** |
| | Head | -4·84 | -8·80 – -0·89 | **0·016** |
| Tone | High pitched | Ref | Ref | Ref |
| | Low pitched | 1·79 | -0·04 – 3·61 | 0·055 |
| | Noise-like | 2·85 | -5·64 – 11·35 | 0·51 |
| Type | Intermittent | Ref | Ref | Ref |
| | Persistent | 10·01 | 7·55 – 12·46 | **<0·0001** |

**Table S8: Secondary outcomes parameter estimates and model fit for GEE models (PPS).**

| | | HADSA | | HADSD | | AIS | | VAS-Loudness | | VAS-Annoyance | |
|---|---|---|---|---|---|---|---|---|---|---|---|
| *Predictors* | | *Estimates (95%CI)* | *p* | *Estimates(95%CI)* | *p* | *Estimates(95%CI)* | *p* | *Estimates(95%CI)* | *p* | *Estimates(95%CI)* | *p* |
| (Intercept) | | 8·65(7·80 – 9·50) | **<0·0001** | 6·96(6·25 – 7·66) | **<0·0001** | 8·55(7·58 – 9·53) | **<0·0001** | 4·28(3·89 – 4·67) | **<0·0001** | 4·1(3·71 – 4·49) | **<0·0001** |
| Arm | UM | Ref | Ref | Ref | Ref | Ref | Ref | Ref | Ref | Ref | Ref |
| | UM+NBN | -0·2(-0·68 – 0·28) | 0·42 | -0·18(-0·58 – 0·23) | 0·39 | -0·9(-1·41 – -0·40) | **0.0005** | 0·31(0·10 – 0·51) | **0·0030** | 0·26(0·05 – 0·46) | **0·017** |
| | HFEM | -0·56(-1·03 – -0·09) | **0·020** | -0·44(-0·84 – -0·03) | **0·036** | -0·49(-1·05 – 0·07) | 0·087 | 0·25(0·04 – 0·46) | **0·017** | 0·26(0·04 – 0·47) | **0·018** |
| | DFCRS | -0·7(-1·17 – -0·23) | **0·0033** | -0·48(-0·89 – -0·08) | **0·02** | -1·1(-1·65 – -0·56) | **0·0001** | -0·31(-0·52 – -0·11) | **0·0025** | 0·11(-0·10 – 0·32) | 0·32 |
| Time | Time | -0·13(-0·18 – -0·07) | **<0·0001** | -0·11(-0·16 – -0·06) | **<0·0001** | -0·28(-0·34 – -0·22) | **<0·0001** | -0·08(-0·10 – -0·05) | **<0·0001** | -0·1(-0·12 – -0·07) | **<0·0001** |
| Sex | Male | Ref | Ref | Ref | Ref | Ref | Ref | Ref | Ref | Ref | Ref |
| | Female | -0·23(-0·58 – 0·12) | 0·20 | -0·17(-0·47 – 0·12) | 0·25 | 0·82(0·43 – 1·20) | **<0·0001** | 0·05(-0·09 – 0·20) | 0·48 | -0·12(-0·28 – 0·03) | 0·12 |
| Age | Age | -0·01(-0·02 – 0·00) | 0·078 | 0(-0·01 – 0·01) | 0·88 | 0(-0·01 – 0·01) | 0·96 | 0(-0·00 – 0·01) | 0·12 | 0(-0·00 – 0·01) | 0·83 |
| Duration | Duration | 0(-0·01 – -0·00) | **0·0008** | 0(-0·01 – -0·00) | **0·0048** | 0(-0·00 – 0·00) | 0·58 | 0(0·00 – 0·00) | **0·026** | 0(-0·00 – 0·00) | 0·43 |
| Location | Unilateral | Ref | Ref | Ref | Ref | Ref | Ref | Ref | Ref | Ref | Ref |
| | Bilateral | -2·11(-2·47 – -1·75) | **<0·0001** | -1·99(-2·29 – -1·69) | **<0·0001** | -1·93(-2·32 – -1·55) | **<0·0001** | -0·15(-0·31 – 0·01) | 0·063 | -0·3(-0·46 – -0·14) | **<0·0001** |
| | Head | -0·6(-2·17 – 0·98) | 0·46 | -0·45(-1·74 – 0·83) | 0·49 | 0·55(-1·05 – 2·16) | 0·50 | 0·16(-0·47 – 0·80) | 0·62 | -0·29(-0·87 – 0·28) | 0·32 |
| Tone | High pitched | Ref | Ref | Ref | Ref | Ref | Ref | Ref | Ref | Ref | Ref |
| | Low pitched | -0·3(-0·73 – 0·14) | 0·18 | -0·34(-0·70 – 0·03) | 0·074 | -0·39(-0·89 – 0·11) | 0·13 | -0·15(-0·32 – 0·03) | 0·11 | 0(-0·18 – 0·18) | 0·99 |
| | Noise-like | -2·56(-3·75 – -1·37) | **<0·0001** | -1·79(-2·92 – -0·67) | **0·0018** | -1·69(-3·02 – -0·35) | **0·013** | -0·18(-1·09 – 0·73) | 0·69 | 0·34(-0·62 – 1·30) | 0·48 |
| Type | Intermittent | Ref | Ref | Ref | Ref | Ref | Ref | Ref | Ref | Ref | Ref |
| | Persistent | 0·53(-0·06 – 1·13) | 0·078 | 0·65(0·17 – 1·13) | **0·0082** | 1·25(0·65 – 1·84) | **<0·0001** | 0·87(0·59 – 1·15) | **<0·0001** | 1·05(0·78 – 1·32) | **<0·0001** |

**Supplementary File: Appendix 1**

## Protocol

# Efficacy of modified sound therapy for chronic subjective tinnitus: A multicenter, double-blind, randomized controlled trial (MOST trial)

Original Protocol Version 1: March 24, 2020

Amendment Version No.: Version 2.0

Version Date: Augst 18, 2020

Principal Investigator: Huawei Li

Affiliation of Principal Investigator: Eye & ENT Hospital Affiliated to Fudan University

# Study Summary

| | |
|---|---|
| **Study Title** | Efficacy of modified sound therapy for chronic subjective tinnitus: A multicenter, double-blind, randomized controlled trial (MOST trial) |
| **Study Subjects** | (1) Aged from 18 to 80 yrs; (2) Chronic tinnitus ( ≥ 3 months); (3) the middle ear function is normal, with pure-tone average (PTA) (0.5, 1, 2 kHz) of the worse ear less than 55 dB HL. |
| **Study Objective** | To study the safety and clinical effects of different acoustic stimulation strategies in tinnitus treatment, and to explore and determine the optimal tinnitus treatment plan. |
| **Study Type** | Multicenter randomized controlled study |
| **Study Design** | This is a multicenter randomized controlled study, sponsored by the Eye & ENT Hospital Affiliated to Fudan University, and conducted at 3 university teaching hospitals in China. Adult chronic tinnitus patients will be enrolled in the study and randomized to receive four different groups of sound therapy. The four interventions are unmodified music (UM), combination of UM and narrowband noise centered at tinnitus pitch (UM+NBN), high frequency- enhanced music (HFEM), and digital frequency customized relieving sound (DFCRS), respectively. The participants are asked to apply the sound therapy for over 2 hours per day for 9 months, followed by assessment data collection and telephone interviews since the intervention began at 5 assessment points (1 month, 2 months, 3 months, 6 months, and 9 months). The primary and secondary outcomes of different sound therapies will be observed in the four groups. Additionally, the duration of follow up will be extended for another 3 months to observe the after-effects following the termination of sound therapy. |
| **Planned Enrollment** | 400 cases |
| **Principal Investigator** | Huawei Li |

| | |
|---|---|
| **Affiliation of Principal Investigator** | Eye & ENT Hospital Affiliated to Fudan University |
| **Source of Case Data** | Adult chronic tinnitus patients who meet the inclusion criteria but do not meet the exclusion criteria |
| **Outcome measurements** | Primary outcome: THI<br>Secondary outcomes: HADS-A, HADS-D, AIS, VAS loudness and annoyance |

# Table of Contents


**1. List of Abbreviations** ... 6

**2. Background and Study Rationale** ... 6

2.1 Background ... 6

2.2 Study Rationale ... 8

**3 Study Objective** ... 9

Objective 1: ... 9

Objective 2: ... 9

Objective 3: ... 9

Objective 4: ... 10

**4. Outcome Measures** ... 10

4.1 Outcomes ... 10

4.2 Primary outcome: ... 13

4.3 Secondary outcomes ... 14

4.4 End Points: ... 14

**5. Study Subjects** ... 14

5.1 Inclusion Criteria: ... 14

5.2 Exclusion criteria: ... 15

5.3 Completion Criteria ... 15

5.4 Screen Failures Criteria ... 16

**6. Study Design** ... 17

6.1 Study Outline ... 17

6.2 Study setting in the four study arms ... 18

6.3 Study design overview and Flow Chart ... 19

6.4 Planed duration of study ... 21

**7. Study Procedures** ... 25

7.1 Screening Period ... 26

7.2 Preparatory Period with Enrollment Confirmation ... 28

7.3 Trial Session ... 28

7.4 Follow-Up Period and Study Termination ... 29

**8. Sound Treatment** ... 31

8.1 Active and control treatment ... 31

8.2 The methods of sound modulation ... 32

**9. Randomized and Blinding ..... 35**
9.1 Randomization and Participant Numbering ..... 35
9.2 Blinding and Bias Minimization ..... 36
**10. Risks ..... 37**
10.1 Non sound treatment Related Risks ..... 37
10.2 Sound treatment Related Risks ..... 38
10.3 Monitoring ..... 38
**11. Statistical Analysis ..... 39**
11.1 Definition and Selection of Statistical Analysis Data Set ..... 39
11.2 Sample Size ..... 40
11.3 Statistical Analysis Method ..... 41
11.4 Sensitivity Analyses ..... 42
11.5 Plans for Interim Analysis ..... 42
11.6 Data Confidentiality ..... 42
11.7 Safety Assessment ..... 42
11.8 Reporting ..... 43
**12. Study Management ..... 43**
12.1 Trial management and quality control ..... 43
12.2 Ethics and disseminations ..... 44
12.3 Pre-study Training ..... 44
12.4 Quality Control for Data Collection ..... 44
**13. Patient Privacy and Data Protection ..... 45**
**14. Participating Sites in the Multicenter Study ..... 45**
**15. Trial status ..... 45**
**16. Reference ..... 46**
**17. Appendix ..... 49**

# 1. List of Abbreviations

AEs, adverse effects

AIM, acoustic immittance measurements

AIS, Athens Insomnia Scale

CRF, case report form

CIs, confidence intervals

DMC, data monitoring committee

DFCRS, digital frequency customized relieving sound

GEE, generalized estimating equations

HFEM, high-frequency enhanced music

HADS, Hospital Anxiety and Depression Scale

ICF, Informed Consent Form

IQR, interquartile ranges

IR, Independent Rater

IRB, institutional review board

ITT, intention-to-treat

LOCF, last observation carried forward

MCAR, missing values are assumed to occur completely at random

MOST, Modified Sound Therapy

PPS, Per Protocol Set

PM, pitch match

PTA, Pure Tone Audiometry

SS, Safety Set

THI, Tinnitus Handicap Inventory

TM, Tinnitus masking

TRT, tinnitus retraining therapy

UM, unmodified music

UM + NBN, unmodified music combined with narrowband noise

VAS, Visual Analogue Scale

# 2. Background and Study Rationale

## 2.1 Background

Tinnitus is an auditory perception of sound in the absence of a corresponding external acoustic stimulus [1], which has a global prevalence of approximately 10% - 25% among

the adult population[2, 3]. The reported prevalence in children and adolescents was estimated 6%-41.9%, when both hearing loss and normal hearing were included [4]. Chronic tinnitus lasting more than three months often induces negative emotions, including anxiety and depression [5, 6]. Psychologically, insomnia or sleep impairments are highly prevalent in patients with tinnitus, and the most severe sleep disorder is often associated with anxiety plus depression [7].

The subjective nature of tinnitus, combined with its diverse etiological factors, makes it a complex condition to manage and treat effectively [8]. Tinnitus might develop as a consequence of changes that occur in central auditory pathways and other brain regions when the brain loses its input from the ear [9]. The non-auditory cortex network, such as limbic system, emotional center, "salience" network and memory center, also extensively participate in the generation and progression of tinnitus [10], as well as in the reinforcement of the associated distress, anxiety and depression, which in turn aggravate tinnitus, forming a vicious cycle [10, 11]. Therefore, the timely and precise intervention is advocated in tinnitus treatment, whereby such vicious cycle and tinnitus progress could be terminated in due course.

While current therapeutic strategies vary, sound therapy has emerged as a particularly promising approach due to its non-invasive nature and potential to directly address the auditory symptoms of tinnitus [12]. The perception of tinnitus is believed to be the result of neural changes within the auditory system, which may be influenced by auditory input, cognitive processes, and emotional status [13]. Sound therapy aims to provide auditory stimulation or noise masking that can potentially recalibrate the neural mechanisms responsible for tinnitus perception, thereby reducing its severity or the distress associated with it. Various forms of sound therapy have been proposed, ranging from broad-spectrum noise generators to customized sound therapies tailored to individual auditory profiles [14].

Sound therapy can be broadly categorized into two main types: unmodulated and modulated sound therapies [15]. Tinnitus masking (TM) and tinnitus retraining therapy (TRT) are the two most commonly used unmodulated strategies, and their ultimate goal is to help patients with tinnitus become accustomed to tinnitus, attenuating excessive attention to it and, thus, reduce the tinnitus-related negative emotions [16-19]. Although some findings have confirmed the effectiveness of TRT or TM in improving the quality of life of tinnitus patients via tinnitus assessment scales [20], a randomized clinical trial showed no significant difference in outcomes between TRT and placebo-sound treatment

control groups [21]. Additionally, the current unmodulated sound therapy does not appear to change the loudness of tinnitus, limiting the possibility of tinnitus elimination [22].

Conversely, customized sound therapy is designed to encourage the reorganization of abnormal neural connections or to inhibit pathological neural activity, which is more promising in effectively eliminating tinnitus [23, 24]. Current research has confirmed the superior effectiveness of customized sound therapy over unmodulated therapy [25]. For example, neuromonics tinnitus treatment involves adding or excluding individual broadband frequency sounds to relaxing music to produce intermittent tinnitus perception and facilitate desensitization to tinnitus [26-28]. It is worth noting that the tailor-made notched music training proposed by Pantev and the tinnitus pitch-matched therapy proposed by Davis used two opposite sound processing strategies; however, both produced positive results [29-31]. Thus, the underlying mechanisms of tinnitus alleviation, which might be linked to the pathogenesis of tinnitus, should be elucidated in the future.

Based on the accumulated explorations on the relevant background and our clinical experience in tinnitus diagnosis and treatment, we presume that the customized acoustic therapy, coupled with tinnitus specific psychological counseling, might be powerful for persistent tinnitus relief. In our previous study [32], we have development a digital frequency customized relieving sound (DFCRS), also named as modified tinnitus relieving sound, which involves creating a sound profile that exactly matches the pitch and quality of the tinnitus perceived by the individual. By aligning the therapeutic sound with the specific characteristics of the tinnitus, it's possible to more effectively alter the brain's perception of the tinnitus, leading to potential long-term habituation or attenuation of the intrusive sound.

## 2.2 Study Rationale

Despite the widespread use of sound therapy in clinical practice, treatment outcomes vary significantly. This variability may stem from the non-standardized nature of therapy application and the lack of understanding regarding the most effective types of sound therapy for specific patient demographics and tinnitus characteristics. Additionally, factors such as age, tinnitus duration, and tinnitus pitch may influence treatment efficacy,

but these factors are not well understood, leading to a "one size fits all" approach in many therapeutic settings.

The purpose of this multicenter trial is to conduct a prospective randomized controlled trial (RCT) to compare the effectiveness of different sound therapy strategies, including unmodified music (UM), unmodified music combined with narrowband noise (UM+NBN), high-frequency enhanced music (HFEM), and DFCRS. By investigating these modalities across a diverse cohort, we will conduct the Modified or Unmodified Sound Therapy on Patients with Subjective Tinnitus (MOST) trial. This study aims to delineate how individual variations in tinnitus characteristics and demographic factors influence the therapeutic outcomes of sound therapies.

Furthermore, the study seeks to establish a more systematic approach to selecting and customizing sound therapy based on specific patient profiles, enhancing the precision and effectiveness of tinnitus management. By addressing the current gaps in knowledge and practice, this trial has the potential to significantly improve clinical outcomes for patients with subjective tinnitus.

# 3 Study Objective

## Objective 1:

To evaluate the effectiveness and safety of sound therapy for adult patients with chronic tinnitus, focusing on improvements in sleep quality, psychological distress, and overall quality of life.

## Objective 2:

To identify the optimal sound therapy strategy for tinnitus management by comparing the efficacy of four different acoustic treatments:

- Unmodified music (UM)
- Unmodified music combined with narrowband noise (UM + NBN)
- High-frequency enhanced music (HFEM)
- Digital frequency customized relieving sound (DFCRS)

## Objective 3:

To investigate the factors that influence the effectiveness of tinnitus treatment, including individual characteristics such as age, tinnitus duration, tinnitus pitch, and so on.

## Objective 4:

To investigate the follow-up effects (extended for 3 months after treatment) of different sound therapy strategies for chronic subjective tinnitus.

# 4. Outcome Measures

## 4.1 Outcomes

### 4.1.1 Tinnitus Handicap Inventory (THI)

The Tinnitus Handicap Inventory (THI) is a self-assessment tool designed to quantify the impact of tinnitus on a person's daily life. It consists of 25 items that evaluate three key dimensions of tinnitus-related distress:

- Functional: How tinnitus affects routine tasks and overall functionality.
- Emotional: The emotional response to tinnitus, such as feelings of frustration or anxiety.
- Catastrophic: Extreme reactions, such as hopelessness or an inability to cope with the condition.

Each item offers three answer choices:

- Yes (4 points),
- Sometimes (2 points),
- No (0 points).

The total score is obtained by summing the responses across all categories, resulting in a score that classifies the severity of tinnitus into five levels:

- Slight: 0–16 points
- Mild: 18–36 points
- Moderate: 38–56 points
- Severe: 58–76 points

- Catastrophic: 78–100 points

A higher score indicates greater tinnitus-related handicap. The THI provides a reliable measurement of tinnitus severity, with strong internal consistency and construct validity. Importantly, the results are not significantly influenced by factors such as age, gender, or hearing loss, making it a robust tool for diverse patient groups.

#### 4.1.2 Hospital Anxiety and Depression Scale (HADS)

The Hospital Anxiety and Depression Scale (HADS) is designed to assess negative emotional states related to tinnitus, focusing on anxiety and depression. The scale consists of two subscales:

- HADS-A: Measuring anxiety (7 items)
- HADS-D: Measuring depression (7 items)

Each item is scored on a 0 to 3 scale, with the total score for each subscale ranging from 0 to 21. Based on the score, patients are classified into three categories:

- Asymptomatic: 0–7 points
- Suspicious Symptoms: 8–10 points
- Definite Symptoms: 11–21 points

A score of 8 or more on either subscale is considered a positive indication of anxiety or depression. This tool is widely used in clinical settings to assess the emotional impact of conditions like tinnitus and can help guide treatment decisions.

#### 4.1.3 Athens Insomnia Scale (AIS)

The Athens Insomnia Scale (AIS) is specifically designed to measure the severity of insomnia symptoms, which are frequently reported by tinnitus sufferers. Insomnia can exacerbate tinnitus-related distress by increasing anxiety and reducing the ability to cope with the condition.

The AIS evaluates three main categories of sleep disturbance:

- Sleep Initiation: Difficulty falling asleep.
- Sleep Maintenance: Difficulty staying asleep or waking up during the night.

- Early Morning Awakening: Waking up earlier than desired and being unable to return to sleep.

Each of the 8 items is rated on a scale from 0 to 3, with the total score reflecting the overall severity of insomnia. The scale is divided into the following severity categories:

- 0–3: No insomnia
- 4–6: Suspected insomnia
- 7–24: Definitive insomnia

The AIS is a reliable and valid tool for assessing sleep issues in tinnitus patients. Given the strong association between insomnia and tinnitus, identifying and treating sleep disorders can play a crucial role in improving tinnitus management and enhancing quality of life. Insomnia and tinnitus often create a vicious cycle, where poor sleep worsens tinnitus perception, and tinnitus, in turn, disrupts sleep. Thus, addressing sleep disorders is a key component of comprehensive tinnitus care.

#### 4.1.4 Visual Analogue Scale (VAS) for tinnitus

The Visual Analogue Scale (VAS) is a simple tool used to measure the perceived severity of tinnitus. It is commonly used in clinical settings to quantify subjective symptoms such as pain or discomfort. In this context, VAS is used to evaluate the subjective loudness and degree of annoyance caused by tinnitus.

Patients are asked to assign a score on a scale from 0 to 10, with 0 representing no tinnitus-related distress and 10 indicating extreme distress. The scale takes into account both the loudness and intrusiveness of the tinnitus.

While VAS is easy to administer and understand, it offers only a superficial assessment. The results can be influenced by factors such as cultural background, intellectual ability, or mental health status. Despite these limitations, VAS provides a quick and effective way to gauge the impact of tinnitus on a patient's life, with higher scores indicating more severe distress.

#### 4.1.5 Pure tone audiometry (PTA)

PTA is performed using an audiometer (Madsen Astera; GN Otometrics, Germany) with headphones (HDA-300 over the ear; Sennheiser). This test is crucial for determining the

extent of hearing loss in tinnitus patients, as there is often a close relationship between the two conditions. In this test, patients are presented with tones at various frequencies (ranging from 125 Hz to 8000 Hz) and asked to indicate when they can hear each tone.

For this study, the relevant frequencies analyzed include 500 Hz, 1000 Hz, and 2000 Hz. The pure-tone average (PTA) is calculated by averaging the hearing thresholds at these frequencies in the worse-hearing ear. A PTA of 20 dB or greater is classified as hearing loss, which is considered a disabling hearing impairment based on WHO hearing-impairment grading[33]. To ensure that participants can adequately perceive the sound therapy, the PTA threshold for inclusion in the study is set at 55 dB or lower in the worse-hearing ear.

#### 4.1.**6 Acoustic Immittance Measurement (AIM)**

Acoustic Immittance Measurement (AIM), often referred to as tympanometry, is used to evaluate the status of the middle ear and tympanic membrane. This test measures the mobility, mass, and resistance of the middle ear system by assessing how sound energy is transmitted through the ear. The results provide valuable information about the outer ear, ossicular chain, stapedius muscle, cochlea, and even the brainstem.

Tympanometry is performed using an impedance audiometer with a 226 Hz probe tone. The results are classified using the Liden and Jerger system[34, 35], which categorizes tympanograms into different types:

- Type A: Normal tympanogram, indicating normal middle ear function. Patients with Type A tympanograms are included in this study.
- Type B: Flat tympanogram, often indicative of middle ear pathology such as fluid or infection.
- Type C: Negative pressure (less than −100 daPa), usually associated with eustachian tube dysfunction.

### 4.2 Primary outcome:

The primary objective is to evaluate the THI scores (range, 0-100) over the 9-month follow-up period.

### 4.3 Secondary outcomes

The secondary outcomes mainly include the following:

- to evaluate perceived tinnitus loudness, assessed using a 0–10 VAS
- to evaluate anxiety and depression levels in tinnitus patient with Hospital Anxiety and Depression Scale.
- to evaluate influence of tinnitus on daily life, assessed using a 0–10 VAS
- to evaluate sleep quality, measured by the AIS-8

### 4.4 Endpoints:

This study defines two primary endpoints:

1) Tinnitus cure: Defined as a THI score of 0 concurrent with a VAS-Loudness score of 0, indicating complete resolution of symptoms and functional impact, thereby reaching the observation and treatment endpoint.
2) Treatment completion: The completion of the nine-month sound therapeutic intervention period, irrespective of symptomatic outcome.

## 5. Study subjects

### 5.1 Inclusion criteria:

To be enrolled in the study, participants must successfully complete the screening process, meet all eligibility criteria, and agree to comply with all procedures. The inclusion criteria for the study are as follows:

- Age: Participants must be between 18 and 80 years old.
- Tinnitus Complaint: Participants must report frequent occurrences of subjective tinnitus lasting at least 5 minutes per episode[37], with symptoms persisting for at least 3 months;
- Hearing Status: Participants must have normal hearing or mild-to-moderate hearing loss, as defined by the World Report on Hearing (2021)[33], of which the average value of hearing threshold at 500, 1000, 2000 Hz in the worse ear is less than 55 dB HL;

- Middle Ear Function: Participants must have a normal Acoustic Immittance Measurement (AIM) result, indicating normal middle ear function;
- Language: Participants must be able to communicate in Mandarin;
- Informed Consent: Participants must be fully informed about the study and agree to participate, including undergoing all treatments, assessments, and follow-up visits relevant to the study.

## 5.2 Exclusion criteria:

The following exclusion criteria are established to ensure participant safety and the consistency of the study sample. Some of these criteria may limit the generalizability of the findings but are necessary for maintaining uniformity in the participant pool:

- Tinnitus Type: Patients with vascular pulsatile tinnitus or objective tinnitus are excluded.
- Ear or Noise Trauma: A history of middle ear pathology, significant noise exposure, noise trauma, or head injury disqualifies participants.
- Major Health Conditions: Participants with any major health condition that could interfere with study participation or follow-up will be excluded.
- Associated Conditions: Patients with other medical conditions that could induce or influence tinnitus, such as infections, tumors, trauma, otosclerosis, Meniere's disease, or sudden hearing loss, are ineligible.
- Severe Mental Health Issues: Participants with severe anxiety, depression, or other mental health conditions that require referral to a psychiatric clinic will be excluded.
- Other Clinical Trials: Individuals currently enrolled in other clinical trials are not eligible to participate.

## 5.3 Completion Criteria

Participants will be classified as complete or evaluable based on the following criteria:

- ✓ Evaluable for Intent-to-Treat (ITT) Analysis: A participant is considered evaluable for the ITT analysis if they have completed at least 1 month of treatment and one THI assessment beyond the baseline.
- ✓ Evaluable and Completed Per-Protocol (PP): A participant is considered evaluable and completed per-protocol if they have completed all 9 months of treatment and all 9-month of follow-up assessments as scheduled. These participants will be included in both the ITT analysis and the PP analysis sets.
- ✓ Evaluable with Early Termination: A participant is considered evaluable with early termination if they have completed at least 1 month of treatment and one THI assessment beyond the baseline but terminated the study early. These participants will be included in the ITT analysis.
- ✓ Good Standing with Clinical Site: A participant is considered in good standing with the clinical site if, in the opinion of the investigator or therapy team, the participant complied with protocol requirements, even if they did not complete all study visits.

## 5.4 Screen Failures Criteria

Screen Failures are participants who pass the initial screening but are later deemed ineligible before being enrolled in the study. Screen failures may occur for one or more of the following reasons:

- Failure to Meet Inclusion Criteria: Participants who do not meet all of the inclusion criteria.
- Meeting Exclusion Criteria: Participants who meet one or more exclusion criteria.
- Withdrawal of Consent: Participants who withdraw consent before enrollment.

All potential participants who begin the screening process will be documented in a Screening Log, and the reasons for screen failure will be recorded. Screen Failures are not considered evaluable and will not be included in the study's analysis sets.

- ➢ Identification of Screen Failures: Screen failures may be identified through the review of medical history, assessments, laboratory results, or discussions with the participant.

- Confirming Ineligibility: If medical assessments suggest ineligibility, they may be repeated for confirmation. Once a participant is confirmed to be ineligible, they will be classified as a Screen Failure.
- Notification: Participants who are classified as screen failures will be informed that they are unfortunately not eligible for the study. No additional screening assessments will be scheduled for these participants.
- Referrals: If requested, screen failures may be referred to an outside therapist for further evaluation or treatment.

# 6. Study Design

## 6.1 Study Outline

This study is a multicenter, randomized controlled trial designed to evaluate the efficacy of different sound therapies in adult patients with chronic tinnitus. Participants will be randomly assigned to one of four treatment groups:

1. Unmodified Music (UM): Patients will receive standard, unmodified music as their sound therapy.
2. UM + Narrowband Noise (NBN): Patients will receive a combination of unmodified music and narrowband noise (NBN) centered at the individual's tinnitus pitch.
3. High-frequency enhanced music (HFEM): Patients will receive music tailored to emphasize high frequencies, customized for each participant.
4. Digital frequency customized relieving sound (DFCRS): Patients will receive a customized sound therapy specifically designed to match the pitch of their tinnitus.

Each type of acoustic therapy will be administered for over 2 hours per day, and the treatment will continue for a total of 9 months to assess the long-term effects of the acoustic therapies.

To assess the posttreatment effects of the acoustic therapies, the study is planned to include another 3-month treatment-free period after 9 months treatment, during which no sound therapy will be provided. This will allow researchers to observe whether the effects of the sound therapy are maintained over time after the cessation of treatment.

Outcome data will be collected at 6 assessment points throughout the study:

- Baseline (before treatment begins)
- 1 month
- 2 months
- 3 months
- 6 months
- 9 months

Both primary and secondary outcomes will be measured to evaluate the effectiveness of the different sound therapies. These outcomes will provide insights into the short-term and long-term efficacy of each treatment modality in managing tinnitus symptoms across the four groups.

Extension analysis will be conducted to assess whether therapeutic effects persist after sound therapy concludes.

## 6.2 Study setting in the four study arms

The methodology for sound therapy in the four treatment arms is outlined as follows:

1. **Enrollment and Randomization**: Adult patients with chronic tinnitus who meet the inclusion criteria and do not meet the exclusion criteria will be enrolled in the study. Participants will be randomized in a 1:1:1:1 ratio across four different sound therapy groups.

2. **Assignment to Treatment Groups:**

Participants will be assigned to one of the following four groups:

- Arm 1: Unmodified Music (UM) (Placebo Control)
- Arm 2: UM + Narrowband Noise (NBN) (Standard Control)
- Arm 3: High-frequency enhanced music (HFEM)
- Arm 4: Digital frequency customized relieving sound (DFCRS).

The detailed methods for sound modulation are shown in Section 8.2.

3. **Intervention Method:**

Acoustic stimulation is the sole intervention in this study. No pharmacological

treatments or hearing aids will be used. All participants will receive comprehensive education from trained staff regarding:

- The mechanism of tinnitus
- The theory and procedure of sound therapy
- The psychoacoustic assessment of tinnitus
- The relationship between tinnitus and attention.

4. **Sound Therapy Duration:**

Participants in all four groups will listen to their assigned sound therapy for at least 2 hours per day over a period of 9 months. After this period, participants will enter a treatment-free phase, during which they will be instructed to avoid any music stimuli to assess the sustained effects of the therapy. An extension follow-up will be scheduled at 3 months after sound treatment disclosed.

5. **Data Collection:**

The following data will be collected from participants at various stages of the study:

- Demographic characteristics
- Hearing test results
- Psychoacoustic measures of tinnitus
- Tinnitus characteristics (such as pitch and loudness)
- Tinnitus severity as assessed by standardized questionnaires

## 6.3 Study design overview and Flow Chart

In this study, participants will be randomly assigned to one of four groups to receive the following interventions:

1. Unmodified Music (UM)
2. Combination of UM and Narrowband Noise (UM + NBN), where the narrowband noise is centered at the tinnitus pitch
3. High-frequency enhanced music (HFEM)
4. Digital frequency customized relieving sound (DFCRS)

**Data Collection and Assessment Timeline**

Participants' data will be collected at six time points throughout the study:

- Baseline (T0): Before the start of treatment
- 1 month post-treatment (T1)
- 2 months post-treatment (T2)
- 3 months post-treatment (T3)
- 6 months post-treatment (T4)
- 9 months post-treatment (T5)

The study flow chart, which illustrates the recruitment process, randomization, treatment duration, and follow-up schedule, is presented in the figure below.

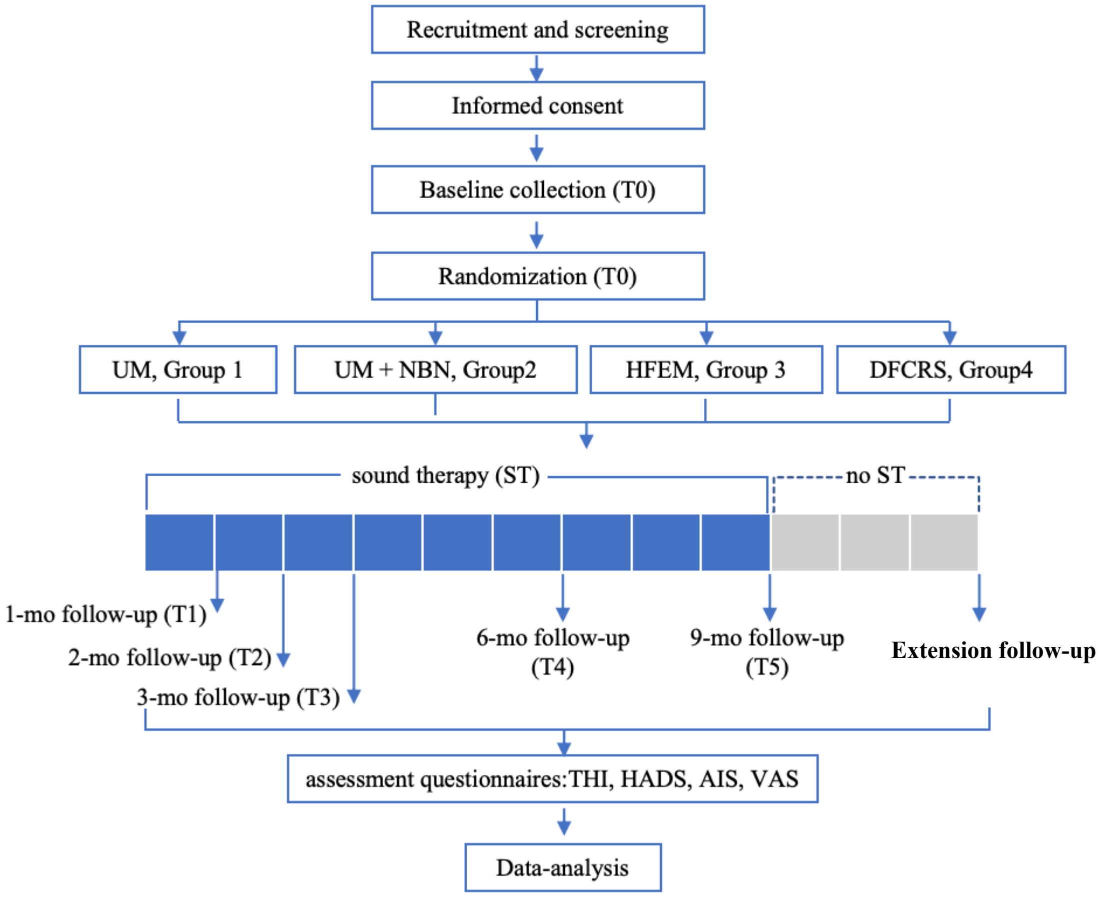


**Figure 1. Recruitment and procedure.** The participants will be enrolled according to the inclusion and exclusion criteria. Before the start of treatment, informed consent should be signed, baseline data should be collected, and the random enrollment should

be arranged. This is a 4-arm randomized clinical trial, including UM, UM+NBN, HFTM, and DFCRS, where sound treatment will last for 9 months. THI and other scales are used to evaluate the treatment effect, and finally statistical analysis will be performed.

**Note:**

1. Baseline is timepoint 0 (T0) of treatment (i.e., before the start of treatment).
2. There are total five post-treatment follow-ups (1-month-T1, 2-month-T2, 3-month-T3, 6-month-T4, and 9-month-T5).
3. Sound therapy is scheduled to last 9 months, unless the patient is lost to follow-up.

## 6.4 Planed duration of study

### 6.4.1 Study Location

This study will be conducted across multiple sites, primarily in tertiary hospitals located in Shanghai, China. The planned participating sites are as follows:

- Eye & ENT Hospital Affiliated to Fudan University (EENT Hospital)
- Yueyang Hospital of Integrative Chinese & Western Medicine, affiliated with Shanghai University of Traditional Chinese Medicine (Yueyang Hospital)
- Zhongshan Hospital Affiliated to Fudan University (Zhongshan Hospital)

Additional study sites may be included as needed to ensure the recruitment of a sufficient number of participants. The lead site for this study is the Ear, Nose, and Throat (ENT) Department at the Eye & ENT Hospital Affiliated to Fudan University, which is renowned for its expertise in otorhinolaryngology.

The study has been reviewed and approved by the ethics committee of the lead site. Ethical approval will also be obtained for any additional participating centers.

### 6.4.2 Participant Selection

Eligibility for the study will be based on a diagnosis of persistent chronic subjective tinnitus with bothersome symptoms. The screening process will include the following evaluations:

- ✓ Hearing and Tinnitus Assessments: These will involve electric otoscopy, PTA, and acoustic immittance measurements (AIM).

- ✓ Imaging Tests: If necessary, imaging tests (such as MRI or CT scans) will be conducted to rule out other middle ear pathologies.

Before treatment begins, the following data will be collected from participants:

- Demographic characteristics
- Hearing test results
- Psychoacoustic measures of tinnitus
- Details of tinnitus characteristics
- Tinnitus severity, assessed through validated questionnaires

During and after the treatment period, additional data will be collected to assess treatment efficacy and monitor for any adverse events.

This is an interventional study, and it will be conducted according to the protocol outlined in the subsequent sections.

### 6.4.3 Informed Consent

The informed consent process will take place in a private and comfortable environment where participants can receive a comprehensive explanation of the study. Under the supervision of the principal investigator, participants will be provided with detailed information regarding:

- ✓ The study objectives and purpose
- ✓ The procedures involved in the study
- ✓ The potential risks and benefits of participation
- ✓ Confidentiality measures for protecting participant data
- ✓ Participant rights, including the right to withdraw from the study at any time

Participants will be given the opportunity to ask questions and seek clarifications to ensure they fully understand the study before making a decision to participate. The process of obtaining written informed consent will follow all ethical guidelines and regulatory requirements, ensuring that participants can make an informed and voluntary decision about their involvement in the study.

### 6.4.4 Randomization and Allocation

Participants will be randomized equally across the three study centers, with a balanced distribution across the four treatment arms within each center. Stratified block randomization will be used to ensure comparability between treatment groups and to minimize selection bias.

**Stratification by Center**

Participants will first be stratified by recruitment center to ensure that each center independently achieves a balanced allocation across the four treatment arms. The allocation of participants will be based on the actual volume of tinnitus diagnosis and treatment at each center:

- EENT Hospital will recruit the largest number of participants.
- Yueyang Hospital of Integrative Chinese & Western Medicine will recruit the second-largest number of participants.
- Zhongshan Hospital Affiliated to Fudan University will recruit the remaining participants.

Within each center, participants will be assigned to one of the four treatment arms in a 1:1:1:1 ratio using block randomization. A fixed block size of four will be used to maintain balance throughout the recruitment process and to prevent prediction of future assignments.

**Randomization Procedure**

Randomization tables will be generated before the trial begins and will be concealed from both the investigators and participants to maintain the integrity of the randomization process. The allocation of participants will be managed by an independent statistician or a designated clinical research coordinator who is not involved in participant enrollment or data collection. The allocation sequence will be securely stored, and treatment group assignments will only be disclosed to the treating team upon participant enrollment.

**Blinding**

This is a double-blind trial, meaning that neither the participants nor the investigators will be aware of the treatment allocations during the study. Although participants will be

blinded, it is possible that some may deduce their group assignment, particularly those in Arm 2 (UM + NBN), due to the presence of background noise in their treatment. However, the informed consent form will provide a general description of all treatment groups without revealing specific details that would allow participants to accurately identify their group.

Blinding codes will be securely maintained and will only be broken after the completion of data analysis or in the event of a clinical emergency.

**Monitoring Randomization**

The randomization process for this study will involve block randomization with fixed block sizes (e.g., 4 participants per block), stratified by study center. The study includes three centers with different sample sizes, and the total sample size is 400 participants. Each center will have four treatment arms, and the block size ensures that each treatment group within a block has an equal number of participants. The randomization will be conducted using the blockrand() function in R, with the sample size for each center as input, along with the number of groups (4 treatment arms) and the block size (4). The randomization tables for each center will be independently generated, ensuring distinct randomization sequences per center. To ensure reproducibility, a fixed seed will be set (set.seed(123)) for the randomization process. The generated randomization tables will be stored in a list for each center and used to allocate participants while maintaining allocation concealment throughout the study. This process ensures balanced and randomized group assignments across all centers.

A sample randomization script used to generate the allocation sequence is as follows:

```
# Example R code for generating block-randomized sequence for each center
library(blockrand)
set.seed(123)    # Ensures reproducibility
# Sample sizes for each center
sample_sizes <- c(200, 140, 60)
# Generate random allocation tables per center
center_randomization <- lapply(1:3, function(center) {
  blockrand(n = sample_sizes[center], num.levels = 4, block.sizes = 4, id.prefix =
paste0("Center", center, "_"))
})
```

### 6.4.5 Intervention

Following randomization, participants will be asked to provide essential personal and clinical information, including key demographic data such as gender, age, and the date of tinnitus onset. Once randomization and baseline measurements are completed, participants will begin the intervention phase according to their assigned treatment group. Detailed instructions and procedures specific to their treatment group will be provided to ensure adherence to the intervention protocol.

### 6.4.6 Timepoints

Each enrolled participant will undergo a clinical evaluation, provide informed consent, and submit basic personal information during the baseline measurement (T0). After stratified randomization, participants will be informed of their assigned treatment group and will begin a 9-month intervention period.

The study will consist of six scheduled visits as follows:

- **Visit 1 (Baseline, T0)**: This is the screening visit, where participants provide informed consent, complete baseline assessments, and begin their assigned treatment.
- **Visit 2 (T1)**: 1-month post-enrollment, participants will attend their first post-treatment follow-up to assess initial responses to the intervention.
- **Visit 3 (T2)**: 2-month post-enrollment, a second follow-up visit will be conducted to monitor the ongoing effects of the treatment.
- **Visit 4 (T3)**: 3-month post-enrollment, participants will attend a follow-up to evaluate mid-stage outcomes of the intervention.
- **Visit 5 (T4)**: 6-month post-enrollment, this visit will serve as a key mid-trial assessment of treatment efficacy and safety.
- **Visit 6 (T5)**: 9-month post-enrollment, participants will attend their final follow-up during the active treatment phase.

These scheduled visits will allow for close monitoring of participants throughout the treatment and follow-up periods, enabling comprehensive assessments of both short-term and long-term outcomes.

# 7. Study Procedures

## 7.1 Screening Period

If a potential participant is deemed eligible based on initial criteria, they will receive a copy of the Informed Consent Form (ICF) for review and be invited to the site for an in-person screening. All individuals who are pre-screened will be assigned a Screening Number and recorded in the Screening Log.

For potential participants who do not pass the screening, their data will not be entered into the Electronic Case Report Form (eCRF). However, the reason for ineligibility will be documented in the Screening Log. If, at any point during the screening process, a participant is determined to be ineligible, they will be classified as a Screen Failure, notified of their ineligibility, and will not be scheduled for further assessments.

If significant conditions other than tinnitus are identified, the potential participant will not be eligible for enrollment. In such cases, they will be advised to seek further evaluation from their personal physician.

**Screening Assessments**

The following assessments will be conducted during the screening period:

a) Written consent must be obtained from the participant before any tests or evaluations are conducted.

b) Relevant medical and psychiatric records will be collected to allow the site physician to obtain a well-characterized medical history and assess eligibility. The participant's medical and psychiatric history will be reviewed through an interview and examination of the provided records. If records are insufficient or missing, additional records will be requested. Information on prior hospitalizations and healthcare utilization will also be collected.

c) A review of past and current medications will be conducted, along with an assessment of the participant’s adherence to prescribed treatments.

d) A pre-exam otoscopic screening will be performed to identify any abnormalities in the external ear, including the ear canal and eardrum. Excessive or impacted cerumen will be removed immediately. All participants will undergo acoustic immittance testing, and those with a type “C” or type “B” tympanogram will be excluded. Pure-tone air conduction thresholds for both ears will also be measured.

e) Participants will be directed to complete self-reported questionnaires as part of the screening process.

**Review and Eligibility Confirmation**

Once all screening results are obtained, the site team will review the medical assessments, interview notes, medical records, and test results against the study's eligibility criteria. If any questions arise regarding potential medical issues, the site physician may request additional tests, assessments, or consultations as needed. With the participant's permission, the site physician may also contact external healthcare providers for further clarification.

The site staff will provide instructions on how participants can access telemedicine visits for future assessments. Participants will be advised to conduct these visits from a private and quiet space where they feel comfortable discussing personal health matters.

### 7.1.1 Enrollment

Prior to enrollment, the site team will thoroughly review all data collected during the screening process, including:

- Screening visit notes
- Medical assessments
- Independent Rater (IR) assessments
- Medical records
- Other eligibility measures

If the participant is determined to be eligible, the appropriate sound therapy formula will be discussed. For detailed information on sound therapy, including tinnitus pitch and loudness matching, refer to **Section 8: Treatment**.

At the onset of the study at each site, once a potential participant is deemed eligible, the study team will contact the Medical Monitor and submit a summary of the participant's medical history for approval. If the Medical Monitor agrees, the participant will be officially enrolled in the study.

Once enrolled, requirements for adverse event (AE) collection will begin immediately.

### 7.2 Preparatory Period with Enrollment Confirmation

Participants will undergo Preparatory Sessions lasting approximately 10 minutes each, prior to the start of the Trial Session. These preparatory sessions will focus on providing psychoeducation about tinnitus and establishing a sense of safety and trust within the therapeutic relationship.

At any point during the Preparatory Period, if a participant is deemed ineligible, the site team will classify them as a Pre-randomization Early Termination. In such cases, the participant will be informed that they are unfortunately not eligible for the study, and no further assessments will be scheduled. Participants classified as Pre-randomization Early Terminations will not proceed to randomization.

### 7.3 Trial Session

#### 7.3.1 Baseline Assessment

After completing the Preparatory Sessions, a staff member will conduct the Baseline Assessment measures, including the THI, VAS, HADS, and AIS-8. This assessment will take approximately 10 minutes and may be recorded to ensure inter-rater reliability. The scores will be promptly sent to an independent staff member for further review.

The baseline data collection may include the following domains:

1) **Demographic characteristics**: Basic demographic data (e.g., gender, age).

2) **Tinnitus-related characteristics**: Information such as tinnitus duration, site, tone, state, and potential triggers.

3) **Tinnitus severity assessment**: Multidimensional assessment using validated questionnaires, including: Tinnitus Handicap Inventory (THI); Hospital Anxiety and Depression Scale (HADS) (subscales HADS-A for anxiety, HADS-D for depression); Athens Insomnia Scale (AIS); Visual Analogue Scale (VAS) for tinnitus severity.

4) **Audiological and imaging tests**: These may include pure tone audiometry, tympanometry, tinnitus pitch matching, and, if applicable, imaging tests such as CT or MRI.

#### 7.3.2 Independent Assessments for Confirmation

A blinded investigator will review and confirm the baseline assessments by examining the contact records. These assessments may also be recorded to ensure inter-rater

reliability. The confirmed data will be sent to a blinded coordinator for entry. Both the sponsor and the site personnel will remain blinded to the data throughout the study.

### 7.3.3 Treatment

Participants will be randomly assigned to one of four sound therapy strategies, which include two control arms and two experimental arms:

**Control Arms:**

***Unmodified Music (UM placebo control arm)*****:** Participants in this group will listen to pure, unmodulated music.

***Combination of Unmodified Music and Narrowband Noise (UM+NBN standard control arm)***: Participants in this group will listen to unmodified music mixed with 1/3 octave narrowband noise centered at the frequency of their tinnitus pitch.

**Experimental Arms:**

***High-frequency enhanced music (HFEM experimental arm)*****:** In this group, the music is modulated in the 6000-9000 Hz range, with an enhanced intensity of 10 dB, regardless of the tinnitus pitch.

***Digital frequency customized relieving sound (DFCRS experimental arm)***: Participants in this group will listen to soothing music that is modulated according to a specific algorithm. The algorithm targets two frequency bands—1/3 octave intervals below and above the tinnitus pitch—with a dynamic increase of 10 dB gain in these specified bands.

For a more detailed explanation of these strategies, refer to previous sections.

**Sound Therapy Application and Follow-Up**

All participants are required to apply their assigned sound therapy for a minimum of 2 hours per day. Following the initiation of the intervention, data collection will occur regularly through assessments and telephone interviews to monitor progress and measure outcomes.

## 7.4 Follow-Up Period and Study Termination

### 7.4.1 Follow-up Period

The study consists of a total of seven scheduled visits to monitor participants' progress and assess the long-term effects of sound therapy:

**Visit 1** (Baseline, T0): This is the initial screening visit, where informed consent is obtained, participants are enrolled, and treatment is initiated.

**Visit 2** (T1): 1 month after enrollment, a post-treatment follow-up visit will be conducted.

**Visit 3** (T2): A follow-up visit scheduled 2 months after enrollment.

**Visit 4** (T3): A follow-up visit scheduled 3 months after enrollment.

**Visit 5** (T4): A follow-up visit scheduled 6 months after enrollment.

**Visit 6** (T5): A follow-up visit scheduled 9 months after enrollment.

These follow-up visits aim to assess both the short-term and long-term effects of the sound therapy. Participants will have access to the therapy team as needed for support, and additional visits (via phone, telemedicine, or in person) can be scheduled upon request. Participants are expected to comply with all protocol requirements, including medication use, until after the study is terminated.

At each follow-up visit, multidimensional assessment questionnaires—Tinnitus Handicap Inventory (THI), Hospital Anxiety and Depression Scale (HADS), Athens Insomnia Scale (AIS), and Visual Analogue Scale (VAS)—will be administered to evaluate tinnitus severity and related outcomes. These assessments will be collected at seven key time points: baseline, and at 1-, 2-, 3-, 6-, and 9-months after the commencement of treatment.

**In detail:**

Follow-up evaluations will be conducted at 1-, 2-, 3-, 6-, and 9-months after treatment begins to assess both short-term and long-term effects of the sound therapy.

To evaluate the lasting effects of the therapy, all acoustic stimulation will be withheld after the 9th of the study. An extension assessment will be scheduled to conduct at 3 months after the cessation of sound therapy to determine whether the therapeutic effects persist.

The data collection process at follow-up visits will mirror the methods used during the baseline assessment to ensure consistency throughout the trial.

### 7.4.2 Study Termination

After all Study Termination measures and assessments are completed, the participant will be considered terminated from the study and may resume their normal daily

activities. The study team will provide an Exit Plan, which may include referrals for additional medical or therapeutic care if required.

### 7.4.3 Extension Studies

At the Study Termination visit, eligible participants will be provided with a new Informed Consent Form (ICF) for potential enrollment in future studies. Once unblinding occurs after the database lock, participants who will be assigned to control conditions, have completed the study, meet the criteria, and are in good standing with the clinical site, will be offered the opportunity to enroll in subsequent safety studies.

### 7.4.4 Exit Plan

At the conclusion of the study, participants will receive an Exit Plan, which will summarize the treatments they completed, their current medications, and provide contact information for any follow-up questions regarding the study. If appropriate, participants may request a referral for further therapeutic or medical care. Participants who terminate the study early will also receive an Exit Plan at their final contact.

### 7.4.5 Withdrawal/retention

Participants have the right to withdraw from the study at any time. Participants who no longer meet the inclusion criteria or who are lost to follow-up will also be classified as withdrawn. Researchers must document the reasons for withdrawal in detail.

Given the long follow-up period and the extended intervals between data collection time points, there is a risk of participants being lost to follow-up, which poses a technical challenge for the study. To encourage participant retention, the study will:

- Simplify the follow-up process to reduce participant burden.
- Waive any additional examination or treatment fees.
- Maintain regular contact with participants through various methods, such as phone calls, WeChat, and email, and provide timely responses to their inquiries.

These retention strategies aim to ensure participant engagement and minimize the risk of dropout throughout the study duration.

# 8. Sound Treatment

## 8.1 Active and control treatment

The active study treatment involves modulated music and includes the following two experimental arms:

- High-frequency enhanced music (HFEM experimental arm): In this group, the music is modulated within the frequency range of 6000-9000 Hz, with an intensity enhancement of 10 dB, regardless of the participant's tinnitus pitch.
- Digital frequency customized relieving sound (DFCRS experimental arm): In this arm, the soothing music is modulated based on a specific algorithm. This algorithm targets two distinct frequency bands—1/3 octave intervals below and above the participant's tinnitus pitch. These bands will be dynamically adjusted to increase intensity by 10 dB.

All participants assigned to the active treatment groups are required to apply the sound therapy for at least 2 hours per day. Data collection and follow-up through telephone interviews will be conducted regularly from the start of the intervention.

The control treatments consist of two arms:

- Unmodified Music (UM, placebo control arm): Participants in this group will listen to pure, unmodulated music.
- Combination of Unmodified Music and Narrowband Noise (UM+NBN, positive control arm): This group will listen to unmodulated music combined with 1/3 octave narrowband noise centered on the participant's tinnitus pitch.

These control treatments are designed to compare the effects of the modulated music interventions against both a placebo condition and a positive control condition.

## 8.2 The methods of sound modulation

The methodology for sound therapy in the four treatment arms is outlined as follows:

**Group 1: Unmodified Music (UM)**

Participants in this group will listen to pure, unmodulated music. The music selected has a pleasant and soothing rhythm, with no additional modifications or noise overlays. This group serves as the placebo control.

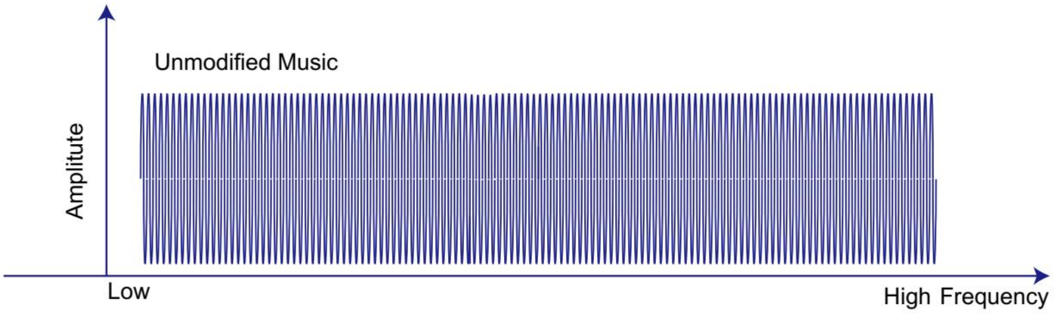


**Figure 2. Schematic diagram of unmodulated music.**

**Group 2: Combination of UM and Narrowband Noise (UM + NBN)**

Participants in this group will listen to unmodified music combined with narrowband noise (NBN). The narrowband noise will be centered on the participant's specific tinnitus frequency, using a 1/3 octave interval around the tinnitus pitch. This group serves as the positive control.

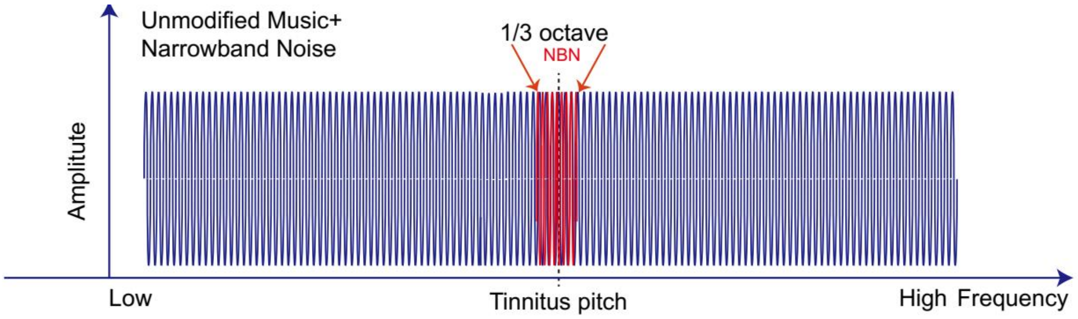


**Figure 3. Schematic diagram of unmodulated music combined with narrowband noise (NBN).** The bandwidth of NBN is 1/3 octave centered at tinnitus pitch.

**Group 3: High-frequency enhanced music (HFEM)**

In this group, participants will listen to modulated music that emphasizes a high-frequency range of 6000-9000 Hz. The intensity of the music within this frequency range will be increased by 10 dB, regardless of the participant's tinnitus pitch. This intervention aims to enhance the therapeutic effect for tinnitus patients with high-frequency hearing loss.

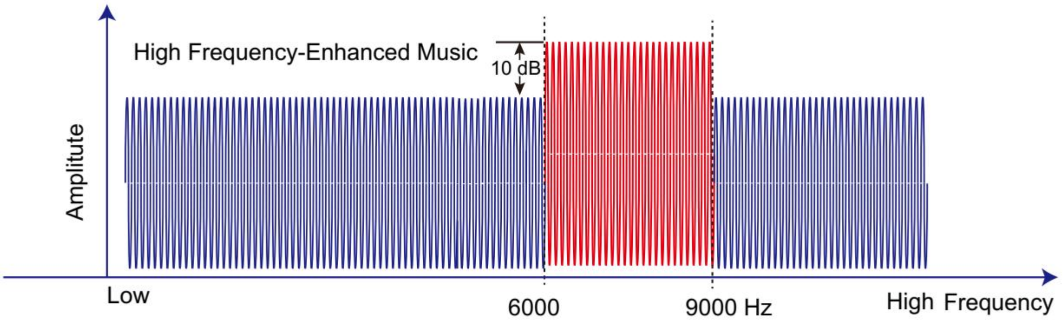

**Figure 4. Schematic diagram of high-frequency enhanced music.** The modulated range is 6000-9000 Hz, and the enhanced intensity is 10 dB.

**Group 4: Digital frequency customized relieving sound (DFCRS)**

This group will receive a customized sound therapy designed to match the participant's specific tinnitus pitch. The process for generating the sound therapy is as follows:

- **Tinnitus pitch match (PM)**: The participant's tinnitus pitch ($F_t$) will be identified by matching the tinnitus frequency to a series of tones within their audiometric range;
- **Verification of Octave Endpoints**: The upper ($F_u$) and lower ($F_l$) endpoints of one octave centered on $F_t$ will be determined. The formula for $F_t$, $F_u$, and $F_l$ will follow standard octave conversion principles as follows.

$$F_u = 2F_l \quad (1)$$

$$F_u \times F_l = F_t^2 \quad (2)$$

- **Determination of Modulated Frequency Bands**: Based on the tinnitus pitch ($F_t$), two 1/3 octave intervals will be calculated above and below the tinnitus frequency. This results in four key frequency points: $F_{uu}$ (upper endpoint of 1/3 octave centered at $F_u$), $F_{ul}$ (lower endpoint of 1/3 octave centered at $F_u$), $F_{ll}$ (lower endpoint of 1/3 octave centered at $F_l$), and $F_{lu}$ (upper endpoint of 1/3 octave centered at $F_l$).

According to the definition of the 1/3 octave, the upper ($F_{uu}$) and lower ($F_{ul}$) end points of the 1/3 octave centered at $F_u$ are:

$$F_{uu} = (2^{\frac{1}{3}})^{\frac{1}{2}} \times F_u \quad (3)$$

$$F_{ul} = (2^{\frac{1}{3}})^{-\frac{1}{2}} \times Fu \quad (4)$$

The upper ($F_{lu}$) and lower ($F_{ll}$) end points of the 1/3 octave centered at $F_l$ are:

$$F_{lu} = (2^{\frac{1}{3}})^{\frac{1}{2}} \times F_l \quad (5)$$

$$F_{ll} = (2^{\frac{1}{3}})^{-\frac{1}{2}} \times F_l \quad (6)$$

- **Music Modulation**: The intensity of the music will be dynamically enhanced by 10 dB within the two frequency bands: Fll to Flu and Ful to Fuu. This tailored modulation is designed to target the participant's specific tinnitus frequency range for therapeutic relief.

A diagram illustrating this modulation strategy will be provided to clarify the process.

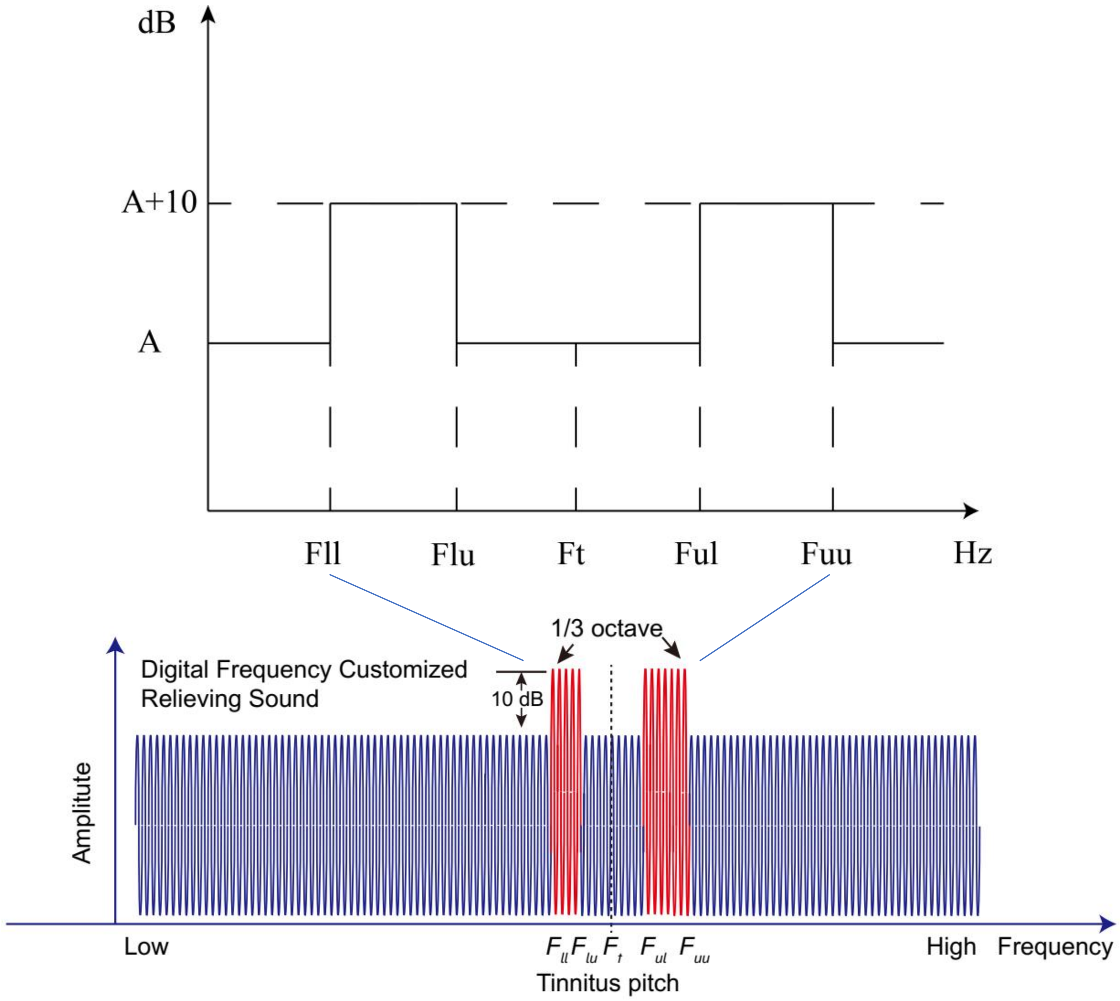


**Figure 5. Schematic diagram of digital frequency customized relieving sound.** The modulated ranges are $F_{ll}$ to $F_{lu}$ and $F_{ul}$ to $F_{uu}$, 1/3 octave each, and the enhanced intensity is 10 dB. The relationship between $F_{ll}$, $F_{lu}$, $F_{ul}$, $F_{uu}$, and $F_t$ is as above.

# 9. Randomized and Blinding

## 9.1 Randomization and Participant Numbering

Before participating in the study, all participants must provide written informed consent, which will be obtained by the Principal Investigator. The randomization process will be conducted by an independent researcher who is not involved in participant recruitment or outcome measurement.

Randomization will be implemented using block randomization with variable block sizes, generated by an independent statistician. The individual responsible for generating the allocation sequence will not participate in the recruitment of participants. The staff

member managing the randomization sequence file—who will also have no involvement in subsequent participant assessments—will notify participants of their group assignment via telephone or email.

Throughout the study, data analysts, study coordinators, and outcome assessors will remain blinded to group assignments until the primary analyses are completed, ensuring objectivity in the data analysis process.

## 9.2 Blinding and Bias Minimization

This study is designed as a double-blinded randomized controlled trial. To minimize bias, the following personnel will remain blinded throughout the trial:

Investigators will not know which group participants are assigned to.

Participants will not know which treatment strategy they are receiving.

Data analysts will be blinded to group allocations during the interpretation of study outcomes.

**Blinding Procedures**

Independent investigators from the site hospital, who are unaware of the group assignments, will conduct participant assessments. This ensures that assessments are performed without knowledge of the assigned interventions, reducing the risk of assessment bias.

The outcome assessors will also be kept unaware of the participants' assigned interventions to further mitigate the potential for biased assessments.

Data analysts involved in the interpretation of study outcomes will remain blinded to treatment groups, helping to prevent bias during data analysis. This will uphold the integrity and objectivity of the trial's findings, thereby enhancing the internal validity of the study.

To ensure the robustness of this blinding process, regular monitoring will be conducted throughout the trial to assess the integrity of the blinding procedures.

**Additional Blinding Measures**

To further enhance blinding and minimize bias, the following measures will be implemented:

- ✓ **Training**: Both data collectors and assessors will undergo thorough training to ensure high interrater reliability in their assessments. This training will help standardize the evaluation process and maintain consistency across all study sites.
- ✓ **Masking**: Whenever possible, data collectors will be masked to the treatment allocation during data collection. This will help prevent biases that could arise from knowledge of the treatment group. By keeping data collectors unaware of participants' group assignments, the study aims to reduce any unintentional influence on the data collection process.
- ✓ **Outcome Assessment**: The outcome data assessor will remain blinded to the randomization until after the statistical analyses of the experimental outcomes are completed. This ensures that the assessment of outcomes is conducted independently and without knowledge of the group allocation, further reducing the potential for bias in the evaluation of the study's results.

By rigorously implementing these blinding measures, the study aims to maximize the objectivity and reliability of its findings, minimizing assessment bias and achieving the highest level of blinding possible.

# 10. Risks

## 10.1 Non sound treatment Related Risks

As part of the study, participants will undergo several pre-treatment procedures, including a comprehensive medical examination (which may include a physical exam, ECG, etc.) to determine eligibility. These procedures carry certain inherent risks:

- ➢ **Medical Examination**: Some participants may experience discomfort or psychological distress during the full medical examination.

While these procedures are required for establishing eligibility, the study team will take all necessary precautions to minimize risks. Participants will be evaluated by the site physician, the therapy team, and the Medical Monitor to ensure they are not at risk for any severe psychosocial disorders or behaviors.

## 10.2 Sound treatment Related Risks

While sound therapy is generally considered a non-invasive and low-risk treatment, there are still potential risks associated with its use. These risks may include:

- **Hearing Discomfort or Fatigue**: Continuous or prolonged exposure to sound therapy, especially at higher volumes or for extended periods, may cause discomfort, ear fatigue, or temporary hearing sensitivity in some participants.
- **Tinnitus Fluctuations**: Some participants may experience fluctuations in their tinnitus symptoms, including temporary worsening of the condition during or after sound therapy sessions. This could result in increased tinnitus loudness or perception of new sounds, which may cause discomfort or distress.
- **Emotional or Psychological Impact**: For participants with anxiety or depression related to their tinnitus, sound therapy could unintentionally exacerbate these conditions, leading to increased emotional distress or irritability.

The therapy will be carefully monitored, and participants will be encouraged to report any discomfort or unusual symptoms. The therapy team will adjust the sound therapy parameters, such as volume or duration, to reduce the risk of adverse effects when necessary.

## 10.3 Monitoring

Throughout the study, participants will be closely monitored for any adverse effects (AEs), whether related to sound therapy or other treatments. Common symptoms that may require attention include:

Physical Symptoms:

- Dizziness
- Headache
- Blurred vision
- Shortness of breath
- Irregular heartbeat
- Numbness or tingling in the arms or legs
- Swelling in the extremities (fingers, hands, feet, or lower legs)

Psychological Symptoms:

- Mood changes (e.g., irritability, aggression, or agitation)
- Cognitive difficulties (e.g., trouble thinking, speaking, or walking)

Other Symptoms:

- Decreased urine output
- Pounding in the ears
- Difficulty breathing at rest

Participants will be encouraged to report any of these symptoms immediately. The therapy team and clinicians will work together to monitor participants closely, ensuring that any adverse effects are addressed promptly. Clinicians prescribing any accompanying medications will inform participants of the AE profile and provide guidance on how to manage potential side effects. The interprofessional communication between clinicians and the therapy team will ensure that sound therapy and any associated treatments achieve their optimal results while minimizing risks to participants.

# 11. Statistical Analysis

## 11.1 Definition and Selection of Statistical Analysis Data Set

The subjects in the trial will be categorized as follows:

**1. Intention-to-Treat Analysis Set (ITT)**

The primary analysis of the RCT will follow the intention-to-treat (ITT) principle. ITT includes all participants who are randomized into the study, regardless of whether they have completed the intervention as per the protocol, adhered to treatment, or had missing outcomes. The ITT approach preserves the benefits of randomization by maintaining participants in their originally assigned groups, allowing for an unbiased comparison that reflects real-world conditions, including protocol deviations and non-compliance. Demographics and other baseline characteristics are also set as the ITT. The missing value of the primary efficacy indicators, secondary efficacy and safety indicators will be filled with the last observation carried forward.

**2. Per Protocol Set (PPS)**

PPS comprises all the ITT subjects who meet the inclusion criteria but not the exclusion criteria and complete the treatment protocol without seriously violating the study protocol, which is the secondary analysis set for efficacy evaluation.

**3. Security data set (Safety Set, SS)**

SS includes all subjects who are block randomized, receive at least one treatment, and have at least one postbaseline safety evaluation.

## 11.2 Sample Size

The sample size for this trial is determined using Generalized Estimating Equations (GEE) tests for the slope of multiple groups in a repeated measures design with a continuous outcome. The calculation is conducted using Power Analysis Software (NCSS; Kaysville, UT, USA).

To achieve a statistical power of 90% at a significance level of 0.05, a total of 400 subjects is required, with participants divided equally across four groups (100 subjects per group). This calculation is based on a Chi-squared test with 3 degrees of freedom to evaluate the significance of differences in slope trajectories between the groups over time.

Under the alternative hypothesis, the projected slopes for the four groups are -0.23, -0.24, -0.27, and -0.30, with a residual standard deviation of approximately 0.172. Each participant will undergo 6 repeated measurements throughout the study period, with measurement intervals at specific time points (e.g., baseline, 1 month, 2 months, etc.).

This sample size allocation (100 participants per group) is expected to provide sufficient power to detect statistically significant differences in the slopes of the groups, ensuring robust and reliable results for the primary analysis.

GEE Tests for the Slope of Multiple Groups in a Repeated Measures Design (Continuous Outcome)

**Numeric Results for a Multi-Group Slope Comparison Test using GEE**
Measurement Times: Equally spaced
Correlation: Compound symmetry (all ρ's equal)
Missing Pattern: No missing data
Number of Groups: 4

| Power | N | Group Alloc Prop Set ri | Meas Times M | Time Set | Grp Slopes Set βi | SD of Grp Slopes σm | Std Dev σ | Base Corr ρ | First Row of Corr Matrix | Alpha | Prop Miss |
|---|---|---|---|---|---|---|---|---|---|---|---|
| 0.90056 | 400 | ri(1) | 6 | T(1) | βi(1) | 0.1 | 0.172 | 0.5 | ρ1(1) | 0.05 | 0 |

**Set(Set Number): Values**
ri(1): 0.25, 0.25, 0.25, 0.25
T(1): 0, 0.2, 0.4, 0.6, 0.8, 1
βi(1): -0.23, -0.24, -0.27, -0.3
ρ1(1): 1, 0.5, 0.5, 0.5, 0.5, 0.5

**References**

Ahn, C., Heo, M., and Zhang, S. 2015. Sample Size Calculations for Clustered and Longitudinal Outcomes in Clinical Research. CRC Press. New York.
Jung, S.H. and Ahn, C. 2004. K-Sample Test and Sample Size Calculation for Comparing Slopes in Data with Repeated Measurements. Biometrical Journal. Vol 46(5). Pages 554-564.


**Report Definitions**
Power is the probability of rejecting a false null hypothesis.
N is the total number of subjects in the study.
Group Alloc Prop Set ri gives the name of the set containing group allocation proportions.
Meas Time M is the number of time points at which each subject is scheduled to be measured.
Time Set gives the name of the set containing the time proportions. The time values represent the proportion of the total study time that has elapsed just before the measurement.
Grp Slope Set βi gives the name of the set containing the slopes for each group.
σ is the standard deviation of a response.
ρ is the base correlation between two responses on the same subject. It may be transformed based on the correlation pattern.
First Row of Corr Matrix presents the top row of the correlation matrix.
Alpha is the significance level of the test. The probability of rejecting the null hypothesis when the null hypothesis is true.
Prop Miss gives the proportion of missing values used for all time values.

## 11.3 Statistical Analysis Method

The primary analysis of the RCT will follow the intention-to-treat (ITT) principle. Missing values are assumed to occur completely at random (MCAR), data for missing outcomes are imputed using the last observation carried forward (LOCF). The analyses of the secondary endpoints will follow the same lines as the analysis of the primary endpoint, where appropriate.

Demographics and baseline characteristics such as age, sex, tinnitus site, baseline THI, baseline HADS are summarized by 3 university teaching hospitals. Categorical variables are presented using frequencies and percentages. Continuous variables are expressed as means ± standard deviation in normal distribution; otherwise, we will use medians and interquartile ranges (IQR). The normality of distributions is assessed graphically and by using the Shapiro–Wilk test. Differences in characteristics among the groups are tested with chi-square and fisher test for categorical variables; one-way ANOVA is used for normally distributed continuous variables, and the Mann-Whitney U test is used for non-normal continuous variables. Means and 95% confidence intervals (CIs) are calculated

for THI, HADS-A, HADS-D, AIS, VAS scores at baseline, 1-, 2-, 3-, 6-, and 9-months post-treatment.

The effect size will be determined using Cohen’s d and Hedge's g which is indicative of the effectiveness of treatment and helps to assess whether a statistically significant difference is of practical concern. Cohen’s d and Hedge's g values are classified as trivial (0-0.2), small (0.2-0.5), medium (0.5-0.8), large (0.8-1.0), and very large (>1.0).

Generalized estimating equations (GEE) under generalized linear model classification are used to analyze the differences of the four groups of outcome indicators over time, and demographic data, disease data and psychoacoustic indicators of tinnitus at baseline are controlled as covariables. All shedding cases will be included in the intention to treat (ITT) analysis, and the missing value is filled by the method of carrying forward the last observation data before the loss of follow-up. A simultaneous "Per-protocol sets" analysis is also performed, and the results of the ITT and PPS analyses will be compared. Two-side P-values below 0.05 are considered significant. Statistical analyses are performed using R software (version 4.2.3).

## 11.4 Sensitivity Analyses

The following sensitivity analyses are pre-specified: 1) handling missing data using the last observation carried forward (LOCF) approach, and 2) developing a multivariable generalized estimating equations model to adjust for potential confounders, including age, gender, tinnitus course, tinnitus site, tinnitus tone and tinnitus state.

## 11.5 Plans for Interim Analysis

No interim analyses are planned for this study.

## 11.6 Data Confidentiality

Patient information is kept anonymous and confidential throughout the analysis process. By separating the intervention and analysis staff, independent investigators in our hospital will perform all follow-up evaluations blinded to the group allocation.

## 11.7 Safety Assessment

Previous studies have shown few advert events of sound therapy. Major possible side effect might be hearing damage caused by the excessive volumes of the sound. In this study, we set a strict maximum playback intensity limit to make sure all therapeutic sounds are absolutely less than the discomfort loudness threshold. Any unexpected

events (such as significant hearing loss, dizziness, etc.) that occur in patients during the study follow-up period will be considered adverse events and need to be properly handled. The severity, occurrence time, duration, treatment measures and course of symptoms will be recorded in detail in the case report form (CRF) and reported to the project leader of each center.

Adverse events will be coded using the MedDRA system, as prepared by the ICH, and subsequently tabulated and described. Comparisons of adverse event incidence across groups will be conducted using the $\chi^2$ test or Fisher's exact test as appropriate. Additionally, a detailed statistical description will be provided for normal or abnormal changes in laboratory test results before and after administration of the sound therapy in each group, along with any potential relationship to the sound therapy. In theory, no adverse reactions are expected in this study; however, should any arise, the above methods will be applied to assess them.

## 11.8 Reporting

Throughout the entire duration of the trial, AEs will be systematically collected and documented in our study. In order to assess signals in the intended patient population:

Cases of noncompliance, protocol violations, participants lost to follow-up, and any other reasons why participants drop out of the study will be assessed for presence of AEs.

# 12. Study Management

Prior to study initiation, the study protocol, protocol amendments, and other relevant documents (such as CRFs) have been reviewed and discussed by the research team and submitted by the principal investigator to the Ethics Committee for ethical review and approval. Any further amendments to the protocol will be embodied in the revised version of protocol which will be signed by the principal investigator and reviewed and approved by the Ethics Committee. The revised approved version approval will be attached to this protocol.

Doctors participating in this study will be trained and supervised by senior professionals.

## 12.1 Trial management and quality control

A data monitoring committee (DMC) has been established to store, monitor, and check the authenticity, security, and integrity of the database. All members in the DMC are independent of the study sponsors and declare no competing interests. The DMC will

periodically review the accumulated data and communicate the problems of its deliberations to the study team if necessary.

A semi-annual audit will be conducted to protect the integrity of all collected data as part of this trial. The principal investigator will ensure that data collection is conducted and reported according to the protocol. There will be semi-annual auditing trials to ensure the protocol is followed, there are no issues with the informed consent procedure, and record keeping is accurate.

## 12.2 Ethics and disseminations

This protocol and the template informed consent forms have been reviewed and approved by the institutional review board (IRB) and the Ethical Committee of Eye & ENT Hospital Affiliated to Fudan University (Reference Number: 2017048-1 and 2017048-2). The research team will make safety and progress reports to the IRB at least annually and within 3 months of study completion. Patients and the public are not involved in the design of this study. The study results will be informed to the public via peer-reviewed journals or academic conferences. The full protocol and dataset will be available from the corresponding author upon reasonable request.

## 12.3 Pre-study Training

To ensure that our multicenter study adhered to strict quality standards, the clinical study protocol will be jointly discussed and formulated by the key persons responsible for the study prior to the official study start date. All medical staff participating in the study will undergo appropriate training. The study project manager will be responsible for arranging the training of study investigators by experts before the clinical study initiation; the training will be mainly related to the study protocol, operating processes, instructions for form completion, and the organization of Q&A session on these topics.

The study project manager and the investigators will take their respective responsibilities, strictly following the clinical study protocol and standard operating procedures, to ensure the implementation of stringent quality control and assurance systems throughout the clinical study.

## 12.4 Quality Control for Data Collection

The participating hospitals will conduct the study according to the clinical study protocol. All the results and abnormal findings observed in the clinical study will be carefully verified and recorded in time to ensure the reliability of the data. The recording and

transfer of clinical data will be supervised or checked by dedicated staff to ensure the scientific accuracy of the data. The conclusions of the clinical study will only be drawn from the raw data.

The study team will ensure that they complete the CRFs accurately and in a timely manner. All study-related data will be centrally managed and analyzed.

Standardized statistical analyses will be performed to summarize and analyze the clinical study results.

# 13. Patient Privacy and Data Protection

The patients' medical records (e.g., medical charts and test reports) will be kept in their entirety within the hospital. Doctors participating in the study, the ethics committees, and the food and drug administrations will be allowed access to the patients' medical records. Any published outputs arising from this study will not disclose the patients' personal identity. We will make every effort to protect the privacy of the patients in relation to their personal medical data to the extent permitted by law.

Other future studies will be allowed to reuse the patients' medical records, provided that the patients' personal identity will not be disclosed.

# 14. Participating Sites in the Multicenter Study

The study is planned to involve multiple study sites, primarily the tertiary hospitals of Shanghai city. Specifically, the planned participating sites are as follows: Eye & ENT Hospital Affiliated to Fudan University (EENT Hospital), Yueyang Hospital of Integrative Chinese & Western Medicine Affiliated to Shanghai University of Traditional Chinese Medicine (Yueyang Hospital), and Zhongshan Hospital Affiliated to Fudan University (Zhongshan Hospital). The participating sites may be updated to ensure the enrollment of sufficient number of patients.

The study has been reviewed and approved by the ethics committee of the lead participating site (EENT Hospital).

# 15. Trial status

Participant recruitment for this trial will be scheduled to commence in April 2021, with an anticipated completion in April 2023.

# 16. Reference


1. Langguth, B., et al., *Tinnitus: causes and clinical management.* Lancet Neurol, 2013. **12**(9): p. 920-930.
2. Bauer, C.A., *Tinnitus.* The New England journal of medicine, 2018. **378**(13): p. 1224-1231.
3. McCormack, A., et al., *A systematic review of the reporting of tinnitus prevalence and severity.* Hear Res, 2016. **337**: p. 70-9.
4. Rosing, S.N., et al., *Prevalence of tinnitus and hyperacusis in children and adolescents: a systematic review.* BMJ Open, 2016. **6**(6): p. e010596.
5. Durai, M. and G. Searchfield, *Anxiety and depression, personality traits relevant to tinnitus: A scoping review.* Int J Audiol, 2016. **55**(11): p. 605-15.
6. Krog, N.H., B. Engdahl, and K. Tambs, *The association between tinnitus and mental health in a general population sample: results from the HUNT Study.* J Psychosom Res, 2010. **69**(3): p. 289-98.
7. Xu, Y., et al., *Association between sleep quality and psychiatric disorders in patients with subjective tinnitus in China.* European archives of oto-rhino-laryngology : official journal of the European Federation of Oto-Rhino-Laryngological Societies (EUFOS) : affiliated with the German Society for Oto-Rhino-Laryngology - Head and Neck Surgery, 2016. **273**(10): p. 3063-3072.
8. Michiels, S., et al., *Diagnostic Criteria for Somatosensory Tinnitus: A Delphi Process and Face-to-Face Meeting to Establish Consensus.* Trends Hear, 2018. **22**: p. 2331216518796403.
9. Shore, S.E., L.E. Roberts, and B. Langguth, *Maladaptive plasticity in tinnitus--triggers, mechanisms and treatment.* Nat Rev Neurol, 2016. **12**(3): p. 150-60.
10. De Ridder, D., et al., *Phantom percepts: tinnitus and pain as persisting aversive memory networks.* Proceedings of the National Academy of Sciences of the United States of America, 2011. **108**(20): p. 8075-8080.
11. Galazyuk, A.V., J.J. Wenstrup, and M.A. Hamid, *Tinnitus and underlying brain mechanisms.* Curr Opin Otolaryngol Head Neck Surg, 2012. **20**(5): p. 409-15.
12. Tunkel, D.E., et al., *Clinical practice guideline: tinnitus executive summary.* Otolaryngol Head Neck Surg, 2014. **151**(4): p. 533-41.
13. Lewis, S., et al., *Assessment and management of tinnitus: summary of NICE guidance.* BMJ, 2020. **368**: p. m976.
14. Wang, H., et al., *The state of the art of sound therapy for subjective tinnitus in adults.* Ther Adv Chronic Dis, 2020. **11**: p. 2040622320956426.
15. Chemali, Z., R. Nehme, and G. Fricchione, *Sensory neurologic disorders: Tinnitus.* Handb Clin Neurol, 2019. **165**: p. 365-381.
16. Bauer, C.A. and T.J. Brozoski, *Effect of tinnitus retraining therapy on the loudness and annoyance of tinnitus: a controlled trial.* Ear Hear, 2011. **32**(2): p. 145-55.
17. Forti, S., et al., *Are results of tinnitus retraining therapy maintained over time? 18-month follow-up after completion of therapy.* Audiol Neurootol, 2009. **14**(5): p. 286-9.
18. Hobson, J., E. Chisholm, and A. El Refaie, *Sound therapy (masking) in the management of tinnitus in adults.* Cochrane Database Syst Rev, 2012. **11**: p. CD006371.

19. Vernon, J., *Attemps to relieve tinnitus.* Journal of the American Audiology Society, 1977. **2**(4): p. 124-131.
20. Henry, J.A., et al., *Multisite Randomized Controlled Trial to Compare Two Methods of Tinnitus Intervention to Two Control Conditions.* Ear Hear, 2016. **37**(6): p. e346-e359.
21. Tinnitus Retraining Therapy Trial Research, G., R.W. Scherer, and C. Formby, *Effect of Tinnitus Retraining Therapy vs Standard of Care on Tinnitus-Related Quality of Life: A Randomized Clinical Trial.* JAMA Otolaryngol Head Neck Surg, 2019. **145**(7): p. 597-608.
22. Tang, D., H. Li, and L. Chen, *Advances in Understanding, Diagnosis, and Treatment of Tinnitus.* Adv Exp Med Biol, 2019. **1130**: p. 109-128.
23. Krick, C.M. and H. Argstatter, *Neural correlates of the Heidelberg Music Therapy: indicators for the regeneration of auditory cortex in tinnitus patients?* Neural Regen Res, 2015. **10**(9): p. 1373-5.
24. Okamoto, H., et al., *Listening to tailor-made notched music reduces tinnitus loudness and tinnitus-related auditory cortex activity.* Proceedings of the National Academy of Sciences of the United States of America, 2010. **107**(3): p. 1207-1210.
25. Wazen, J.J., et al., *Evaluation of a customized acoustical stimulus system in the treatment of chronic tinnitus.* Otol Neurotol, 2011. **32**(4): p. 710-6.
26. Li, S.A., L. Bao, and M. Chrostowski, *Investigating the Effects of a Personalized, Spectrally Altered Music-Based Sound Therapy on Treating Tinnitus: A Blinded, Randomized Controlled Trial.* Audiol Neurootol, 2016. **21**(5): p. 296-304.
27. Jang, D.W., E. Johnson, and S.S. Chandrasekhar, *Neuromonics Tinnitus Treatment: preliminary experience in a private practice setting.* Laryngoscope, 2010. **120 Suppl 4**: p. S208.
28. Hanley, P.J. and P.B. Davis, *Treatment of tinnitus with a customized, dynamic acoustic neural stimulus: underlying principles and clinical efficacy.* Trends Amplif, 2008. **12**(3): p. 210-22.
29. Stein, A., et al., *Inhibition-induced plasticity in tinnitus patients after repetitive exposure to tailor-made notched music.* Clin Neurophysiol, 2015. **126**(5): p. 1007-15.
30. Stein, A., et al., *Clinical trial on tonal tinnitus with tailor-made notched music training.* BMC Neurol, 2016. **16**: p. 38.
31. Teismann, H., H. Okamoto, and C. Pantev, *Short and intense tailor-made notched music training against tinnitus: the tinnitus frequency matters.* PLoS One, 2011. **6**(9): p. e24685.
32. Tang, D., et al., *The clinical effects of modified tinnitus relieving sound (MTRS) for chronic tinnitus: protocol for a randomized controlled trial.* Trials, 2023. **24**(1).
33. Organization, W.H., *World report on hearing*. 2021: World Health Organization.
34. Jerger, J., *Clinical experience with impedance audiometry.* Arch Otolaryngol, 1970. **92**(4): p. 311-24.
35. Lidén, G., *The scope and application of current audiometric tests.* J Laryngol Otol, 1969. **83**(6): p. 507-20.
36. Nondahl, D.M., et al., *Prevalence and 5-year incidence of tinnitus among older adults: the epidemiology of hearing loss study.* J Am Acad Audiol, 2002. **13**(6): p. 323-31.
37. Gopinath, B., et al., *Incidence, Persistence, and Progression of Tinnitus Symptoms in Older Adults: The Blue Mountains Hearing Study.* Ear and Hearing, 2010. **31**(3): p. 407-412.

38. Newman, C.W., S.A. Sandridge, and G.P. Jacobson, *Psychometric adequacy of the Tinnitus Handicap Inventory (THI) for evaluating treatment outcome*. J Am Acad Audiol, 1998. **9**(2): p. 153-60.

# 17. Appendix

The THI, HADS, AIS-8, VAS assessments (English Version), ICF, and CRF are presented as below:

## TINNITUS HANDICAP INVENTORY

Patient Name: ______________________________ Date: ____________

**INSTRUCTIONS:** The purpose of this questionnaire is to identify difficulties that you may be experiencing because of your tinnitus. Please answer every question. Please do not skip any questions.

| | | | |
|---|---|---|---|
| 1. Because of your tinnitus, is it difficult for you to concentrate? | Yes | Sometimes | No |
| 2. Does the loudness of your tinnitus make it difficult for you to hear people? | Yes | Sometimes | No |
| 3. Does your tinnitus make you angry? | Yes | Sometimes | No |
| 4. Does your tinnitus make you feel confused? | Yes | Sometimes | No |
| 5. Because of your tinnitus, do you feel desperate? | Yes | Sometimes | No |
| 6. Do you complain a great deal about your tinnitus? | Yes | Sometimes | No |
| 7. Because of your tinnitus, do you have trouble falling to sleep at night? | Yes | Sometimes | No |
| 8. Do you feel as though you cannot escape your tinnitus? | Yes | Sometimes | No |
| 9. Does your tinnitus interfere with your ability to enjoy your social activities (such as going out to dinner, to the movies)? | Yes | Sometimes | No |
| 10. Because of your tinnitus, do you feel frustrated? | Yes | Sometimes | No |
| 11. Because of your tinnitus, do you feel that you have a terrible disease? | Yes | Sometimes | No |
| 12. Does your tinnitus make it difficult for you to enjoy life? | Yes | Sometimes | No |
| 13. Does your tinnitus interfere with your job or household responsibilities? | Yes | Sometimes | No |
| 14. Because of your tinnitus, do you find that you are often irritable? | Yes | Sometimes | No |
| 15. Because of your tinnitus, is it difficult for you to read? | Yes | Sometimes | No |
| 16. Does your tinnitus make you upset? | Yes | Sometimes | No |
| 17. Do you feel that your tinnitus problem has placed stress on your relationships with members of your family and friends? | Yes | Sometimes | No |
| 18. Do you find it difficult to focus your attention away from your tinnitus and on other things? | Yes | Sometimes | No |
| 19. Do you feel that you have no control over your tinnitus? | Yes | Sometimes | No |
| 20. Because of your tinnitus, do you often feel tired? | Yes | Sometimes | No |
| 21. Because of your tinnitus, do you feel depressed? | Yes | Sometimes | No |
| 22. Does your tinnitus make you feel anxious? | Yes | Sometimes | No |
| 23. Do you feel that you can no longer cope with your tinnitus? | Yes | Sometimes | No |
| 24. Does your tinnitus get worse when you are under stress? | Yes | Sometimes | No |
| 25. Does your tinnitus make you feel insecure? | Yes | Sometimes | No |

| | | | | | | | |
|---|---|---|---|---|---|---|---|
| **Total Per Column** | | | | | | | |
| | x4 | | x2 | | x0 | | |
| **Total Score** | | + | | + | | = | |

## Hospital Anxiety and Depression Scale (HADS)

**Tick the box beside the reply that is closest to how you have been feeling in the past week.**
**Don't take too long over you replies: your immediate is best.**

| D | A | | D | A | |
|---|---|---|---|---|---|
| | | **I feel tense or 'wound up':** | | | **I feel as if I am slowed down:** |
| | 3 | Most of the time | 3 | | Nearly all the time |
| | 2 | A lot of the time | 2 | | Very often |
| | 1 | From time to time, occasionally | 1 | | Sometimes |
| | 0 | Not at all | 0 | | Not at all |
| | | | | | |
| | | **I still enjoy the things I used to enjoy:** | | | **I get a sort of frightened feeling like 'butterflies' in the stomach:** |
| 0 | | Definitely as much | | 0 | Not at all |
| 1 | | Not quite so much | | 1 | Occasionally |
| 2 | | Only a little | | 2 | Quite Often |
| 3 | | Hardly at all | | 3 | Very Often |
| | | | | | |
| | | **I get a sort of frightened feeling as if something awful is about to happen:** | | | **I have lost interest in my appearance:** |
| | 3 | Very definitely and quite badly | 3 | | Definitely |
| | 2 | Yes, but not too badly | 2 | | I don't take as much care as I should |
| | 1 | A little, but it doesn't worry me | 1 | | I may not take quite as much care |
| | 0 | Not at all | 0 | | I take just as much care as ever |
| | | | | | |
| | | **I can laugh and see the funny side of things:** | | | **I feel restless as I have to be on the move:** |
| 0 | | As much as I always could | | 3 | Very much indeed |
| 1 | | Not quite so much now | | 2 | Quite a lot |
| 2 | | Definitely not so much now | | 1 | Not very much |
| 3 | | Not at all | | 0 | Not at all |
| | | **Worrying thoughts go through my mind:** | | | **I look forward with enjoyment to things:** |
| | 3 | A great deal of the time | 0 | | As much as I ever did |
| | 2 | A lot of the time | 1 | | Rather less than I used to |
| | 1 | From time to time, but not too often | 2 | | Definitely less than I used to |
| | 0 | Only occasionally | 3 | | Hardly at all |
| | | | | | |
| | | **I feel cheerful:** | | | **I get sudden feelings of panic:** |
| 3 | | Not at all | | 3 | Very often indeed |
| 2 | | Not often | | 2 | Quite often |
| 1 | | Sometimes | | 1 | Not very often |
| 0 | | Most of the time | | 0 | Not at all |
| | | | | | |
| | | **I can sit at ease and feel relaxed:** | | | **I can enjoy a good book or radio or TV program:** |
| | 0 | Definitely | 0 | | Often |
| | 1 | Usually | 1 | | Sometimes |
| | 2 | Not Often | 2 | | Not often |
| | 3 | Not at all | 3 | | Very seldom |

Please check you have answered all the questions

Scoring:
Total score: Depression (D) ___________ Anxiety (A) ______________
0-7 = Normal
8-10 = Borderline abnormal (borderline case)
11-21 = Abnormal (case)

## ATHENS INSOMNIA SCALE

*This scale is intended to record your own assessment of any sleep difficulty you might have experienced. Please, check (by circling the appropriate number) the items below to indicate your estimate of any difficulty, provided that it occurred at least three times per week during the last month.*

1. SLEEP INDUCTION *(time it takes you to fall asleep after turning-off the lights)*

| **0** | **1** | **2** | **3** |
|---|---|---|---|
| No problem | Slightly delayed | Markedly delayed | Very delayed or did not sleep at all |

2. AWAKENINGS DURING THE NIGHT

| **0** | **1** | **2** | **3** |
|---|---|---|---|
| No problem | Minor problem | Considerable problem | Serious problem or did not sleep at all |

3. FINAL AWAKENING EARLIER THAN DESIRED

| **0** | **1** | **2** | **3** |
|---|---|---|---|
| Not earlier | A little earlier | Markedly earlier | Much earlier or did not sleep at all |

4. TOTAL SLEEP DURATION

| **0** | **1** | **2** | **3** |
|---|---|---|---|
| Sufficient | Slightly insufficient | Markedly insufficient | Very insufficient or did not sleep at all |

5. OVERALL QUALITY OF SLEEP *(no matter how long you slept)*

| **0** | **1** | **2** | **3** |
|---|---|---|---|
| Satisfactory | Slightly unsatisfactory | Markedly unsatisfactory | Very unsatisfactory or did not sleep at all |

6. SENSE OF WELL-BEING DURING THE DAY

| **0** | **1** | **2** | **3** |
|---|---|---|---|
| Normal | Slightly decreased | Markedly decreased | Very decreased |

7. FUNCTIONING (PHYSICAL AND MENTAL) DURING THE DAY

| **0** | **1** | **2** | **3** |
|---|---|---|---|
| Normal | Slightly decreased | Markedly decreased | Very decreased |

8. SLEEPINESS DURING THE DAY

| **0** | **1** | **2** | **3** |
|---|---|---|---|
| None | Mild | Considerable | Intense |

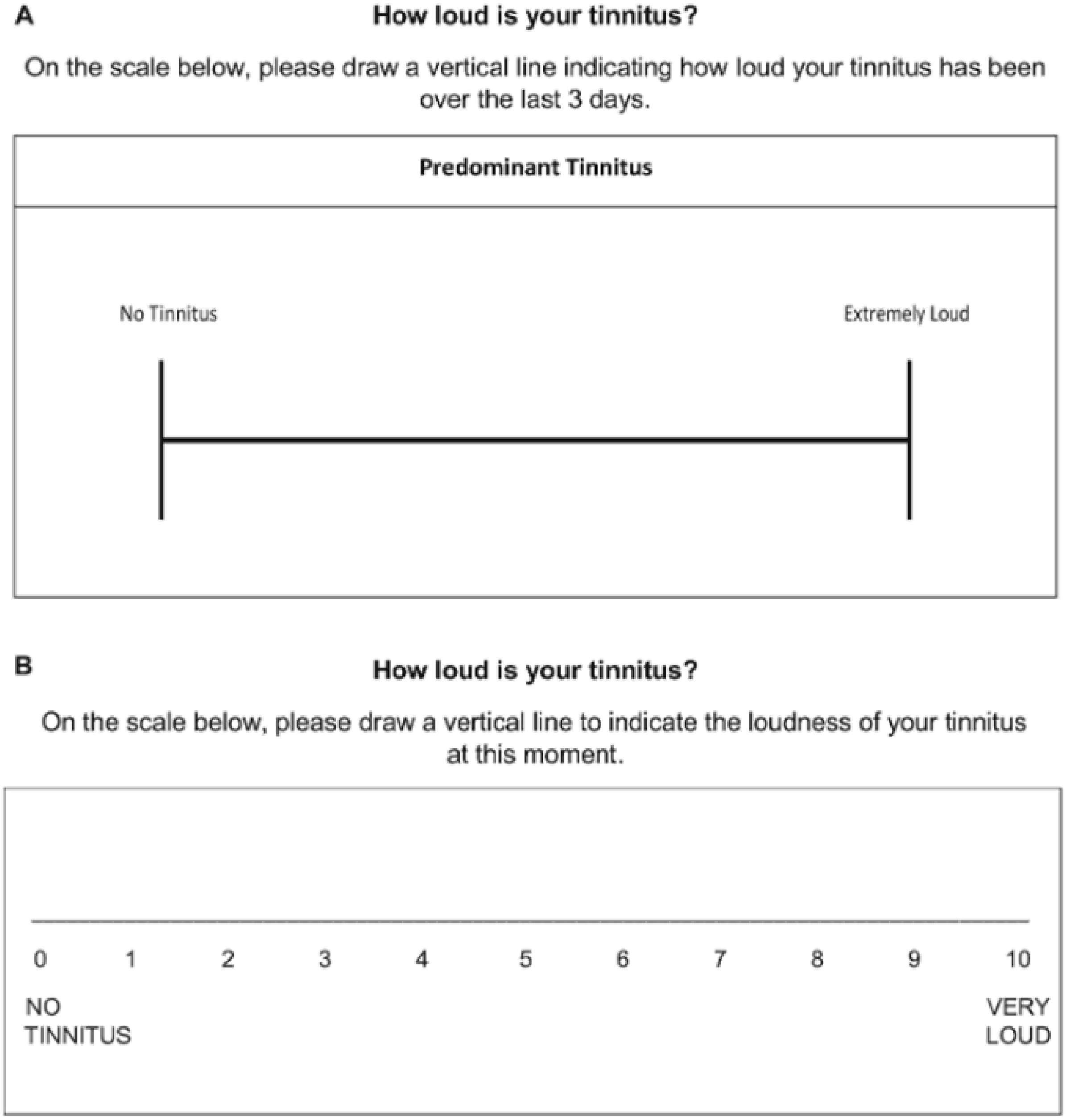


(**a** and **b**) An example of a Visual Analogue Scale is provided in (**a**) and a Numeric Rating Scale in (**b**)

## Informed Consent Form

Dear Sir/Madam:

We invite you to participate in a clinical research study on tinnitus sound therapy. Before you decide whether to join this study, please carefully read this informed consent form and make a well-considered decision. You may ask your research doctor or research staff any questions you have about the study until you fully understand. You may also discuss your decision with your family and friends. If you are currently participating in another study, please inform your research doctor or research staff. Below is a summary of the study:

1. Background of the Study:

(1) Tinnitus has a high prevalence worldwide, affecting approximately 10–25% of adults. It impacts patients' sleep, mood, work, and family life. However, effective cures for tinnitus remain a recognized challenge in the medical field. This study aims to provide different sound interventions based on tinnitus assessment results, evaluate the effectiveness of sound therapy for tinnitus, and further clarify on which sound therapy strategy is more effective and which characteristics of tinnitus patients respond best.

(2) This study has been approved by the Ethics Committee of the Eye & ENT Hospital Affiliated to Fudan University. It will adhere to the international principles of the Declaration of Helsinki and Chinese national laws and regulations, complying with medical ethics.

2. Study Design and Process:

This study does not involve the collection of tissue, blood, or other samples. Specific steps and procedures are as follows:

(1) Collect general medical history, conduct tinnitus inquiries, audiological testing, and CT/MRI (if necessary) to confirm the diagnosis of tinnitus and classify its severity.

(2) Conduct tinnitus matching tests, including Pitch Matching (PM) and Loudness Matching (LM).

(3) The four sound interventions are unmodified music (UM), combination of UM and narrowband noise centered at tinnitus pitch (UM + NBN), high frequency- enhanced music (HFEM), and digital frequency customized relieving sound (DFCRS),

respectively. Participants will listen to the therapeutic sound for a total of at least 2 hours per day for 9 months, with the volume set to partially mask tinnitus while remaining comfortable.

(4) Assess participants at the beginning of treatment and at 1, 2, 3, 6, and 9 months into the treatment. The assessments include the Tinnitus Handicap Inventory (THI), Hospital Anxiety and Depression Scale (HADS), Athens Insomnia Scale (AIS), and Visual Analog Scale (VAS).

(5) Confidentiality of Personal Privacy: Your medical records will be securely stored and accessible only to research staff. When required, government authorities or ethics committee members may review your personal data in compliance with regulations. Study results will be published as aggregated statistical data without any identifiable participant information.

3. Inclusion and Exclusion Criteria:

Inclusion Criteria:

(1) Adults aged 18–80, regardless of gender.
(2) Subjective tinnitus lasting for at least three months.
(3) Hearing loss with an average pure-tone threshold ≤ 55 dB HL (0.5, 1, 2 kHz) in the worse-hearing ear.
(4) Ability to communicate in Mandarin independently or with assistance.

Exclusion Criteria:

(1) Vascular pulsatile tinnitus or objective tinnitus.
(2) Major health issues that prevent participation or follow-up compliance.
(3) Tinnitus caused by infection, tumor, trauma, neurogenic factors, otosclerosis, Ménière's disease, or sudden deafness.
(4) Severe hyperacusis or severe anxiety/depression.
(5) Participation in other studies potentially affecting tinnitus.
(6) Determination by the researcher that the participant is unsuitable for the study.

4. Possible Risks and Benefits:

(1) Possible Risks: This is a non-invasive study that does not interfere with your diagnosis or treatment and will not cause harm to your physical, psychological, or social well-being. The study process is supervised by the Ethics Committee of the Eye & ENT Hospital. If you have any questions during the study, you can consult the research doctor.

(2) Possible Benefits: Participation will not incur additional costs. The study process and results may help provide effective interventions for your tinnitus and contribute valuable data for advancing tinnitus research in China. There will be no monetary compensation for your participation, but we sincerely thank you for contributing to scientific research and medical advancement.

5. Voluntary Participation:

Your participation in this study is entirely voluntary. You may withdraw from the study at any time without providing a reason. Withdrawal will not affect your relationship with the medical staff or your future medical care.

6. Contact Information:

If you have any questions about this study, please contact Dr. Tang Dongmei at the Eye & ENT Hospital Affiliated to Fudan University. Phone: 13023299189. If you have concerns regarding your rights as a participant, face any difficulties, or wish to provide feedback or suggestions related to the study, you may contact the research doctor.

**Informed Consent Signature Section**

I have read the above information and understand the purpose of the study and its potential benefits. I have received satisfactory answers to all my questions regarding the study procedures and content. I voluntarily sign this consent form and agree to participate in the study.

Participant’s Signature: Date of Signature:

Participant’s Contact Number:

I have read and explained this informed consent form to the participant and answered all their questions. The participant has understood the information and agreed to participate in this scientific research.

Researcher’s Signature: Date of Signature:

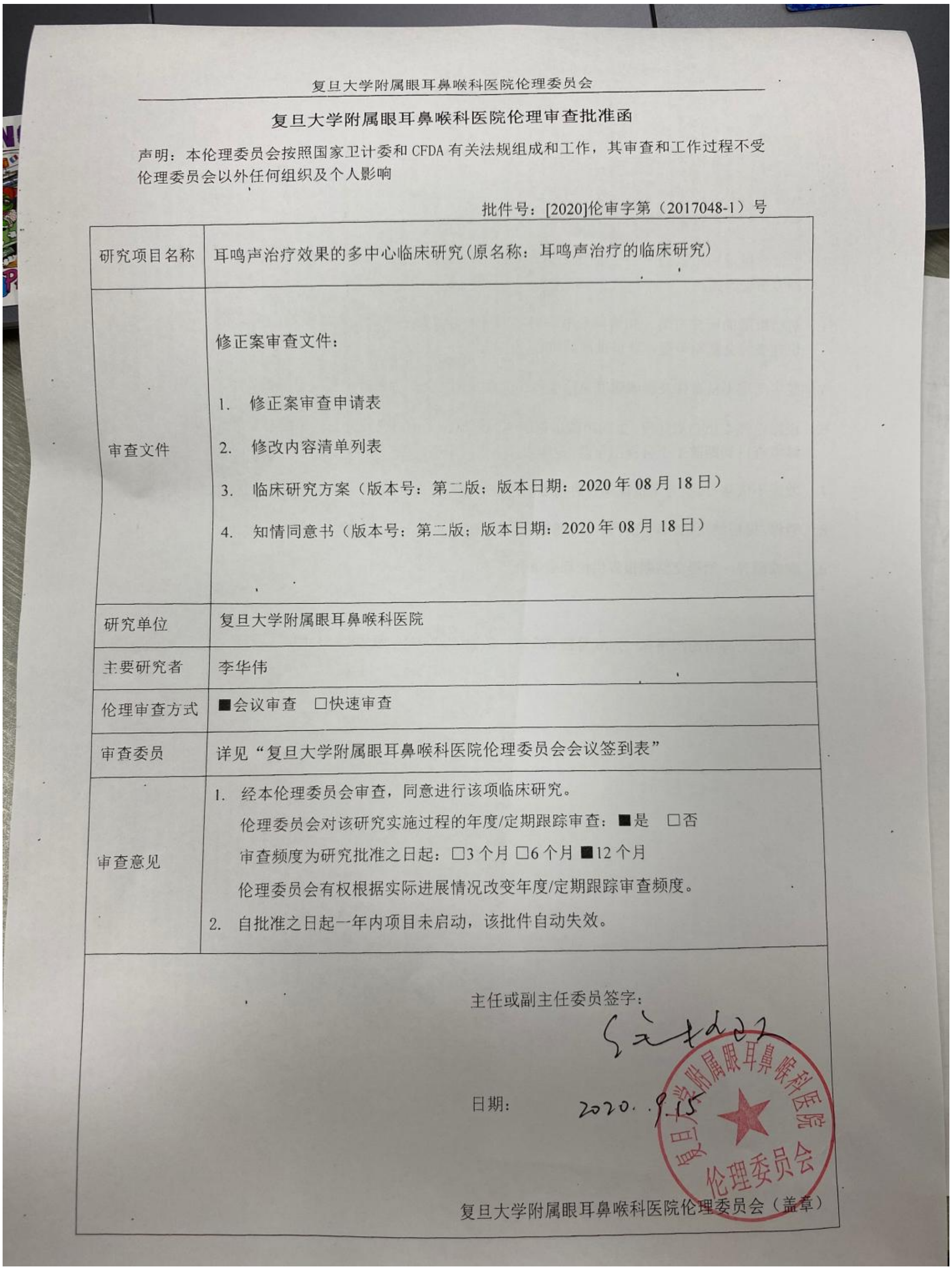

复旦大学附属眼耳鼻喉科医院伦理委员会

**复旦大学附属眼耳鼻喉科医院伦理审查批准函**

声明：本伦理委员会按照国家卫计委和 CFDA 有关法规组成和工作，其审查和工作过程不受伦理委员会以外任何组织及个人影响

批件号：[2020]伦审字第（2017048-1）号

| | |
|---|---|
| 研究项目名称 | 耳鸣声治疗效果的多中心临床研究（原名称：耳鸣声治疗的临床研究） |
| 审查文件 | 修正案审查文件：<br>1. 修正案审查申请表<br>2. 修改内容清单列表<br>3. 临床研究方案（版本号：第二版；版本日期：2020 年 08 月 18 日）<br>4. 知情同意书（版本号：第二版；版本日期：2020 年 08 月 18 日） |
| 研究单位 | 复旦大学附属眼耳鼻喉科医院 |
| 主要研究者 | 李华伟 |
| 伦理审查方式 | ■会议审查 □快速审查 |
| 审查委员 | 详见“复旦大学附属眼耳鼻喉科医院伦理委员会会议签到表” |
| 审查意见 | 1. 经本伦理委员会审查，同意进行该项临床研究。<br>伦理委员会对该研究实施过程的年度/定期跟踪审查：■是 □否<br>审查频度为研究批准之日起：□3 个月 □6 个月 ■12 个月<br>伦理委员会有权根据实际进展情况改变年度/定期跟踪审查频度。<br>2. 自批准之日起一年内项目未启动，该批件自动失效。 |

主任或副主任委员签字：[illegible]

日期：2020. 9.15

复旦大学附属眼耳鼻喉科医院伦理委员会

复旦大学附属眼耳鼻喉科医院伦理委员会（盖章）

# Case Report Form

**Subject's Name** ____________________

**ID Number** ____________________

**Research Institution Name：** ____________________

**Researcher's Signature：** ____________________

Revision date：2020-08-19

**General information** **Date of signing of informed consent：**

| | | | |
|---|---|---|---|
| Gender | □ Male □ Female | Age | |_|_| years |

▪ Tinnitus Site：

☐ Unilateral (Left Ear / Right Ear)

☐ Bilateral (Both Ears Equally / Louder in Left Ear / Louder in Right Ear)

☐ Central (Perceived in the Head)

▪ Duration of Tinnitus:_______months

▪ Nature of Tinnitus Sound:

☐ High-Pitched (e.g., Cicada Noise / Whistling / Chirping / Hissing)

☐ Low-Pitched (e.g., Buzzing / Humming)

☐ Noise (Mixed Sounds)

▪ Tinnitus State:

☐ Intermittent

☐ Continuous

*Remarks (Any Changes in Tinnitus):* __________

▪ Factors Associated with Tinnitus:

☐ Noise Exposure ☐ Sudden Hearing Loss ☐ Presbycusis (Age-Related Hearing Loss)

☐ Trauma ☐ Ototoxic Drugs ☐ Cervical Spondylosis ☐ Ménière's Disease

☐ Acoustic Neuroma ☐ Others: __________

▪ Systemic Factors:

☐ Hypertension ☐ Diabetes ☐ Hyperthyroidism or Hypothyroidism ☐ Lacunar Infarction

▪ Treatment History (Yes/No):

☐ If Sound Therapy: Type of Sound Therapy _______ Duration of Treatment _______

*Effect:* Tinnitus Disappeared / Improved / Worsened / No Effect

☐ If Medication: Name of Medication _______ Duration of Treatment _______

*Effect:* Tinnitus Disappeared / Improved / Worsened / No Effect

| |
|---|
| ▪ Severity Assessment (Multiple Selections Allowed):<br>☐ Annoying or Irritating<br>☐ Negatively Impacts Life (e.g., Insomnia, Anxiety, Depression)<br>☐ Affects Work, Family Life, or Household Chores |
| Any Previous Medical History or Comorbidities?<br>☐ No ☐ Yes (If Yes, Please Fill Out the Table Below): |

| Disease Diagnosis | Generic Name of Medication | Daily Dose | Dosage Method | Start-End Date (Mark "×" for Continuing) |
|---|---|---|---|---|
| | | | | |_|_|_|_|/|_|_|/|_|_|to|_|_|_|_|/|_|_|/|_|_|/□ |
| | | | | |_|_|_|_|/|_|_|/|_|_|to|_|_|_|_|/|_|_|/|_|_|/□ |
| | | | | |_|_|_|_|/|_|_|/|_|_|to|_|_|_|_|/|_|_|/|_|_|/□ |
| | | | | |_|_|_|_|/|_|_|/|_|_|to|_|_|_|_|/|_|_|/|_|_|/□ |
| | | | | |_|_|_|_|/|_|_|/|_|_|to|_|_|_|_|/|_|_|/|_|_|/□ |

## Hearing Test Results：

**PTA**

| | 125Hz | 250Hz | 500Hz | 1000Hz | 2000Hz | 4000Hz | 8000Hz |
|---|---|---|---|---|---|---|---|
| Right BC | | | | | | | |
| Right AC | | | | | | | |
| Left BC | | | | | | | |
| Left AC | | | | | | | |

**Tympanometry：**

Left ear ☐A ☐B ☐C ☐As ☐Ad

Right ear ☐A ☐B ☐C ☐As ☐Ad

**Other Hearing Tests (if applicable, please record):**

Stapedius Reflex:

DPOAE：

ASSR：

ABR：

**Tinnitus matching tests：**

| | Left ear | Right ear |
|---|---|---|
| Tinnitus site 1 | Hz, dB HL, dB SL | Hz, dB HL, dB SL |
| Tinnitus site 2 | Hz, dB HL, dB SL | Hz, dB HL, dB SL |

**Imaging Tests (if applicable, please record)**:

CT：

MRI：

## Inclusion Criteria

| | | |
|---|---|---|
| ✧ Adults aged 18–80, regardless of gender； | □Yes | □No |
| ✧ Subjective tinnitus； | □Yes | □No |
| ✧ Chronic tinnitus lasting for at least three months； | □Yes | □No |
| ✧ Ability to communicate in Mandarin independently or with assistance； | □Yes | □No |
| ✧ Hearing loss with an average pure-tone threshold ≤ 55 dB HL (0.5, 1, 2 kHz) in the worse-hearing ear； | □Yes | □No |
| ✧ The subject or their legal representative is able to understand the purpose of the study, voluntarily agrees to participate, can follow instructions to complete the experiment, and has signed the informed consent form. | □Yes | □No |

## Exclusion Criteria

| | | |
|---|---|---|
| ✧ Vascular pulsatile tinnitus or objective tinnitus; | □Yes | □No |
| ✧ Major health issues that prevent participation or follow-up compliance; | □Yes | □No |
| ✧ Tinnitus caused by infection, tumor, trauma, neurogenic factors, otosclerosis, Ménière’s disease, or sudden deafness; | □Yes | □No |
| ✧ Severe hyperacusis; | □Yes | □No |
| ✧ Diagnosed with severe anxiety/depression; | □Yes | □No |
| ✧ Participation in other studies potentially affecting tinnitus; | □Yes | □No |
| ✧ Determination by the researcher that the participant is unsuitable for the study. | □Yes | □No |

## Confirm Enrollment Date： 202|_|/|_|_|/|_|_|。

## First Assessment Record After Enrollment (Baseline)：

|_|_|_|_|Year|_|_|Month|_|_|Day

| Items | | Results |
|---|---|---|
| Primary Outcome | THI | Score： |
| Secondary Outcomes | HADS | Score：A (Anxiety) D (Depress) |
| | AIS | Score： |
| | VAS for tinnitus loudness | Score（0-10）： |
| | VAS for tinnitus influence | Score（0-10）： |

**Follow-up 1（1 month）：** |_|_|_|_|Year|_|_|Month|_|_|Day

| Items | | Results |
|---|---|---|
| Primary Outcome | THI | Score： |
| Secondary Outcomes | HADS | Score：A (Anxiety)　D (Depress) |
| | AIS | Score： |
| | VAS for tinnitus loudness | Score（0-10）： |
| | VAS for tinnitus influence | Score（0-10）： |

**Follow-up 2（2 months）：** |_|_|_|_|Year|_|_|Month|_|_|Day

| Items | | Results |
|---|---|---|
| Primary Outcome | THI | Score： |
| Secondary Outcomes | HADS | Score：A (Anxiety)　D (Depress) |
| | AIS | Score： |
| | VAS for tinnitus loudness | Score（0-10）： |
| | VAS for tinnitus influence | Score（0-10）： |

**Follow-up 3（3 months）：** |_|_|_|_|Year|_|_|Month|_|_|Day

| Items | | Results |
|---|---|---|
| Primary Outcome | THI | Score： |

| | | |
|---|---|---|
| Secondary Outcomes | HADS | Score：A (Anxiety)　D (Depress) |
| | AIS | Score： |
| | VAS for tinnitus loudness | Score（0-10）： |
| | VAS for tinnitus influence | Score（0-10）： |

**Follow-up 4（6 months）：** |_|_|_|_|Year|_|_|Month|_|_|Day

| Items | | Results |
|---|---|---|
| Primary Outcome | THI | Score： |
| Secondary Outcomes | HADS | Score：A (Anxiety)　D (Depress) |
| | AIS | Score： |
| | VAS for tinnitus loudness | Score（0-10）： |
| | VAS for tinnitus influence | Score（0-10）： |

**Follow-up 5（9 months）：** |_|_|_|_|Year|_|_|Month|_|_|Day

| Items | | Results |
|---|---|---|
| Primary Outcome | THI | Score： |
| Secondary Outcomes | HADS | Score：A (Anxiety)　D (Depress) |
| | AIS | Score： |
| | VAS for tinnitus loudness | Score（0-10）： |
| | VAS for tinnitus influence | Score（0-10）： |

**Extension Follow-up ：** |_|_|_|_|Year|_|_|Month|_|_|Day

| Items | | Results |
|---|---|---|
| Primary Outcome | THI | Score： |
| Secondary Outcomes | HADS | Score：A (Anxiety)    D (Depress) |
| | AIS | Score： |
| | VAS for tinnitus loudness | Score（0-10）： |
| | VAS for tinnitus influence | Score（0-10）： |

**Efficacy Evaluation at Study Completion：**

| | | |
|---|---|---|
| 1.Primary Outcome Improvement | □Yes | □No |
| 2.Secondary Outcome Improvement | □Yes | □No |

## Adverse Event Report Form

**Did an adverse event occur?**

| | | |
|---|---|---|
| Adverse Event Name | | |
| Clinical Diagnosis | | |
| Occurrence Date and Time | ||||| Year ||| Month ||| Day<br>||| Hour ||| Minute (24-hour format) | ||||| Year ||| Month ||| Day<br>||| Hour ||| Minute (24-hour format) |
| Severity of Adverse Event* | ☐ 1 Mild ☐ 2 Moderate ☐ 3 Severe | ☐ 1 Mild ☐ 2 Moderate ☐ 3 Severe |

| | | |
|---|---|---|
| Characteristics of the Adverse Event | ☐ Paroxysmal (Number of episodes: \|\|\|) ☐ Persistent<br>☐ Other: __________<br>☐ Paroxysmal (Number of episodes: \|\|\|) ☐ Persistent<br>☐ Other: __________ | ☐ Paroxysmal (Number of episodes: \|\|\|)<br>☐ Persistent<br>☐ Other: __________<br>☐ Paroxysmal (Number of episodes: \|\|\|)<br>☐ Persistent<br>☐ Other: __________ |
| Adverse Event Process<br>*(Including symptoms and signs)* | | |
| Management of Adverse Event<br>*(Including generic names of medications used, doses, methods of administration, and other treatments)* | ☐ None ☐ Yes (If yes, please record below): | ☐ None ☐ Yes (If yes, please record below): |
| **To be completed at the end of the adverse event or follow-up** | | |
| Follow-up Time | Time: \|\|\|\| Year \|\|\| Month \|\|\| Day \|\|\| Hour \|\|\| Minute (24-hour format) | Time: \|\|\|\| Year \|\|\| Month \|\|\| Day \|\|\| Hour \|\|\| Minute (24-hour format) |
| Outcome of the Adverse Event | ☐ 1 Resolved/Returned to Normal<br>☐ 2 Improved<br>☐ 3 Not Improved<br>☐ 4 Residual Effects (Specify: ____________) | ☐ 1 Resolved/Returned to Normal<br>☐ 2 Improved<br>☐ 3 Not Improved<br>☐ 4 Residual Effects (Specify: ____________) |
| Withdrawal from the Study | ☐ No ☐ Yes | ☐ No ☐ Yes |

**Note:** *Severity Grading:

1. Mild: Tolerable for the subject, requires no special treatment, and has no impact on the subject's health.
2. Moderate: Intolerable for the subject, directly affecting the subject's health.
3. Severe: Life-threatening, fatal, or disabling, requiring emergency treatment.*

### *Summary of Trial Completion*

| |
|---|
| Was this clinical trial completed?<br><br>**Was this clinical trial completed?**<br><br>☐ Yes ☐ No (If no, please complete the following):<br><br>**Date of Withdrawal from the Trial:** ||||| Year ||| Month ||| Day<br><br>**Primary Reason for Withdrawal:**<br><br>☐ 1 Lost to Follow-Up<br><br>☐ 2 Adverse Event<br><br>☐ 3 Subject Withdrew Voluntarily (Reason: ____________)<br><br>☐ 4 Investigator Decided to Withdraw the Subject (Reason: ____________)<br><br>☐ 5 Other: ____________ |
| Did the subject experience any adverse events during the trial? ☐ Yes ☐ No |
| **If adverse events occurred, were they all resolved?**<br><br>☐ Yes ☐ No<br><br>*If no, the subject should be monitored until recovery or return to pre-treatment levels.* |
| **Is this a qualified case?**<br><br>☐ Yes ☐ No (If no, please complete the following):<br><br>☐ 1 Did not meet inclusion criteria<br><br>☐ 2 Withdrew from the trial prematurely<br><br>☐ 3 Poor adherence |

# Case Report Form (CRF) Review Statement

As the principal investigator at this trial site, I hereby declare:

Upon review, all entries in this Case Report Form are truthful, complete, and accurate.

Principal Investigator's Signature：

Date：______Year____Month____Day

# The Ethics Committee of the Eye and ENT Hospital of Fudan University
# Ethical Approval Form

Declaration: This ethics committee works according to the National Health Commission (NHC) and National Medical Products Administration (NMPA) regulations. The review and work processes of the ethics committee are independent.

EC Ref No. [2017]:2017048

| | | | |
|---|---|---|---|
| **Review Date** | 2017-08-24 | **Review Address** | 83 Fenyang Road, Bld 3, Floor 6, No.1 meeting room |
| **Project Title** | **Clinical trials of sound therapy for subjective tinnitus** | | |
| **Documents for Review** | Initial review:<br>1. Ethical Approval Application Form<br>2. Clinical study protocol (Version 1.0)<br>3. Informed consent (Version 1.0; 2017-08-15)<br>4. Resume of the principal investigator (Version 1.0; 2017-08-15)<br>5. Case report form and questionnaires (Version 1.0; 2017-08-15)<br>6. References (Version 1.0; 2017-08-15) | | |
| **Affiliation of Research** | The Eye and ENT Hospital of Fudan University | | |
| **Principal Investigator** | Huawei Li | | |
| **Review Mode** | ■Conference review □Express review | | |
| **Review Members** | See the sign-in sheets for details. | | |
| **Review Comments** | 1. The committee reviewed the study protocol and formally APPROVED this study to proceed under the current protocol.<br>Comments and Suggestions by the Ethics Committee: □No ■Yes:<br>Given the high volume of research projects submitted by principal investigators at this conference, it is recommended to involve additional principal investigators in some of the projects to ensure effective management and oversight..<br>2. If the project is not initiated within one year from the approval date, this approval will automatically expire. | | |
| Signature of the Director: Xingtao Zhou<br>Date: 2017.8.31<br>The Ethics Committee of the Eye and ENT Hospital of Fudan University (stamped) | | | |

NOTES: PLEASE READ CAREFULLY

1. The research projects approved by this Ethics Committee involve biomedical research involving human subjects and must be carried out strictly following the latest approved research protocol and informed consent form, as well as in compliance with the relevant domestic regulatory guidelines.
2. Before implementing a project that involves the export of human genetic resources or requires specific approval by relevant authorities following national regulations, the project must be declared to and approved by the relevant authorities.
3. The ethics committees of other institutions may be able to use this approval for reference purposes. If there is a different opinion regarding the protocol review, please contact the ethics committee as soon as possible.
4. Before incorporating any changes into the approved research protocol or informed consent form, the Ethics Committee must be notified on time for re-examination and approval.
5. This Ethics Committee should be notified of any severe adverse events or unintended events affecting the risk-benefit ratio of the study promptly.
6. According to the Ethics Committee's opinion regarding the frequency of annual/periodic follow-up reviews, please apply one month before the annual/periodic follow-up review date is due, regardless of whether the study has begun.
7. If there is any non-compliance with the protocol or breach of law, it must be reported immediately to the Ethics Committee.
8. In the event of a suspension or early termination of a clinical study, please inform the Ethics Committee immediately.
9. Upon completing the study, a final report must be submitted to the Ethics Committee for review.

Address: Room 305, Building 10, No. 83 Fen Yang Road, Shanghai, 200031, China;
Telephone Number: +86-021-64377134

# 复旦大学附属眼耳鼻喉科医院伦理审查批准函

声明：本伦理委员会按照国家卫计委和 CFDA 有关法规组成和工作，其审查和工作过程不受伦理委员会以外任何组织及个人影响

批件号：[2017]伦审字第（2017048）号

| 会议时间 | 2017-08-24 | 会议地点 | 汾阳路 83 号 3 号楼 6 楼第 1 会议室 |
|---|---|---|---|
| 研究项目名称 | 耳鸣声治疗的临床研究 | | |
| 审查文件 | 1. 伦理审查申请表<br>2. 临床研究方案（版本号 Version1.0）<br>3. 受试者知情同意书（版本号：第一版，版本日期 2017 年 8 月 15 日）<br>4. 病例报告表<br>5. 参考文献<br>6. 主要研究者简历 | | |
| 研究单位 | 复旦大学附属眼耳鼻喉科医院 | | |
| 主要研究者 | 李华伟 | | |
| 伦理审查方式 | ■会议审查 □快速审查 | | |
| 审查委员 | 详见“复旦大学附属眼耳鼻喉科医院伦理委员会会议签到表” | | |
| 审查意见 | 1. 经本伦理委员会审查，同意进行该项临床研究。<br>意见和建议：□无■有：鉴于本次会议主要研究者申报的研究项目数量较多，建议为部分项目增加主要研究者。<br>2. 自批准之日起一年内项目未启动，该批件自动失效。 | | |

主任或副主任委员签字：[illegible]

日期：2017.8.31

复旦大学附属眼耳鼻喉科医院伦理委员会（盖章）

# The Ethics Committee of the Eye and ENT Hospital of Fudan University
# Ethical Approval Form

Declaration: This ethics committee works according to the National Health Commission (NHC) and National Medical Products Administration (NMPA) regulations. The review and work processes of the ethics committee are independent.

EC Ref No. [2020]:2017048-1

| | |
|---|---|
| **Project Title** | **A randomized multicenter controlled clinical trial on the efficacy of sound therapy for tinnitus management (Previous title: Clinical trials of sound therapy for subjective tinnitus)** |
| **Documents for Review** | Amendments review documents:<br>1. Amendment Review Application Form<br>2. List of modified content<br>3. Clinical study protocol (Version 2.0, 2020-8-18)<br>4. Informed consent (Version 2.0; 2020-8-18) |
| **Affiliation of Research** | The Eye and ENT Hospital of Fudan University |
| **Principal Investigator** | Huawei Li |
| **Review Mode** | ■Conference review □Express review |
| **Review Members** | See the sign-in sheets for details. |
| **Review Comments** | 1. The Ethics Committee reviewed and formally APPROVED the study protocol, granting permission for the study to proceed under the current protocol.<br>Annual/Periodic Follow-Up Reviews by the Ethics Committee: ■Yes □No<br>Frequency of Review from the Approval Date: □3 months □6 months ■12 months<br>The Ethics Committee reserves the right to adjust the frequency of annual/periodic follow-up reviews based on the actual progress of the study.<br>2. If the project is not initiated within one year from the approval date, this approval will automatically expire. |
| Signature of the Director: Gezhi Xu<br>Date: 2020.9.15<br>The Ethics Committee of the Eye and ENT Hospital of Fudan University (stamped) | |

NOTES: PLEASE READ CAREFULLY

1. The research projects approved by this Ethics Committee involve biomedical research involving human subjects and must be carried out strictly following the latest approved research protocol and informed consent form, as well as in compliance with the relevant domestic regulatory guidelines.
2. Before implementing a project that involves the export of human genetic resources or requires specific approval by relevant authorities following national regulations, the project must be declared to and approved by the relevant authorities.
3. The ethics committees of other institutions may be able to use this approval for reference purposes. If there is a different opinion regarding the protocol review, please contact the ethics committee as soon as possible.
4. Before incorporating any changes into the approved research protocol or informed consent form, the Ethics Committee must be notified on time for re-examination and approval.
5. This Ethics Committee should be notified of any severe adverse events or unintended events affecting the risk-benefit ratio of the study promptly.
6. According to the Ethics Committee's opinion regarding the frequency of annual/periodic follow-up reviews, please apply one month before the annual/periodic follow-up review date is due, regardless of whether the study has begun.
7. If there is any non-compliance with the protocol or breach of law, it must be reported immediately to the Ethics Committee.
8. In the event of a suspension or early termination of a clinical study, please inform the Ethics Committee immediately.
9. Upon completing the study, a final report must be submitted to the Ethics Committee for review.

Address: Room 305, Building 10, No. 83 Fen Yang Road, Shanghai, 200031, China;
Telephone Number: +86-021-64377134

# 复旦大学附属眼耳鼻喉科医院伦理审查批准函

声明：本伦理委员会按照国家卫计委和 CFDA 有关法规组成和工作，其审查和工作过程不受伦理委员会以外任何组织及个人影响

批件号：[2020]伦审字第（2017048-1）号

| | |
|---|---|
| 研究项目名称 | 耳鸣声治疗效果的多中心临床研究(原名称：耳鸣声治疗的临床研究) |
| 审查文件 | 修正案审查文件：<br>1. 修正案审查申请表<br>2. 修改内容清单列表<br>3. 临床研究方案（版本号：第二版；版本日期：2020 年 08 月 18 日）<br>4. 知情同意书（版本号：第二版；版本日期：2020 年 08 月 18 日） |
| 研究单位 | 复旦大学附属眼耳鼻喉科医院 |
| 主要研究者 | 李华伟 |
| 伦理审查方式 | ■会议审查 □快速审查 |
| 审查委员 | 详见“复旦大学附属眼耳鼻喉科医院伦理委员会会议签到表” |
| 审查意见 | 1. 经本伦理委员会审查，同意进行该项临床研究。<br>伦理委员会对该研究实施过程的年度/定期跟踪审查：■是 □否<br>审查频度为研究批准之日起：□3 个月 □6 个月 ■12 个月<br>伦理委员会有权根据实际进展情况改变年度/定期跟踪审查频度。<br>2. 自批准之日起一年内项目未启动，该批件自动失效。 |

主任或副主任委员签字：[illegible]

日期：2020.9.15

复旦大学附属眼耳鼻喉科医院伦理委员会（盖章）

# The Ethics Committee of the Eye and ENT Hospital of Fudan University
# Ethical Approval Form

Declaration: This ethics committee works according to the National Health Commission (NHC) and National Medical Products Administration (NMPA) regulations. The review and work processes of the ethics committee are independent.

EC Ref No. [2024LunShenZi]:2017048-2

| | |
|---|---|
| **Project Title** | **A randomized multicenter controlled clinical trial on the efficacy of sound therapy for tinnitus management (Previous title: Clinical trials of sound therapy for subjective tinnitus)** |
| **Documents for Review** | Amendments review documents:<br>1. Amendment Review Application Form<br>2. List of modified content<br>3. Clinical study protocol (Version 3.0, 2024-12-11) |
| **Affiliation of Research** | The Eye and ENT Hospital of Fudan University |
| **Principal Investigator** | Huawei Li |
| **Review Mode** | □Conference review ■Express review |
| **Review Members** | Bing Chen, Jingchao Yan. |
| **Review Comments** | 1. The clinical trial shall be conducted in compliance with the principles of Good Clinical Practice (GCP) and in accordance with the revised and approved study documents.<br>2. The frequency for annual/periodic continuing review shall remain unchanged.<br>3. This approval shall automatically become null and void if the study fails to initiate within one (1) year from the date of approval. |
| Signature of the Director: Gezhi Xu<br>Date: 2024.12.30<br>The Ethics Committee of the Eye and ENT Hospital of Fudan University (stamped) | |

NOTES: PLEASE READ CAREFULLY

1. The research projects approved by this Ethics Committee involve biomedical research involving human subjects and must be carried out strictly following the latest approved research protocol and informed consent form, as well as in compliance with the relevant domestic regulatory guidelines.
2. Before implementing a project that involves the export of human genetic resources or requires specific approval by relevant authorities following national regulations, the project must be declared to and approved by the relevant authorities.
3. The ethics committees of other institutions may be able to use this approval for reference purposes. If there is a different opinion regarding the protocol review, please contact the ethics committee as soon as possible.
4. Before incorporating any changes into the approved research protocol or informed consent form, the Ethics Committee must be notified on time for re-examination and approval.
5. This Ethics Committee should be notified of any severe adverse events or unintended events affecting the risk-benefit ratio of the study promptly.
6. According to the Ethics Committee's opinion regarding the frequency of annual/periodic follow-up reviews, please apply one month before the annual/periodic follow-up review date is due, regardless of whether the study has begun.
7. If there is any non-compliance with the protocol or breach of law, it must be reported immediately to the Ethics Committee.
8. In the event of a suspension or early termination of a clinical study, please inform the Ethics Committee immediately.
9. Upon completing the study, a final report must be submitted to the Ethics Committee for review.

Address: Room 305, Building 10, No. 83 Fen Yang Road, Shanghai, 200031, China;
Telephone Number: +86-021-64377134

# 复旦大学附属眼耳鼻喉科医院伦理审查批准函

声明：本伦理委员会按照国家卫计委和 NMPA 有关法规组成和工作，其审查和工作过程不受伦理委员会以外任何组织及个人影响

批件号：[2024]伦审字第（2017048-2）号

| | |
|---|---|
| 研究项目名称 | 耳鸣声治疗效果的多中心临床研究 |
| 审查文件 | 修正审查文件：<br>1. 修正案审查申请表<br>2. 修改内容清单<br>3. 临床研究方案（版本号：第三版；版本日期：2024 年 12 月 11 日） |
| 研究单位 | 复旦大学附属眼耳鼻喉科医院 |
| 主要研究者 | 李华伟 |
| 伦理审查方式 | ☐会议审查 ■快速审查 |
| 审查委员 | 陈兵 闫晶超 |
| 审查意见 | 1. 同意按修改后相关文件遵循 GCP 原则进行临床试验；<br>2. 年度/定期跟踪审查频率不变；<br>3. 自批准之日起一年内项目未启动，该批件自动失效。 |

主任或副主任委员签字：[signature]

日期：2024.12.[illegible]

复旦大学附属眼耳鼻喉科医院伦理委员会（盖章）

注意：（请仔细阅读）

1. 本伦理委员会批准的项目为涉及人体的生物医学研究，必须严格按照所批最新版本的研究方案和知情同意书开展研究，并遵循国内相关法规指南要求。
2. 凡是涉及人类遗传资源出口或者按照国家规定必须经有关部门专项审批的内容，均需在项目执行前向有关部门申报并获得批准。
3. 本批件可能用于其他中心伦理委员会参考，如果对方案审查存在不同意见，请及时与本伦理委员会沟通。
4. 对已批准的研究方案、知情同意书等材料的任何修改及主要研究者更换等，须及时通知本伦理委员会重新审查，获得批准后执行。
5. 发生严重不良事件及影响研究风险收益比的非预期事件，须及时报告本伦理委员会。
6. 根据伦理委员会对年度/定期跟踪审查频度的意见，无论研究开始与否，请在年度/定期跟踪审查日到期前 1 个月提出年度/定期跟踪审查的申请。
7. 发现不依从/违反方案情况须及时报告伦理委员会审查。
8. 暂停/提前终止临床研究，请及时通知伦理委员会。
9. 完成研究，须提交结题报告供伦理委员会审查。

地址：上海市汾阳路 83 号 10 号楼 305 室；邮编：200031；电话：021-64377134

# Appendix 2: Statistical Analysis

**Sample Size Calculation**
The sample size was calculated *a priori* using the "Generalized Estimating Equation (GEE) Tests for the Slope of Multiple Groups in a Repeated Measures Design" procedure implemented in the PASS module of NCSS software (Version 2021, NCSS, LLC, Kaysville, UT, USA). This method was selected to ensure sufficient power to detect differences in the rate of change (slopes) over time among the four treatment arms, which constitutes the primary study hypothesis. The calculation parameters were set as follows: a two-tailed significance level (α) of 0.05; a statistical power (1-β) of 90%; four independent groups; and six repeated measurements per participant. The time points were coded proportionally to the total study duration as 0 (T0/baseline), 0.2 (T1), 0.4 (T2), 0.6 (T3), 0.8 (T4), and 1.0 (T5). Based on preliminary data, the slopes under the alternative hypothesis were specified as -0.23, -0.24, -0.27, and -0.30 for the four groups, respectively, with a common residual standard deviation of 0.172 and an assumed exchangeable (compound symmetry) within-subject correlation structure with a coefficient of 0.5. The calculation indicated that a total sample size of 400 participants (100 per arm) was required. To account for a potential attrition rate of up to 10%, the target sample size was inflated to 440 participants.

**Handling of Missing Data**
A multi-step procedure was employed to handle missing data, which is critical for the validity of the intention-to-treat analysis. First, the mechanism of missingness was rigorously assessed. Little's test for Missing Completely at Random (MCAR) was statistically significant, leading to the rejection of the MCAR hypothesis. To evaluate whether the data were Missing at Random (MAR) rather than Missing Not at Random (MNAR), a series of logistic regression analyses were conducted. For each follow-up visit, the missingness indicator (1=missing, 0=observed) was regressed on a comprehensive set of fully observed baseline variables (e.g., age, sex, baseline THI score, tinnitus duration). The consistent lack of significant associations supported the plausibility of the MAR mechanism, justifying the use of multiple imputation.

Consequently, multiple imputation under the MAR assumption was performed using the Multivariate Imputation by Chained Equations (MICE) approach via the mice package in R. We generated 50 imputed datasets (m = 50) to achieve high efficiency, with the imputation process running for 20 iterations (maxit = 20) to ensure convergence, which was confirmed by visual inspection of trace plots. The Predictive Mean Matching (PMM) method was used for imputing the continuous outcome variables, as it preserves the original data distribution. The imputation model was fully conditional and included the following variables to make the MAR assumption more credible: all longitudinal THI scores (T0-T5), the treatment group

indicator, all pre-specified covariates (age, gender, tinnitus duration, baseline THI score), and auxiliary baseline variables (e.g., HADS-A, HADS-D, AIS, and VAS scores at baseline).

**Analysis of Baseline Characteristics**

Baseline demographic and clinical characteristics are summarized by treatment arm. Categorical variables are presented as frequencies (percentages), and group differences were assessed using Pearson's chi-square test or Fisher's exact test for small cell counts (expected frequency < 5). The normality of continuous variables was assessed using the Shapiro-Wilk test supplemented by visual inspection of Q-Q plots. Normally distributed variables are reported as mean ± standard deviation (SD) and compared across groups using one-way analysis of variance (ANOVA). Non-normally distributed variables are reported as median and interquartile range (IQR) and compared using the Kruskal-Wallis test.

**Analysis of Primary Outcome**

The primary analysis followed the intention-to-treat (ITT) principle, including all 440 randomized participants analyzed according to their original treatment assignment. Results from the 50 imputed datasets were pooled using Rubin's rules. Descriptive statistics (means and 95% confidence intervals, CIs) for the Tinnitus Handicap Inventory (THI) are presented at each time point.

The primary inferential analysis utilized a Generalized Estimating Equation (GEE) model to evaluate longitudinal changes in THI scores, accounting for the correlation of repeated measures within participants. The model included time (as a continuous variable), treatment group, the time-by-group interaction term (to test for differential slopes), and was adjusted for the pre-specified covariates of age, gender, tinnitus duration, and baseline THI score. The working correlation structure was selected by comparing the Quasi-likelihood under the Independence model Criterion (QIC) among unstructured, exchangeable, and autoregressive structures; the exchangeable structure was chosen for the primary model based on the smallest QIC value. Results are presented as parameter estimates (β) with 95% CIs and corresponding p-values for the key effects of interest, specifically the comparisons of each active intervention group (UM+NBN, HFEM, DFCRS) against the usual management (UM) control group at the primary endpoint.

**Analysis of Secondary Outcomes**

In accordance with the primary analysis strategy, all secondary outcomes—Hospital Anxiety and Depression Scale-Anxiety (HADS-A), HADS-Depression (HADS-D), Athens Insomnia Scale-8 (AIS-8), and Visual Analogue Scales for Loudness (VAS-L) and Annoyance (VAS-A)—were analyzed using an identical GEE modeling approach under the ITT principle. For each secondary outcome, a separate GEE model was fitted with the same specification as the primary THI model: including time, treatment group, the time-by-group interaction, and adjusting for the identical set of baseline covariates (age, sex, tinnitus duration, location, tone, and type). The primary

statistical inference for these outcomes focused on the estimated group effect, derived from the model, for each active intervention relative to the UM control group at the study endpoint. These model-based estimates, along with their 95% confidence intervals and p-values, provide a direct and clinically interpretable measure of the intervention's impact on anxiety, depression, insomnia, and subjective tinnitus perceptions, and are reported as the main results for the secondary outcomes in the supplementary tables (Tables S5, S7). For completeness, the omnibus test for the overall group × time interaction (across all 4 groups and 6 time points) is also reported.

**Effect Size Calculation**

Within-group effect sizes from baseline to each follow-up were calculated as Cohen's d for descriptive purposes for both primary and secondary outcomes. For each group and time point, Cohen's d was computed using the raw mean difference between the baseline and follow-up scores, divided by the pooled standard deviation (SD) of the baseline scores for the entire study sample (N=440). This approach provides a common benchmark for comparing the magnitude of change across groups and time. The 95% confidence intervals for Cohen's d were calculated using the MBESS package in R.

**Sensitivity and Supplementary Analyses**

To assess the robustness of the primary findings, several sensitivity analyses were conducted: 1) Comparison of the primary GEE model against alternative specifications (a null model and a simplified model without covariates); 2) A complete-case per-protocol (PP) analysis, which excluded participants with major protocol deviations; 3) Re-analysis using different correlation structures. The results remained consistent across these analyses, supporting the stability of the primary conclusions. An extended follow-up analysis was performed at 12 months. Furthermore, to visualize the dynamic transitions in clinical severity, a Sankey diagram was generated for the subgroup of participants with complete THI data at baseline, 3, 9, and 12 months, illustrating the flow of individuals across severity categories.

**Software and Significance**

All statistical analyses were performed using R software (version 4.2.3). Key R packages included geepack (for GEE), mice (for multiple imputation), MBESS (for effect size CIs), lubridate, tidyr, and dplyr. All statistical tests were two-tailed, and a p-value of less than 0.05 was considered statistically significant.
